# Differentiation of human induced pluripotent stem cells to authentic macrophages using a defined, serum-free, open-source medium

**DOI:** 10.1016/j.stemcr.2021.05.018

**Published:** 2021-06-24

**Authors:** Alun Vaughan-Jackson, Szymon Stodolak, Kourosh H. Ebrahimi, Cathy Browne, Paul K. Reardon, Elisabete Pires, Javier Gilbert-Jaramillo, Sally A. Cowley, William S. James

**Affiliations:** 1 Sir William Dunn School of Pathology, University of Oxford, South Parks Road, Oxford OX1 3RE, UK; 2 Chemistry Research Laboratory, Department of Chemistry, University of Oxford, Oxford OX1 3TA, UK; 3 Vagelos College of Physicians and Surgeons, Columbia University, New York, NY 10032, USA

**Keywords:** induced pluripotent stem cell, culture, differentiation, macrophage, disease modeling, transcriptional analysis, human

## Abstract

Human induced pluripotent stem cells (iPSCs) and macrophages derived from them are increasingly popular tools for research into both infectious and degenerative diseases. However, as the field strives for greater modeling accuracy, it is becoming ever more challenging to justify the use of undefined and proprietary media for the culture of these cells. Here, we describe a defined, serum-free, open-source medium for the differentiation of iPSC-derived macrophages. This medium is equally capable of maintaining these cells compared with commercial alternatives. The macrophages differentiated in this medium display improved terminally differentiated cell characteristics, reduced basal expression of induced antiviral response genes, and improved polarization capacity. We conclude that cells cultured in this medium are an appropriate and malleable model for tissue-resident macrophages, on which future differentiation techniques can be built.

## Highlights

• Transparency in composition of iPSC-derived macrophage culture medium
• Transcriptomic analysis of iPSC-derived macrophages
• Improved terminal differentiation of iPSC-derived macrophages

## Teaser

Vaughan-Jackson et al. sought to bring transparency and malleability to the composition of growth media for human iPSC-derived macrophages. They describe a new, simple, defined, and open-sourced medium which supports the growth of macrophages. Building upon previous methodologies for iPSC-derived macrophage differentiation, they improve terminal differentiation and provide fully competent authentic tissue-derived macrophages.

## Introduction

Resident phagocytes are an evolutionarily conserved cell type in metazoans. In mammals, resident macrophages support tissue homeostasis through a wide range of specialized trophic, remodeling, and defense functions, whose importance is illustrated by their failure in malignant, degenerative, and infectious diseases (Gordon et al., 2014; Steinman and Moberg, 1994). Numerous methods for the differentiation of macrophages from stem cells have been developed over the years, building upon advances in our understanding of the requirements for hematopoiesis (Rajab et al., 2018; Rowe et al., 2016). These can be broadly categorized into three types: monolayer cultures, co-cultures with xeno cells, and via embryoid body (EB) intermediates. Each vary in number of growth factors applied, timing, complexity, yield, and cost. A summary of these methodologies can be found in Table S1. To investigate the molecular pathways involved in the pathogenesis of both infectious diseases, such as those caused by human immunodeficiency virus-1 (HIV-1) (van Wilgenburg et al., 2013) and *Mycobacterium tuberculosis* (Härtlova et al., 2018), and degenerative diseases, especially Parkinson disease (Haenseler et al., 2017a; Lee et al., 2020) and Alzheimer disease (Brownjohn et al., 2018; Garcia-Reitboeck et al., 2018), we developed a pathophysiologically authentic, yet genetically tractable, model of human tissue macrophages derived from pluripotent stem cells (Karlsson et al., 2008; van Wilgenburg et al., 2013). We have shown that this model resembles an MYB-independent early wave of myelopoiesis *in vivo* that gives rise to resident macrophages in a number of tissues (Bian et al., 2020; Buchrieser et al., 2018). Our first method involved spontaneous differentiation of mesoderm from embryonic stem cells via EBs, followed by myeloid differentiation using macrophage colony stimulating factor (M-CSF) (CSF-1) and interleukin-3 (IL-3) in a serum-supplemented medium (Karlsson et al., 2008). Since this was not effective for all pluripotent stem cell lines, and to remove the undefined serum component, we subsequently developed a serum-free method, using BMP4, VEGF, and SCF to promote mesodermal lineage and prime hemogenic endothelium differentiation during EB formation, and the serum-free X-VIVO 15 (XVIVO) medium (Lonza) during the M-CSF/IL-3-directed myeloid differentiation stage (van Wilgenburg et al., 2013).

With a greater focus than ever on ensuring research integrity in the life sciences through the adoption of open practices in data curation, publication, and the sharing of materials (Cech et al., 2003; Morey et al., 2016; National Academy of Sciences, National Academy of Engineering (US) and Institute of Medicine (US) Committee on Science, Engineering, and Public Policy, 2009), it is desirable to replace proprietary media used for culturing and differentiating human pluripotent stem cells with ones that are both defined and fully open-source. While great improvements have been made to move away from xeno-material containing co-culture and serum-based methodologies toward well-defined monolayer and EB protocols, of the methods described in Table S1, only one has been described to utilize fully defined and open-sourced media throughout differentiation (Cao et al., 2019). The remainder either supplement cultures with serum, or rely on commercial, serum-free alternatives of undisclosed composition. The suppliers of both the commercial induced pluripotent stem cell (iPSC) culture medium, based on previously published medium (Chen et al., 2011), TeSR-E8 (STEMCELL Technologies), and X-VIVO 15 (Life Technologies) decline to publish the composition of these optimized materials on commercial grounds. Consequently, the scientific community does not know whether components that may have a material effect on macrophage physiology are present, nor their concentration. Examples could include, but are not limited to, anti-infectives, anti-inflammatories, cell-permeable iron-chelators cryoprotectants, and excessive concentrations of glucose. Accordingly, we have sought to base replacement media on widely available and open-source materials, enabling others to reproduce our experiments conveniently and without undue dependence on particular suppliers.

In this paper, we describe OXM, an open-source alternative macrophage differentiation medium based on Advanced DMEM/F-12 (aDMEM/F-12). We show that OXM generates homogeneous macrophage precursors very comparable to those produced by our earlier methods, both phenotypically and transcriptionally. We note that these cells show signatures consistent with more complete terminal differentiation, including improved morphology and cell-cycle arrest, and have lower basal expression levels of interferon-inducible genes while being more responsive to inflammatory stimulation. This method therefore generates cells that are even closer models of the “surveilling” state of homeostatic tissue macrophages.

## Results

### XVIVO medium, but not OXM medium, contains undisclosed molecules

We first developed a serum-free, defined, open-source medium, named OXM, for differentiation of iPSCs to macrophages. Previously, we described methodologies for differentiation (Karlsson et al., 2008; van Wilgenburg et al., 2013) in which Advanced DMEM or RPMI supplemented with 10% fetal calf serum (FCS), or a serum-free alternative, XVIVO, were used to culture the cells. However, the composition of XVIVO is proprietary, we therefore could not know whether it contained additives that may affect the phenotype of cells differentiated in this medium. We therefore decided to use aDMEM/F-12 buffered with HEPES as the base of OXM. To replace FCS, additional insulin and tropolone, a cell-permeable iron chelator shown to be a suitable substitute for transferrin in cell culture (Field, R.P., Lonza Group, 2003. Animal cell culture. US Patent 6593140), were included. M-CSF and IL-3 were supplemented independently. For full composition, see Table S2.

**Figure 1:**
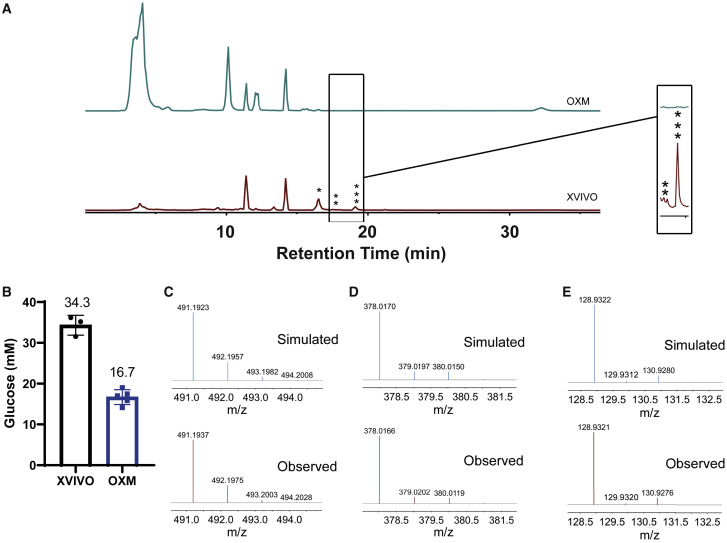
XVIVO medium, but not OXM medium, contains undisclosed molecules Negative-ion high-resolution LC-MS spectroscopy was used to analyze different compounds in the media. (A) Total ion chromatogram of the molecules in the XVIVO medium is compared with that of molecules in the OXM medium. (B) Concentration of glucose in XVIVO and OXM media. n = 3 (XVIVO) and 5 (OXM) independent experiments. Error bars represent ±SD. (C–E) (C) Comparison of the observed MS spectrum of the main peaks absent in OXM with the simulated spectrum of [M-H]^–^ adduct of isolariciresinol 9′-O-alpha-L-arabinofuranoside (^∗^), (D) that of [M + FA-H] – adduct of methyl glucosinolate (^∗∗^), and (E) that of [M-H]^–^ adduct of dithionous acid (^∗∗∗^).

To identify possible causes of differences between XVIVO- and OXM-cultured macrophages, we carried out a limited investigation into the chemical composition of each medium using high-resolution negative-ion liquid chromatography-mass spectrometry (LC-MS), specifically screening for metabolites and polar molecules, but not proteins or non-polar molecules. A comparison of the total ion chromatograms (TIC) shows that there are differences in the chemical composition between OXM and XVIVO (Figure 1A), as indicated by the intensity of individual peaks. Each peak was analyzed and putatively assigned as described in the supplemental experimental procedures. A list of predicted metabolites is given in the supplemental information (Table S3). Specifically, we noted a 2-fold difference in C6 sugar concentration between XVIVO and OXM. We subsequently independently confirmed this by enzymatic assay, finding 34.3 mM glucose in XVIVO and 16.7 mM in aDMEM/F-12 (Figure 1B). LC-MS also predicted that the levels of amino acids were at higher concentrations in OXM than in XVIVO except for tryptophan (44 nM in OXM, 6.3 μM in XVIVO) (Table S3). In addition, we noted that three peaks in the TIC of the XVIVO media were absent in that of the OXM media (Figure 1A). The best predicted molecules for these peaks are isolariciresinol 9′-O-alpha-L-arabinofuranoside (CID: 131751348, Figure 1C), methyl glucosinolate (CID: 9573942, Figure 1D) and dithinous acid (CID: 24490, Figure 1E). These molecules were either absent from the OXM media or below the detection limit.

### Differentiation of iPSCs to macrophages in novel fully defined, open-source medium, OXM

**Figure 2:**
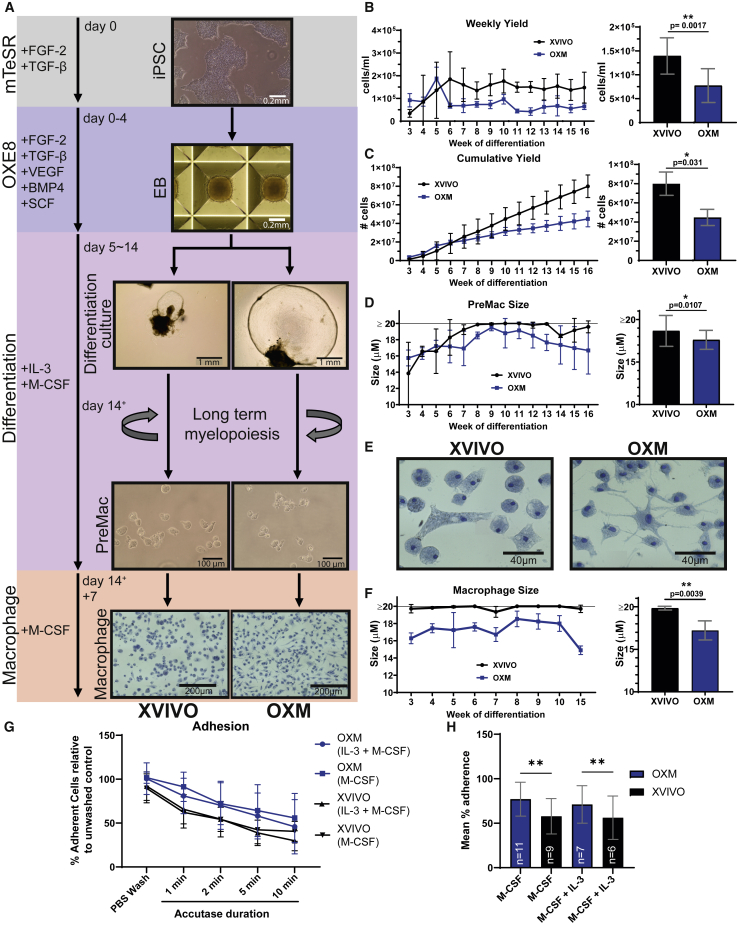
Morphology throughout macrophage differentiation (A) Schematic of iPSC-derived macrophage differentiation. Base medium is displayed on the left with main growth factor supplements adjacent. (B) Average weekly yield (left) and overall average weekly yield (right) of PreMac cells per mL of medium removed from differentiation cultures. (C) Cumulative yield of PreMac cells over 16 weeks of differentiation (left) and final total yield (right). (D) PreMac size over the lifespan of the differentiation culture (left) and overall average PreMac size (right). (E) Methylene blue (1%, w/v) staining to show macrophage morphology. (F) Macrophage size over multiple weeks of differentiation (left) and overall average macrophage size (right). (G) Percentage adherent cells cultured in fresh differentiation medium (M-CSF + IL-3) or macrophage medium (M-CSF) after incubation with Accutase normalized to untreated control. (H) Average of adherent cells over time course shown in (G). (B–H) XVIVO-cultured cells represented in black versus OXM-cultured cells in blue, mean ± SD. (B–C) n = 2 experiments in each of 3 independent donors’ cell lines. (D and F) n = 1 experiment in each of 3 independent donors’ cell lines. (G–H) n is displayed within the bars of (H) and made up from 2 independent donors’ cell lines. (B–F) Significance was calculated by Wilcoxon matched-pairs signed rank t test. (H) Significance was calculated by two-way ANOVA, Tukey’s multiple comparison test. Significance is shown when ^∗^p < 0.05, ^∗∗^ p < 0.01, ^∗∗∗^ p < 0.001.

We next sought to compare the maturation states of OXM-differentiated and cultured macrophages against those differentiated and cultured in XVIVO. EBs were formed and cultured for 4 days according to our previous publication (van Wilgenburg et al., 2013). Minor modifications to methodology were made, including culturing in an in-house, defined, open-source medium derived from E8 (Chen et al., 2011) we call OXE8. Note that, while the published composition is open-source, commercial media based on E8 have been modified and have proprietary compositions. OXE8 contains FGF-2 and transforming growth factor β and further supplements of VEGF, BMP4, and SCF (for composition see Table S2). After EB formation, as in our earlier methods, EBs were transferred into OXM- or XVIVO-based differentiation medium supplemented with IL-3 and M-CSF. For terminal macrophage differentiation media, IL-3 and tropolone were then omitted (Figure 2A; Table S2). Morphological differences between differentiation cultures were immediately apparent. EBs produced larger cyst-like structures and greater adherent stromal growth in OXM medium (Figure 2A). By week 3 of differentiation, monocyte-like macrophage precursors cells (PreMacs) had started to be released into the supernatant. This continued for >16 weeks (Figures 2B and 2C). Interestingly, OXM differentiation cultures produced a large number of PreMacs in the first 3 weeks of production before their yield reduced to 7–8 × 10^4^ cells/mL/week. XVIVO differentiation cultures were slower to yield PreMacs, but by week 6 produced approximately 2-fold more cells, an average of 1.4 × 10^5^ cells/mL/week (Figure 2B). After 16 weeks the average total yield was (4.48 ± 1.26) × 10^7^ cells in OXM and (7.99 ± 2.61) × 10^7^ cells in XVIVO (Figure 2C). PreMacs produced in OXM were significantly smaller than in XVIVO (range 15.8–19.5 μm, mean 17.9 μm, versus 13.8 to ≥20 μm, mean 19.5 μm, respectively) (Figure 2D). Note that owing to limitations of the NucleoCounter NC-3000, 20 μm is the upper limit of cell size, so XVIVO-produced cells may have been larger.

For terminal differentiation of PreMacs into macrophages, cells were cultured for a further 7 days in macrophage differentiation medium. After 7 days, XVIVO-cultured cells remain rounded or acquire a bipolar morphology, with large vesicle-like structures visible. OXM-cultured cells were morphologically heterogeneous with rounded cells, large, flattened cells, and cells with multiple projections (Figure 2A, expanded view in 2E). The size of adherent macrophages was measured after resuspension. OXM-cultured macrophages were significantly smaller than those in XVIVO: mean 17.51 μm (range 16.28–18.53 μm) versus mean 19.85 μm (range 19.35 to ≥20 μm) (Figure 2F). Differences in time taken to resuspend OXM versus XVIVO cells showed that OXM cells were more adherent, both when cultured in macrophage medium or in fresh differentiation medium (Figures 2G and 2H). In summary, OXM supports the production of more adherent, smaller macrophages than XVIVO, albeit with a lower cumulative yield.

### Macrophages cultured in OXM are phenotypically similar to, but distinct from, XVIVO-cultured cells

**Figure 3:**
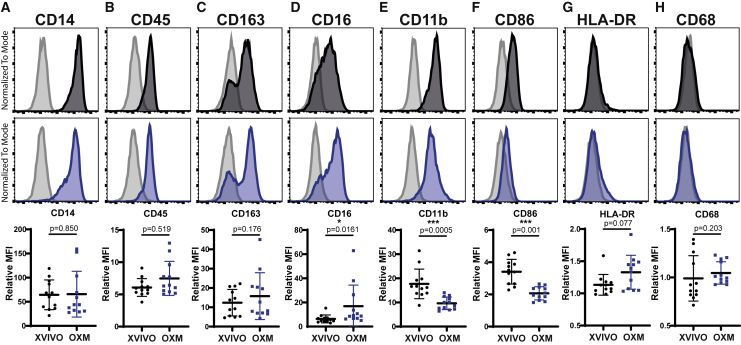
Macrophage surface marker phenotype Surface expression of (A) CD14, (B) CD45, (C) CD163, (D) CD16, (E) CD11b, (F) CD86, (G) HLA-DR, and (H) CD68 as measured by flow cytometry on macrophages. Histograms show fluorescence intensity (x axis) normalized to the mode (y axis) for macrophages cultured in XVIVO (black) or OXM (blue), relative to the isotype control (gray). Dot plots show ratio of the geometric mean fluorescence intensity (MFI) compared with the isotype control. Bars display mean ± SD. n = 4 experiments in each of 3 independent donors’ cell lines. Significance was calculated by Wilcoxon matched-pairs signed rank t test. Significance is shown when ^∗^p < 0.05, ^∗∗^ p < 0.01, ^∗∗∗^ p < 0.001.

To determine macrophage phenotype, surface marker abundance was determined by flow cytometry. Key markers of macrophage lineage, the lipopolysaccharide (LPS) co-receptor CD14 (Figure 3A), and the pan-leukocyte marker CD45 (Figure 3B), were highly expressed in macrophages derived using both media, with no significant differences. Nor was a difference observed with the expression of scavenger receptor CD163 (Figure 3C). Levels of the Fcγ immunoglobulin G (IgG) receptor, CD16, were significantly higher in OXM-cultured cells (Figure 3D). Conversely, both the β2-integrin receptor, CD11b, and the costimulatory receptor, CD86, were more highly expressed in XVIVO-cultured cells, suggesting a more M1-like phenotype (Mosser, 2003) (Figures 3E and 3F). Consistent with our previous reports (Haenseler et al., 2017b; Karlsson et al., 2008; van Wilgenburg et al., 2013), major histocompatibility complex class II (human leukocyte antigen-DR [HLA-DR]) expression is low in these unstimulated cells in either condition (Figure 3G). Expression of the tissue-resident macrophage marker CD68 was observed intracellularly but not on the cell surface, consistent with earlier literature (Gordon and Plüddemann, 2017), and was significantly higher in OXM-cultured cells (Figures 3H and S1H).

PreMac cells gave broadly similar results to those of fully differentiated macrophages, although with differences in CD14 and CD16 expression (Figure S2). Overall, the PreMacs and macrophages produced using both OXM and XVIVO media display a macrophage phenotype, but show small and consistent differences that may reflect differences in polarization.

Finally, to test the phagocytic capacity of macrophages produced under the two conditions, we measured phagocytic uptake of Alexa 488-conjugated zymosan, a yeast-derived particulate glycan. Phagocytic uptake after 30 min was not significantly different between conditions across three genetic backgrounds (Figure S3), indicating that macrophages cultured in OXM are phagocytically competent.

### While transcriptionally very similar, OXM-cultured macrophages have a more homeostatic, and XVIVO-cultured macrophages a more immunologically active, signature

**Figure 4:**
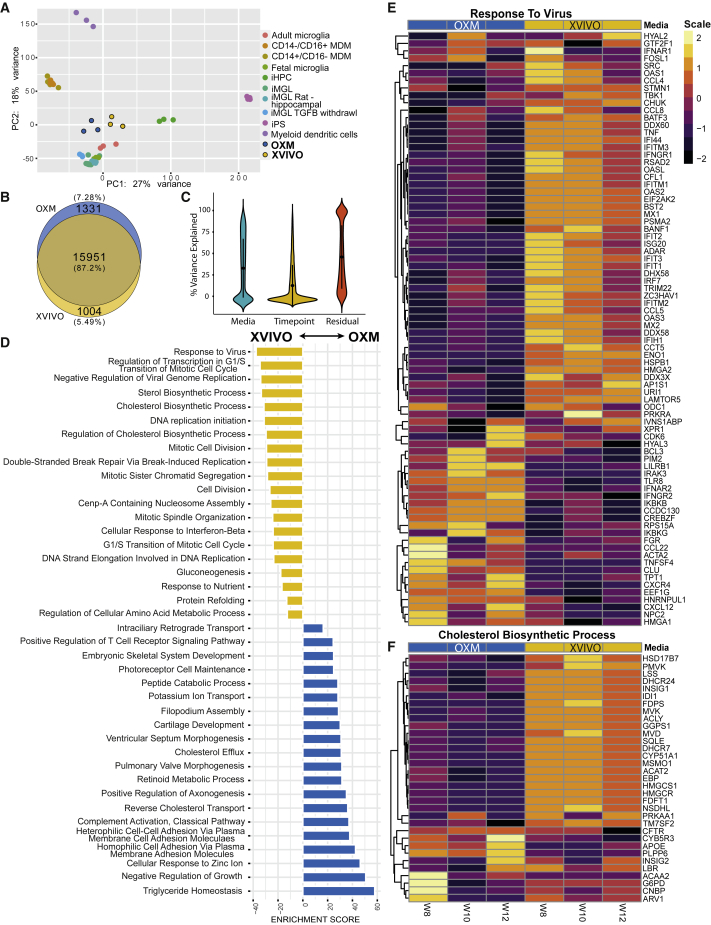
Transcriptome of iPSC-derived macrophages Transcriptome of macrophages as measured by RNA-seq of three biological replicates per medium condition from one genetic background (SFC840-03-03). (A) Principal-component analysis (PCA) plot of transcriptional profile for iPSC-derived macrophages cultured in XVIVO or OXM compared with iPSC, induced hematopoietic progenitor cells (iHPCs), microglia (MGL) (adult, fetal, iPSC-derived, and iPSC-derived with TGFB withdrawal), monocytes (MDM), and dendritic cells reported previously (Abud et al., 2017). (B) Venn diagram representing total number of genes expressed by iPSC-derived macrophages in either medium composition. (C) Violin plot of the percentage variance attributed to each variable (medium, time point, and residual). (D) GO term enrichment between media compositions. Top 15 most enriched results in each medium are shown. Yellow represents terms enriched in XVIVO-cultured cells, and blue in OXM-cultured cells. (E and F) Heatmaps of GO terms for; “response to virus” and “cholesterol biosynthetic process.” Results across three repeat measurements are shown. XVIVO in yellow, OXM in blue. The color in each row represents the *Z* score of the log_2_ fold difference from the mean TPM value for that gene.

We used RNA sequencing (RNA-seq) to investigate the detailed expression profile of our iPSC-derived tissue macrophages. We first compared the macrophages cultured in XVIVO or OXM to a published dataset of iPSC-derived human microglia (tissue-resident macrophages of the brain) that had been differentiated via induced hematopoietic progenitor cells (iHPCs) derived using modified protocols from previous reports (Abud et al., 2017). Principal-component analysis, in which PC1 explained 27% of the variance, and PC2 explained 18%, showed tight clustering of OXM- and XVIVO-cultured cells together. The iPSC-derived macrophages showed closest similarity to microglia rather than iHPCs or monocyte-derived macrophages (MDMs), and least similarity to pluripotent cells and definitive hematopoietic lineages, such as dendritic cells (Figure 4A). This was expected because iPSC macrophages have a primitive ontogeny, similar to that of microglia, which develop from primitive, yolk sac/embryonic fetal macrophages, unlike adult definitive MDMs that develop from hematopoietic progenitors in the bone marrow (Buchrieser et al., 2017; Ginhoux and Guilliams, 2016; Vanhee et al., 2015). Further differential expression analysis found 15,951 genes shared in both OXM and XVIVO populations, and 2,335 genes unique to either OXM (1,331) or XVIVO (1,004) (Figure 4B). Analysis of the sources of this variation showed that the media used explained approximately 30% of the variation between samples, while the age of the differentiation culture (as indicated by the week of PreMac harvesting) accounted for approximately 15%, and the remainder was due to residual, undefined sources (Figure 4C).

The three genes most significantly upregulated in OXM versus XVIVO were the carbohydrate binding lectin, *CHI3L1*, the tetraspanin, *CD37*, and the protease, *HTRA4* (p = 0.0028, 0.0049, and 0.006, respectively). In XVIVO, the most upregulated genes were the interferon-inducible protein *IFIT3*, the endonuclease *RNASE1*, and the epoxidase *SQLE* (p = 0.0028, 0.006, and 0.006, respectively). The most enriched gene ontology (GO) term in XVIVO compared with OXM-cultured cells, was “response to virus” (Figures 4D and 4E). Multiple interferon-inducible genes, e.g., *MX2*, *RSAD2*, and the *IFIT* family of proteins, were more highly expressed in XVIVO-cultured cells. However, some antiviral receptors, such as *TLR8*, are more highly expressed in OXM-cultured cells. We also found enrichment of metabolism-related processes. In OXM-cultured cells, the most highly enriched GO term was “triglyceride homeostasis” (Figure S4A), and in XVIVO-cultured cells the “cholesterol biosynthetic process” was highly enriched with 23/33 genes in this term upregulated in these cells (Figures 4D and 4F). The term “DNA replication initiation” was enriched in XVIVO-cultured cells (Figure S4B). To test whether XVIVO cells may therefore be in cell cycle, we measured total KI67, which transcriptionally has 2.66-fold higher expression in XVIVO-cultured cells (Figure S4C), by immunostaining and flow cytometry. This confirmed expression is higher but note that only a small proportion of cells (XVIVO; 1.15% ± 0.90%, OXM; 0.54% ± 0.55%) are in cell cycle (Figure S4D). In OXM, the inflammation-related term “complement activation, classical pathway,” cell adhesion, and several developmental terms are enriched (Figures 4E and S4E). The enrichment of the cell adhesion term supports observations that OXM-cultured cells were more adherent than XVIVO-cultured cells (Figures 2G and 2H). Overall, when compared against adult microglia, both OXM- and XVIVO-cultured cells show similar but distinct expression profiles of genes within the GO terms mentioned above (Figure S5). However, OXM macrophages, in particular, display closer levels of expression of genes regulating the cell cycle and cholesterol metabolism compared with microglia (Figures S5C and S5D). Transcriptomic analysis has shown that, although cells cultured in these different media are highly similar, XVIVO-cultured macrophages display a greater tendency toward proliferation and an activated antiviral response, and OXM-cultured macrophages toward homeostasis, adhesion, and inflammation.

### OXM- and XVIVO-cultured macrophages have different classical and alternative activation profiles

**Figure 5:**
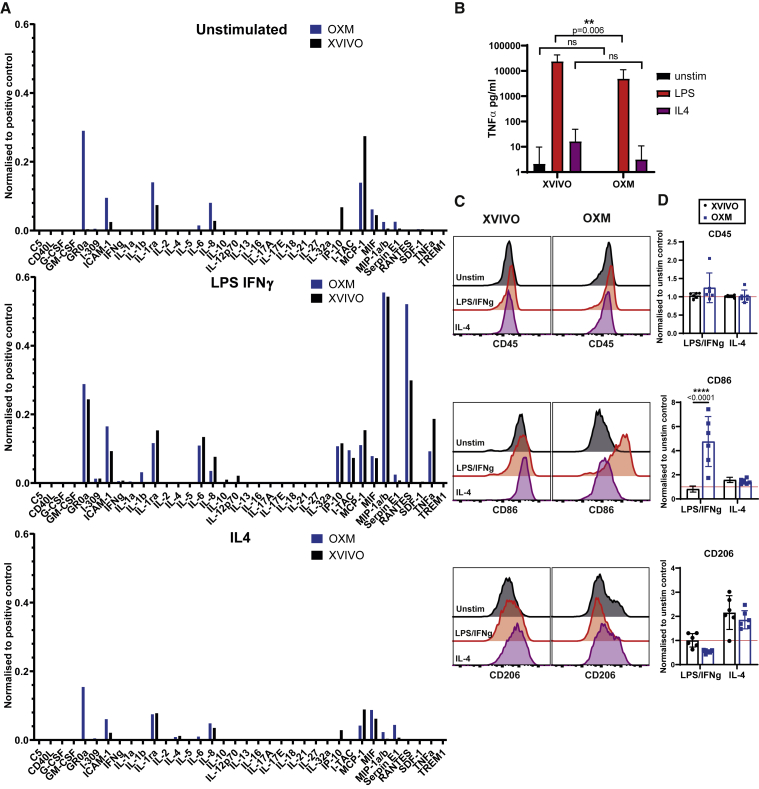
Macrophage activation states between culture media (A) Cytokine secretion into the supernatant under resting conditions (top), or stimulated with 100 ng/mL LPS + 20 ng/mL IFN-γ (middle) or 50 ng/mL IL-4 (bottom) normalized to a positive control. n = 1 experiment. (B) TNF-α secretion from unstimulated (black), or 100 ng/mL LPS (red) or 50 ng/mL IL-4 (purple) stimulated cells. Mean ± SD, n = 5 experiments in each of 3 independent donors’ cell lines. (C) Surface expression of CD45, CD86, or CD206 measured by flow cytometry after stimulation, colored according to (B). Histograms show fluorescence intensity (x axis) normalized to the mode (y axis). (D) Geometric MFI relative to unstimulated control. Mean ± SD, n = 3 experiments in each of 2 independent donors’ cell lines. (B–D) Significance was calculated by two-way ANOVA, Sidak’s multiple comparison test. Significance is shown when ^∗^p < 0.05, ^∗∗^p < 0.01, ^∗∗∗^p < 0.001, ^∗∗∗∗^p < 0.0001.

To test whether the subtle transcriptomic differences between the cells differentiated in the two media resulted in functional differences, we measured cytokine secretion and polarization after stimulation. Cytokine secretion was measured at resting state and after classical (LPS and INF-γ) or alternative (IL-4) activation, as described previously (Jiang et al., 2012; van Wilgenburg et al., 2013). Cytokine profiles under both conditions were consistent with the previous reports (Figure 5A). OXM-cultured cells produced noticeably more GROα, ICAM-1, IL-1ra, and IL-8 in the resting state, while XVIVO-cultured cells produced more IP-10 (CXCL10). After classical activation, XVIVO-cultured cells produced more tumor necrosis factor alpha (TNF-α), as well as low levels of IL-12p70, while OXM-cultured cells produced more RANTES (CCL5) and small quantities of IL-1β. Alternative activation suppressed secretion of resting state cytokines in both media. Consistent with the results above, LPS-induced TNF-α secretion from OXM-cultured cells quantified by enzyme-linked immunosorbent assay (ELISA) was lower than from XVIVO cells (Figure 5B).

Considering the differences in cytokine production, we next assessed macrophage surface marker polarization toward classically defined M1 and M2 states after activation. This was measured by immunostaining for CD86 and CD206, respectively (Mosser, 2003; Roszer, 2015) (Figures 5C and 5D). Although activation had no effect on CD45 expression in both conditions, CD86 expression significantly increased in OXM-cultured cells compared with XVIVO-cultured cells after classical activation (4.75- versus 0.81-fold). CD206 expression increased in both conditions after alternative activation and was not significantly different between conditions (1.86- versus 2.16-fold) (Figures 5C and 5D). In conclusion, although cells cultured under either condition can respond well to stimuli, they differ in their cytokine profile, and cells cultured in OXM display greater changes in typical macrophage polarization surface markers than those in XVIVO.

### OXM-cultured macrophages are more susceptible to HIV-1 infection, but not to Zika virus

**Figure 6:**
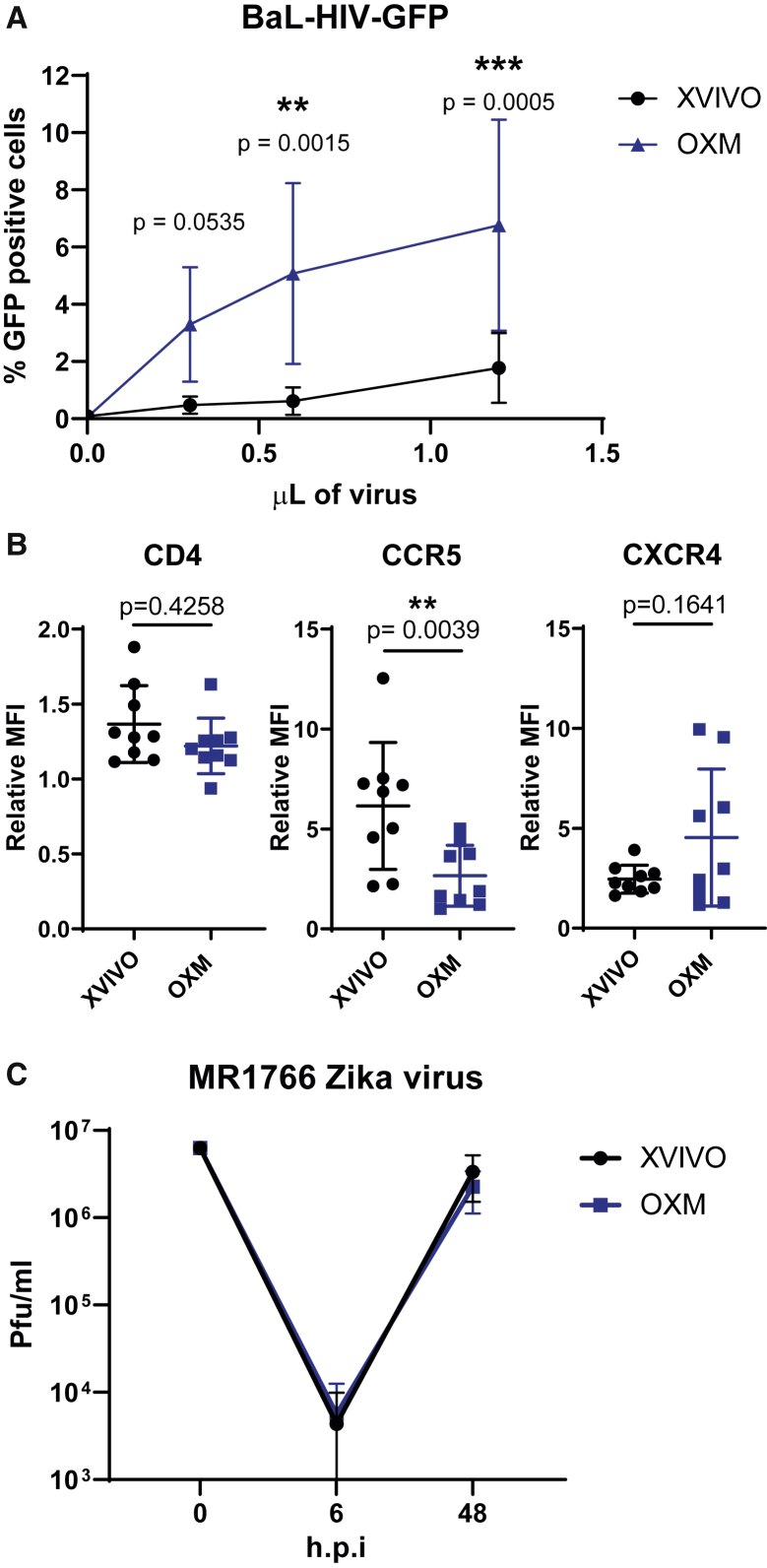
Macrophage infection by HIV-1 or Zika virus under different culture conditions (A) Percentage of macrophages infected with a GFP-encoding, single-round HIV-1 pseudotyped virus measured by flow cytometry. (B) CD4, CCR5, and CXCR4 surface expression measured by flow cytometry shown as ratio of geometric MFI to the isotype control. (C) MR1766 Zika virus titer from infected macrophages shown in plaque-forming units (pfu)/mL. Significance was calculated by (A) two-way ANOVA, Sidak’s multiple comparison test, (B) Wilcoxon matched-pairs signed rank t test. (A–C) Mean ± SD n = 4 experiments in each of 2 independent donors’ cell lines. Significance is shown when ^∗^p < 0.05, ^∗∗^ p < 0.01, ^∗∗∗^ p < 0.001.

Due to their positioning as sentinel cells throughout the body, tissue-resident macrophages are often the first members of the immune system to respond to infection. Consequently, several pathogens have evolved to use these cells as a host during early infection, including viruses, such as HIV-1 and Zika virus (ZIKV). In the case of HIV-1, macrophages are hypothesized to act as a potential reservoir for latent infection due to the long-lived, non-replicating state of these cells (Honeycutt et al., 2016, 2017). To determine whether iPSC macrophages would be a suitable model for modeling this disease, macrophages were infected with a macrophage-tropic (CCR5-tropic Ba-L envelope), GFP expressing, HIV-1 pseudotype virus. Infectivity was measured by expression of GFP after 72 h by flow cytometry. A significantly higher proportion of macrophages cultured in OXM than in XVIVO were infected (6.76% ± 3.69% compared with 1.77% ± 1.23%; Figure 6A). Expression of the HIV-1 entry receptors CD4 and CXCR4 was not significantly different between conditions, and CCR5 was higher in XVIVO-cultured macrophages (mean flouresence intensity (MFI) relative to isotype: 6.16 ± 3.18 XVIVO versus 2.67 ± 1.52 OXM; Figure 6B). This difference in viral entry may be explained by the higher expression of known HIV-1 restriction factors in XVIVO-cultured cells, including the *IFITM* and the *APOBEC* families of proteins, and *TRIM5α* (Figure 4F). To test whether differences in susceptibility are restricted to lentiviruses, we also assessed replication ZIKV strain MR-766 (Uganda, 1947) in these cells. Cells produced in both media released virus after an eclipse phase of ~6 h post infection with no difference in output titer ([3.36 ± 1.83] ×10^7^ pfu/mL XVIVO, [2.26 ± 1.14] ×10^7^ pfu/mL OXM at 48 h post infection; Figure 6C). Therefore, OXM-cultured macrophages are a good model for HIV-1 infection, and differences in viral susceptibility between culturing conditions are not pan-viral.

## Discussion

The need to define an open-source medium for differentiating macrophages from iPSC was driven by two requirements. Firstly, to understand the finer changes in macrophage response to stimuli we must have a clear understanding of the resting conditions of these cells. Secondly, LC-MS analysis indicated the potential presence of several undisclosed molecules in the commercial serum-free alternative medium, XVIVO 15, some of which could inhibit, or polarize the macrophage response if present. Our analysis putatively identified isolariciresinol 9′-O-alpha-L-arabinofuranoside, a lignan glycoside, which is known to have anti-inflammatory effects (Liu et al., 2018), as well as methyl glucosinolate and dithionous acid. While the precise functions of these molecules are unknown, it is worth noting that indole glucosinolates have been reported to have potent anti-inflammatory effects (Vo et al., 2014). In its ionized form (dithionite), dithionous acid is a strong reducing agent capable of affecting the redox state of heme-containing enzymes, such as cytochrome *b*, which may affect cell physiology (Berton et al., 1986).

We found that basing our new medium, OXM, on aDMEM/F-12, did not prevent differentiation of macrophages from iPSCs. However, we did see considerable differences in both yield and morphology. OXM-cultured cells were significantly more adherent, with the increased expression of adherence-related genes. This appeared to limit the harvestability of the cells cultured in OXM to once per week, most probably due to inducing cell adherence to plastic seen when cultured in fresh differentiation medium, but does not explain the observed reduced yield of macrophages cultured in this medium. We have previously shown that XVIVO-cultured iPSC macrophages are significantly larger than blood MDMs (van Wilgenburg et al., 2013), which are closer in size to OXM-cultured cells. Considering the very high levels of glucose in XVIVO medium, we suspected that there could be major differences in cell metabolism. We were therefore unsurprised to see that metabolism-related GO terms were among the most abundant terms differentially enriched between the media types. Whereas OXM-cultured cells were enriched for triglyceride homeostasis, reverse cholesterol transport and peptide catabolic process, XVIVO-cultured cells upregulated many genes involved in lipid metabolism and cholesterol biosynthesis, such as fatty acid synthase (*FASN*), 7-dehydrocholesterol reductase (*DHCR7*), and acetyl-CoA acetyltransferase 2 (*ACAT2*).

Macrophage metabolism differs substantially between activated states (M1 versus M2), with fatty acid synthesis upregulated in M1 cells and fatty acid oxidation upregulated in M2 cells (Caputa et al., 2019). Unsurprisingly then, we observed that XVIVO-cultured cells had higher expression of markers of classical M1 (pro-inflammatory) polarization (Boyette et al., 2017; Mosser, 2003). It is possible that this activated state in XVIVO is responsible for their relative insensitivity to LPS and interferon γ-induced changes in CD86 expression, unlike OXM-cultured cells, which displayed significant changes in this marker, although this does not appear to impact the cytokine response by these cells. Conversely, OXM-cultured cells showed similarities to non-classical or inflammatory monocytes, with increased expression of CD16. This potential pro-inflammatory phenotype may therefore be linked to the constitutive secretion of some inflammatory cytokines, such as IL-6 and GROα, but its importance in cellular function is unclear. Nevertheless, this cytokine profile observed is consistent with our previous work (Haenseler et al., 2017b) in which iPSC-derived microglia released similar cytokines in monoculture that were reduced when co-cultured with neurons. This suggests that our tissue-resident macrophages likely receive anti-inflammatory signals from neighboring stromal cells as part of a regulatory feedback system. Overall, neither resting OXM- or XVIVO-cultured macrophages clearly fell into conventional phenotypes, such as “classical,” “non-classical,” “M1-activated,” or “M2-activated.” However, iPSC-derived macrophages have been shown to more closely resemble primitive macrophages, and therefore such categories, which are largely derived from work with adult blood MDMs cultured in serum-containing medium, are an imperfect system for the phenotypic characterization of these cells (Murray et al., 2014; Nahrendorf and Swirski, 2016). Instead, primitive macrophages, such as our iPSC-derived macrophages (Buchrieser et al., 2017), ought to be considered more along a spectrum of activation states, subtly influenced by nutrient availability and extracellular matrix and cell-cell interactions in the various tissue environments they may occupy (Guilliams et al., 2020; Martinez and Gordon, 2014; Murray et al., 2014).

One surprising observation was that XVIVO-cultured cells appear to be transcriptionally primed for an antiviral response. We found higher expression of many interferon-inducible genes and constitutive secretion of the interferon-inducible cytokine IP-10 (CXCL10). This clearly translated into a reduced infectivity of HIV-1 pseudotype virus in these cells compared with OXM-cultured cells, despite the cells having higher expression of the surface receptor CCR5. However, it did not result in a difference in ZIKV infection of these cells. This is supported by the fact that OXM-cultured cells had higher expression of the interferon receptors *IFNAR2* and *IFNGR2*, and therefore are likely capable of mounting a strong antiviral response. It is worth noting that, although expression of interferon genes was lower in OXM-cultured cells, it was not absent. These cells may therefore be a better model of pre-viral exposure macrophages.

To conclude, we describe the successful development of a defined, and open-source medium for culturing human iPSC-derived macrophages: OXM. Subtle differences in phenotype and function in macrophages cultured in a commercial alternative highlight the importance of transparency in culturing techniques and demonstrates how direct comparisons without validation between culturing methods may not always be appropriate. We hope that the open-source nature of this medium will enable it to be used as a foundation for future improved differentiation protocols not only for macrophages, but also other iPSC-derived cell types.

## Experimental procedures

### iPSC lines

The derivation and characterization of the iPSC lines used in this study are described elsewhere: SFC840-03-03 (Fernandes et al., 2016), SFC841-03-01 (Dafinca et al., 2016), and SFC856-03-04 (Haenseler et al., 2017b). See supplemental information for more information.

### iPSC culture

iPSCs were cultured in their stated medium (Table S1) on Geltrex (Gibco, no. A1413201)-coated tissue culture dishes and passaged using TrypLE Express (Gibco, no. 12604013). For 24 h after plating, medium was supplemented with 10 μM Y-27632 (Abcam, ab120129). Cells were incubated at 37°C, 5% CO_2_. See supplemental information for more information.

### Macrophage differentiation

iPSCs were differentiated into macrophages using a protocol based on that described previously (van Wilgenburg et al., 2013). The updated method is as follows. AggreWell 800 plates (STEMCELL Technologies, no. 34815) were prepared by addition of 0.5 mL Anti-Adherence Rinsing Solution (STEMCELL Technologies, no. 07010) and centrifugation at 3,000 × *g* for 3 min to remove bubbles from the microwells. Rinse solution was then aspirated and replaced with 1 mL of 2× concentrated EB medium (1× EB medium: OXE8 medium supplemented with 50 ng/mL BMP4 (PeproTech, no. PHC9534), 50 ng/mL VEGF (PeproTech, no. PHC9394), and 20 ng/mL SCF (Miltenyi Biotec, no. 130-096-695) supplemented with 10 μM Y-27632. iPSCs were resuspended by washing with PBS, incubating in TrypLE Express for 3–5 min at 37°C, 5% CO_2_, followed by gentle lifting in aDMEM/F-12 to achieve single-cell suspension. Cells were counted and pelleted by centrifugation at 400 × *g* for 5 min. After centrifugation, cells were resuspended at 4 × 10^6^ cells/mL in OXE8 supplemented with 10 μM Y-27632 and 1 mL added to the AggreWell. The AggreWell plate was then spun at 100 × *g* for 3 min with no braking to encourage even distribution of cells across microwells. Cells were incubated for 4 days at 37°C, 5% CO_2_, with daily feeding of 75% medium change with EB medium, by aspiration of 1 mL by pipette, and gentle addition of 1 mL fresh medium twice to avoid disturbance to the microwells. After 4 days, EBs were lifted from the plate using a Pasteur pipette and passed over a 40 μm cell strainer to remove dead cells, before washing into a tissue culture plate with differentiation medium (Table S2). EBs were divided evenly into 2 T175 flasks and topped up to 20 mL with differentiation medium. Differentiation cultures were incubated at 37°C, 5% CO_2_, with weekly feeding of an additional 10 mL until macrophage precursors (PreMac) cells started to be produced. After this point, PreMac cells were collected weekly and a minimum of equal volumes of media to the volume removed were replaced into the differentiation cultures. Each harvest involved a 25%–50% medium change. Harvested cells could be stored or transported on ice for up to 48 h without loss of viability, and were either used directly or plated in appropriately sized tissue culture plates for further culturing in XVIVO or OXM macrophage medium (Table S2) for a further 7 days, with a 50% medium change on day 4.

### Cell count, size, and viability measurements

Cell count, size, and viability were measured using the NucleoCounter NC-3000 (ChemoMetec) after staining for live/dead cells using Solution-13 AO-DAPI stain (ChemoMetec, no. 910-3013). See the supplemental information for more details.

### Cell adhesion

Cells were lifted by incubation with Accutase and remaining adherent cells stained with NucBlue for imaging and quantification of nuclei by fluorescent microscopy. See the supplemental information for more details.

### Flow cytometry

Cells were stained directly without fixation in fluorescence-activated cell sorting buffer (PBS supplemented to 1% FBS, 10 μg/mL human-IgG (Sigma, no. I8640-100MG), and 0.01% sodium azide) and compared with isotype controls with the same fluorophores from the same company. Fluorescence was measured using the BD LSRFortessa X-20 (BD Biosciences) and analyzed on FlowJo version 10. See the supplemental information for more details.

### Cytokine and chemokine release

Cytokine and chemokine secretion was assessed as described previously (van Wilgenburg et al., 2013) using the Proteome Profiler Human Cytokine Array Kit (R&D Systems, no. ARY005B). TNF-α was quantified by ELISA (Invitrogen, 88-7346-88) according to manufacturer’s instructions. See the supplemental information for more details.

### Phagocytosis

Phagocytosis was measured by uptake of zymosan A (*S. cerevisiae*) BioParticles Alexa Fluor 488 (Thermo Fisher Scientific, no. Z23373) after 30 min. Uptake was measured by measurement of fluorescence using the BD LSRFortessa X-20. See the supplemental information for more details.

### HIV-1 infection assay

Seven-day differentiated macrophages were infected for 72 h with a GFP-expressing HIV-1 lentiviral vector pseudotyped with strain Ba-L envelope virus diluted in macrophage medium. Percentage infected cells was determined by measurement of fluorescence using the BD LSRFortessa X-20 flow cytometer. See the supplemental information for more details.

### ZIKV infection assay

Macrophages were infected with 2 × 10^6^ pfu of ZIKV isolate MR1766 for 4 h before replacing with fresh medium. Supernatant was collected at the stated time points and stored for later quantification of virus by plaque assaying on Vero-76 cells. See the supplemental information for more details.

### RNA-seq

RNA was extracted from 1 × 10^6^ macrophages cultured in a 6-well plate, differentiated from PreMac cells harvested at weeks 8, 10, and 12 of differentiation, and sequenced by Novogene. Results were analyzed in-house.

### LC-MS analysis of media

Samples of the media were prepared for LC-MS analysis as described previously (Ebrahimi et al., 2020). See the supplemental information for more details.

### Quantification of glucose concentration

Glucose concentration in the media was quantified using a modified protocol for the Glucose (HK) Assay Kit (Supelco, no. GAHK20-1KT). See the supplemental information for more details.

### Statistical analysis

All statistical analysis used is reported in the figure legends and was carried out using GraphPad Prism version 8.2.1 or R version 4.0. In all cases data were considered significant when p < 0.05.

### Data and code availability

RNA-seq data and analysis have been deposited with the NCBI Gene Expression Omnibus. Theaccession number for the data in this paper is GEO: GSE171313. See the supplemental information for more details.

## Author contributions

Conceptualization, A.V.-J., S.A.C., and W.S.J.; methodology, A.V.-J. and W.S.J.; investigation, A.V.-J., K.H.E., C.B., E.P., J.G.-J., and W.S.J.; data curation, S.S.; formal analysis, A.V.-J., S.S., and K.H.E.; visualization, A.V.-J., S.S., K.H.E., and P.K.R.; supervision, P.K.R. and E.P.; writing – original draft, A.V.-J., K.H.E., and W.S.J.; writing – review & editing, A.V.-J., S.A.C., and W.S.J.; funding acquisition, A.V.-J., S.A.C., and W.S.J.

## Supplemental information

Document S1.Supplemental experimental procedures, Figures S1–S5, and Tables S1–S3

Document S2. Article plus supplemental information

## Footnotes

Supplemental information can be found online at https://doi.org/10.1016/j.stemcr.2021.05.018.
