# Differentiation of human induced pluripotent stem cells to authentic macrophages using a defined, serum-free, open-source medium

**DOI:** 10.1016/j.stemcr.2021.11.010

**Published:** 2021-12-14

**Authors:** Alun Vaughan-Jackson, Szymon Stodolak, Kourosh H. Ebrahimi, Cathy Browne, Paul K. Reardon, Elisabete Pires, Javier Gilbert-Jaramillo, Sally A. Cowley, William S. James

## Main text

(Stem Cell Reports *16*, 1735–1748; 2021)

The authors would like to make two corrections to the supplemental information for this manuscript. First, the formulation of XVIVO medium is stated to be the same as previously published by van Wilgenburg et al. (2013), and OXM is subsequently based on this formulation. In van Wilgenburg et al. (2013) the concentration of M-CSF used was 100 ng/mL; however, in the originally published version of our supplemental information, 50 ng/mL M-CSF was reportedly used. This value was incorrect as written, and the concentration was in fact 100 ng/mL, the same as in Wilgenburg et al. (2013). Therefore, Table S2 in the supplemental information has been corrected to reflect this, stating a final concentration of 100 ng/mL M-CSF in both OXM and XVIVO media. Secondly, in the originally published version of this paper, OXM Macrophage medium is described to be the same as OXM differentiation medium with only Tropolone and IL-3 omitted. However, in the originally published version of Table S2 in the supplemental information, the composition of OXM Macrophage medium is listed as 96.5% Advanced DMEM/F-12, 2mM GlutaMAX, 15mM HEPES, 10 ng/mL M-CSF, and 1% Penicillin-Streptomycin. The correct formulation is 96.5% Advanced DMEM/F-12, 2mM GlutaMAX, 15mM HEPES, 5 μg/mL human recombinant Insulin solution, 100 ng/mL M-CSF, and 1% Penicillin-Streptomycin. The supplemental information has now been corrected with the manuscript online and the authors sincerely apologize for any confusion these errors may have caused.
