# Supplementary material for: Differentiation of human induced pluripotent stem cells to authentic macrophages using a defined, serum-free, open-source medium
Source: Stem Cell Reports. 2021 Jun 24;16(7):1735–48. doi: 10.1016/j.stemcr.2021.05.018 (PMC8282471; doi:10.1016/j.stemcr.2021.05.018)
Supplement: Document S1.Supplemental experimental procedures, Figures S1–S5, and Tables S1–S3 [file mmc1.pdf]

Stem Cell Reports, Volume *16*

Supplemental Information

Differentiation of human induced pluripotent stem cells to authentic macrophages using a defined, serum-free, open-source medium

Alun Vaughan-Jackson, Szymon Stodolak, Kourosh H. Ebrahimi, Cathy Browne, Paul K. Reardon, Elisabete Pires, Javier Gilbert-Jaramillo, Sally A. Cowley, and William S. James

Supplemental Items

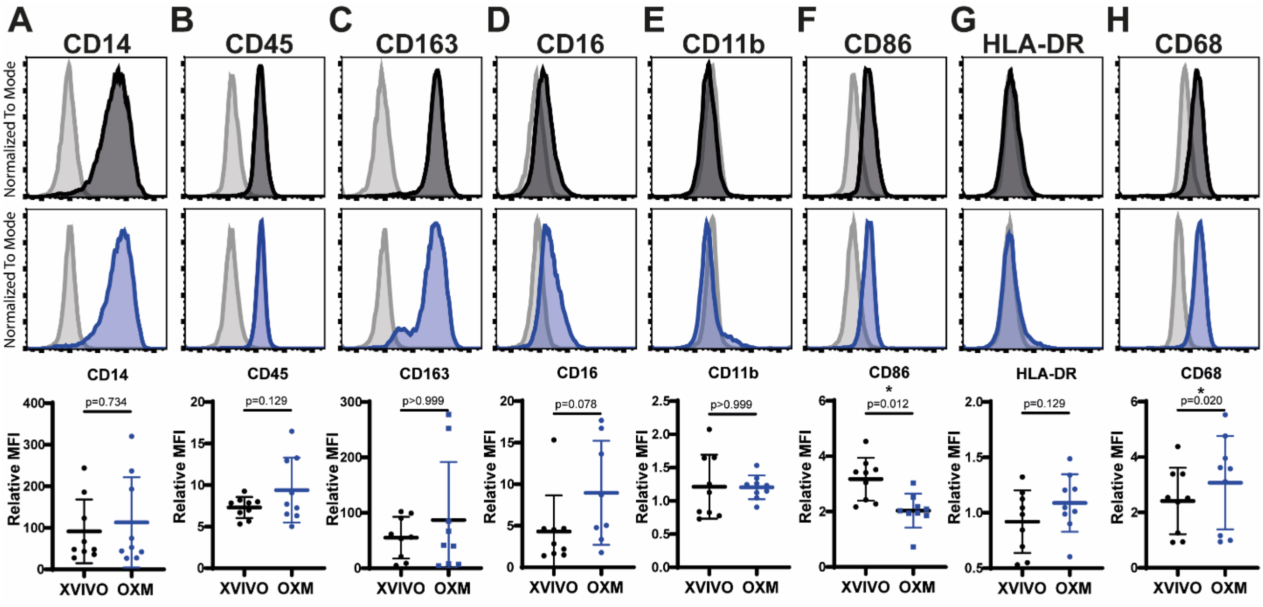

Figure S1

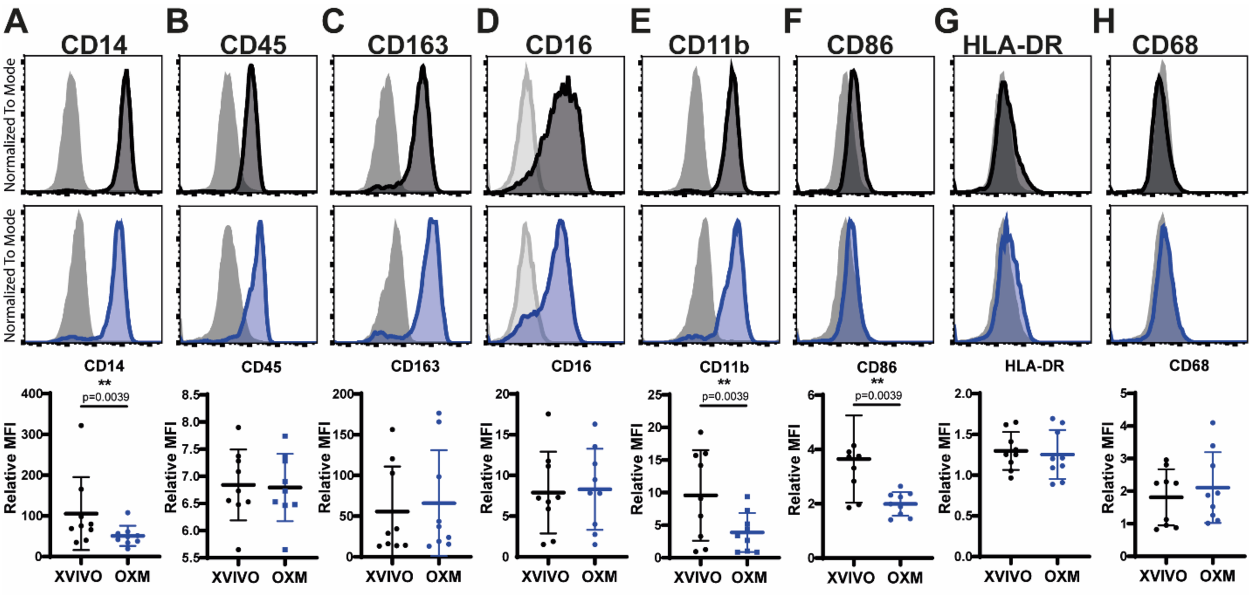

A CD14
B CD45
C CD163
D CD16
E CD11b
F CD86
G HLA-DR
H CD68
Normalized To Mode
Normalized To Mode
CD14
**
p=0.0039
Relative MFI
XVIVO OXM
CD45
CD163
CD16
CD11b
**
p=0.0039
CD86
**
p=0.0039
HLA-DR
CD68

Figure S2

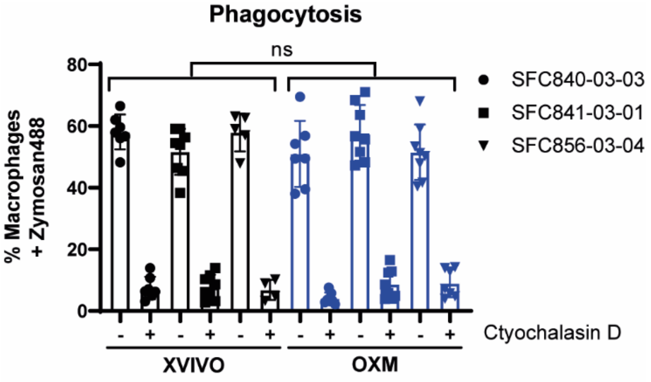
Phagocytosis
ns
80
60
40
20
0
% Macrophages + Zymosan488
SFC840-03-03
SFC841-03-01
SFC856-03-04
- + - + - + - + - + - +
Ctyochalasin D
XVIVO
OXM

Figure S3

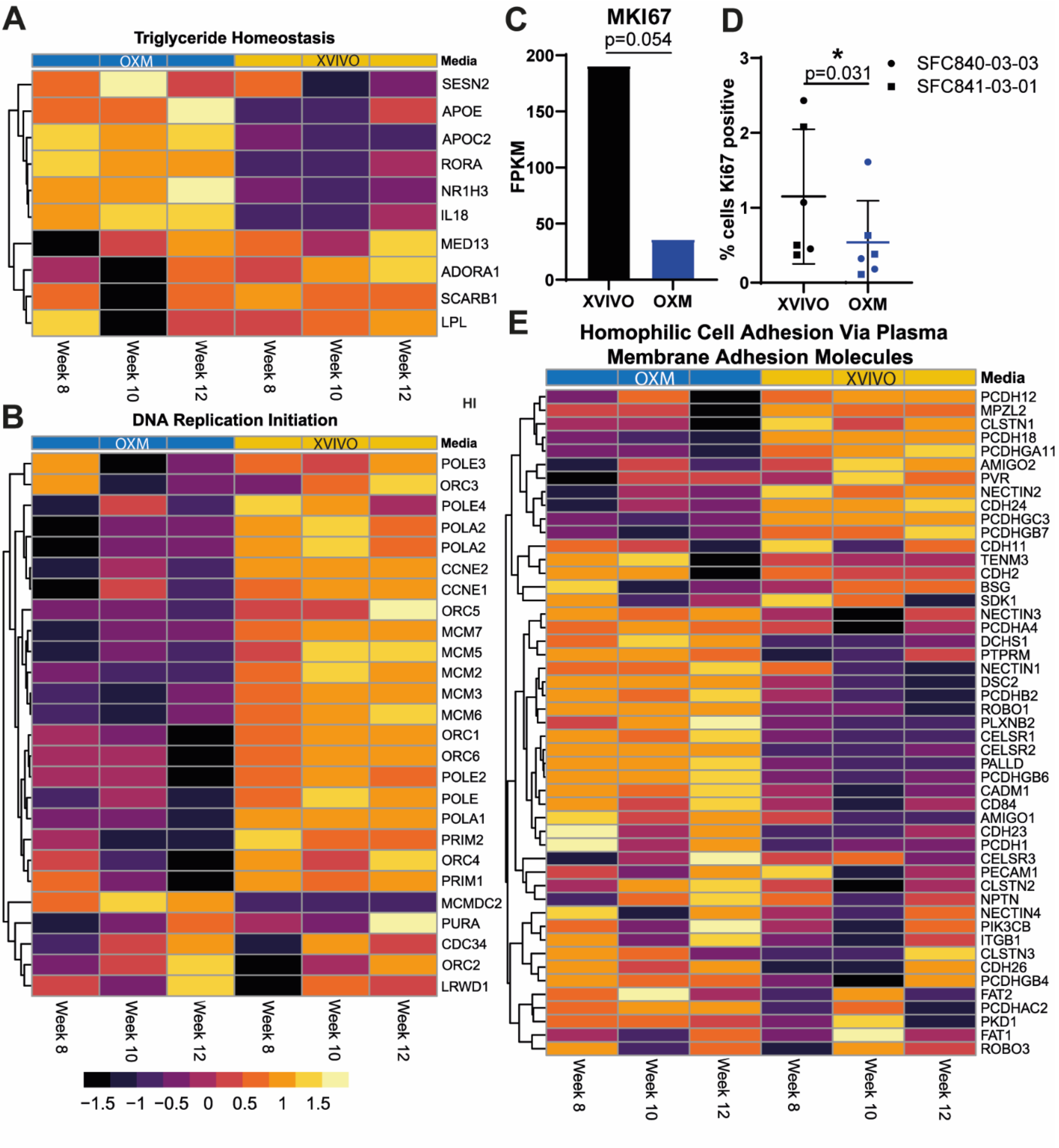
A
Triglyceride Homeostasis
OXM
XVIVO
Media
SESN2
APOE
APOC2
RORA
NR1H3
IL18
MED13
ADORA1
SCARB1
LPL
Week 8
Week 10
Week 12
Week 8
Week 10
Week 12
HI
B
DNA Replication Initiation
OXM
XVIVO
Media
POLE3
ORC3
POLE4
POLA2
POLA2
CCNE2
CCNE1
ORC5
MCM7
MCM5
MCM2
MCM3
MCM6
ORC1
ORC6
POLE2
POLE
POLA1
PRIM2
ORC4
PRIM1
MCMDC2
PURA
CDC34
ORC2
LRWD1
Week 8
Week 10
Week 12
Week 8
Week 10
Week 12
−1.5 −1 −0.5 0 0.5 1 1.5
C
MKI67
p=0.054
FPKM
200
150
100
50
0
XVIVO
OXM
D
*
p=0.031
% cells Ki67 positive
3
2
1
0
XVIVO
OXM
SFC840-03-03
SFC841-03-01
E
Homophilic Cell Adhesion Via Plasma Membrane Adhesion Molecules
OXM
XVIVO
Media
PCDH12
MPZL2
CLSTN1
PCDH18
PCDHGA11
AMIGO2
PVR
NECTIN2
CDH24
PCDHGC3
PCDHGB7
CDH11
TENM3
CDH2
BSG
SDK1
NECTIN3
PCDHA4
DCHS1
PTPRM
NECTIN1
DSC2
PCDHB2
ROBO1
PLXNB2
CELSR1
CELSR2
PALLD
PCDHGB6
CADM1
CD84
AMIGO1
CDH23
PCDH1
CELSR3
PECAM1
CLSTN2
NPTN
NECTIN4
PIK3CB
ITGB1
CLSTN3
CDH26
PCDHGB4
FAT2
PCDHAC2
PKD1
FAT1
ROBO3
Week 8
Week 10
Week 12
Week 8
Week 10
Week 12

Figure S4

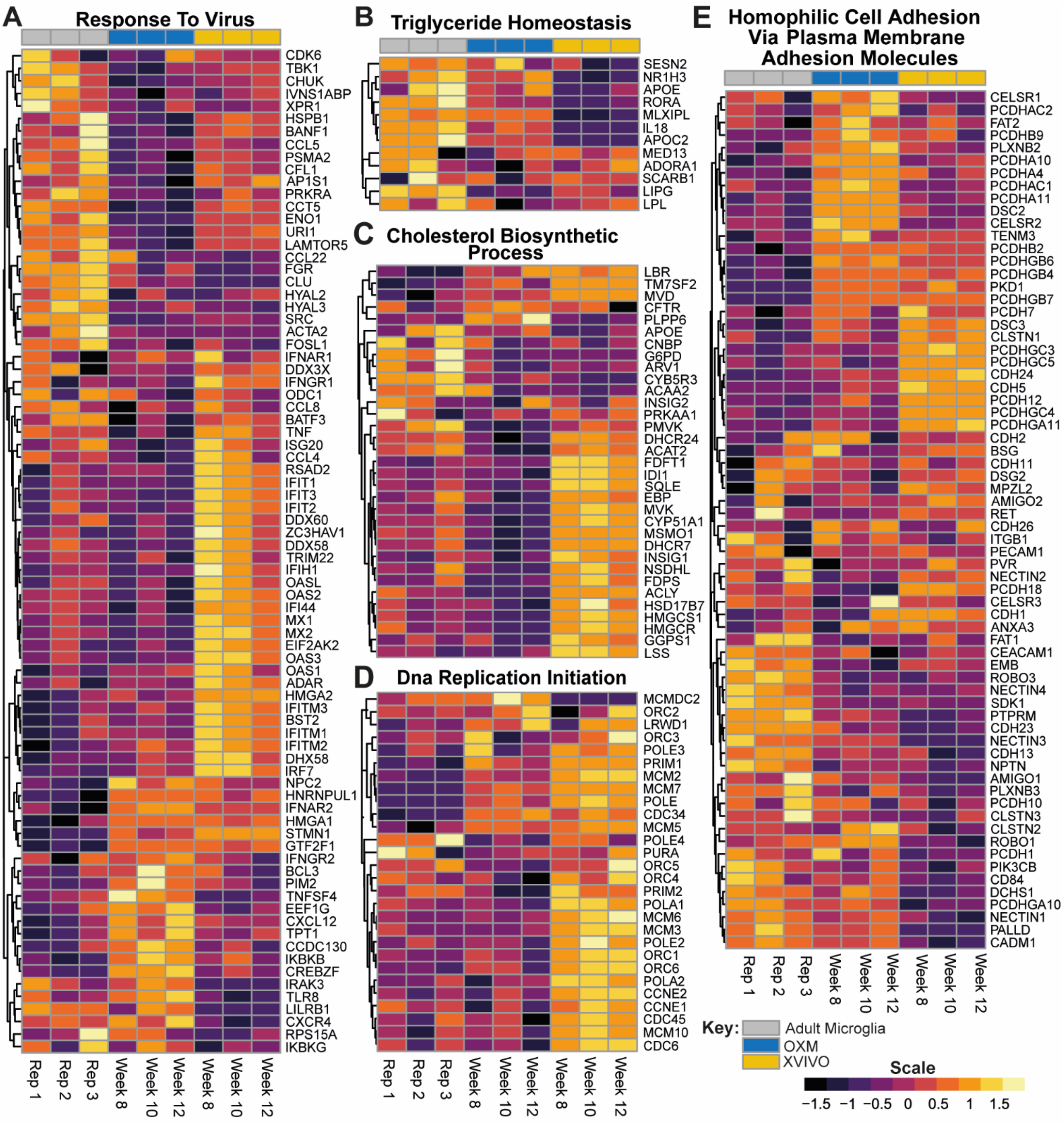

A Response To Virus
B Triglyceride Homeostasis
C Cholesterol Biosynthetic Process
D Dna Replication Initiation
E Homophilic Cell Adhesion Via Plasma Membrane Adhesion Molecules
Rep 1
Rep 2
Rep 3
Week 8
Week 10
Week 12
Key:
Adult Microglia
OXM
XVIVO
Scale
−1.5 −1 −0.5 0 0.5 1 1.5

Figure S5

Figure Legends

Figure S1: Macrophage total marker phenotype. Related to Figure 3.

Total expression of A) CD14, B) CD45, C) CD163, D) CD-16, E) CD11b, F) CD86, G) HLA-DR, and H) CD68 as measured by flow cytometry in macrophages. Histograms show fluorescence intensity (x-axis) normalised to the mode (y-axis) for macrophages cultured in XVIVO (black) or OXM (Blue), relative to the isotype control (grey). Dot plots show ratio of the geometric MFI compared to the isotype control. Bars display mean ± SD. n=12 across 3 independent cell lines. Significance was calculated by Wilcoxon matched-pairs signed rank t-test. Significance is shown when $p<0.05$ (*), <0.01 (**), <0.001 (***)

Figure S2: PreMac surface marker phenotype. Related to Figure 3.

Surface expression of A) CD14, B) CD45, C) CD163, D) CD-16, E) CD11b, F) CD86, G) HLA-DR, and H) CD68 as measured by flow cytometry on PreMac cells. Histograms show fluorescence intensity (x-axis) normalised to the mode (y-axis) for macrophages cultured in XVIVO (black) or OXM (Blue), relative to the isotype control (grey). Dot plots show ratio of the geometric MFI compared to the isotype control. Bars display mean ± SD. n=12 across 3 independent cell lines. Significance was calculated by Wilcoxon matched-pairs signed rank t-test. Significance is shown when $p<0.05$ (*), <0.01 (**), <0.001 (***)

Figure S3: Phagocytosis in different culture conditions. Related to Figure 3.

Number of macrophages that are positive for Alexa-488 conjugated zymosan coated beads after 30-minute incubation, as measured by flow cytometry. Control cells were pre-treated with 10µM Cytochalasin D for 1 hour to inhibit phagocytosis. n=8 across 3 independent cell lines. Significance was calculated by 2-way ANOVA, Sidak's multiple comparison test. Significance is shown when $p<0.05$ (*), <0.01 (**), <0.001 (***)

Figure S4: Macrophage cell cycle state. Related to Figure 4.

A-B, E) Heatmaps of GO terms for; A) Triglyceride Homeostasis, B) DNA Replication Initiation, and E) Homophilic Cell Adhesion Via Plasma Membrane Adhesion Molecules. Results across 3 repeat measurements are shown. XVIVO in yellow, OXM in blue. The colour in each row represents the z-score of the $\log_2$ fold difference from the mean TPM value for that gene. C) Mean TPM values for *MKI67* (KI67) expression in XVIVO vs OXM-cultured cells. D) Percentage of KI67 positive cells measured by immune-staining and flow cytometry. XVIVO in black, OXM in blue. n=6 in 2 independent cell lines. Mean and standard deviation shown. Significance was calculated by Wilcoxon matched-pairs signed rank t-test. Significance is shown when $p<0.05$ (*), <0.01 (**), <0.001 (***), <0.0001 (****)

Figure S5: OXM and XVIVO cultured macrophage GO terms compared to adult microglia. Related to Figure 4.

A-E) Heat maps from figures 4 and S4 here comparing gene expression data from Abud et al. 2017 Adult Microglia (grey - left), OXM (blue - middle) and XVIVO (yellow - right) for the GO terms; A) Response to Virus, B) Triglyceride Homeostasis, C) Cholesterol Biosynthetic Process, D) DNA Replication Initiation, and E) Homophilic Cell Adhesion Via Plasma Membrane Adhesion Molecules. The colour in each row represents the z-score of the $\log_2$ fold difference from the mean TPM value for that gene.

Reference	Species	Stem Cell	Co-culture, EB, or monolayer?	# steps	Duration	Defined?	# supplemented growth factors	Serum?	Proprietary component?	Approx. Yield	Notes
Wiles and Keller, 1991	Mouse	ESC	EB	2	2-8 weeks	No	IL-3, M-CSF	10-15%	No	N/A	Primarily a haematopoiesis protocol with details on driving Macrophage differentiation
Nakano et al., 1994	Mouse	ESC	Co-culture with OP9 cells	2	10 days	No	M-CSF	20%	No	700/ stem cell	Primarily a haematopoiesis protocol with details on driving Macrophage differentiation
Moore et al., 1998	Mouse	ESC	EB	3	15-17 days	No	IL-3, M-CSF	10-15%	No	1-4/stem cell	media based on Wiles and Keller 1991
Lindmark et al., 2004	Mouse	ESC	EB	3	15-17 days	No	IL-3, M-CSF, IL-1B	10%	Yes	N/A	Based on Moore et al. 1998
Anderson et al., 2006	Human	ESC	Co-culture with S17 cells	3	30-37 days	No	GM-CSF, M-CSF	10-15%	No	N/A	
Odegaard et al., 2007	Mouse	ESC	EB	5	Infinite	No	SCF, B-estradiol, IL-3	10-15%	Yes	Infinite	Myeloid progenitors immortalised using retrovirus expressing Hox9a
Karlsson et al., 2008	Human	ESC	EB	3	2 weeks - 4 months	No	IL-3, M-CSF	10%	No	3-5x10^6/EB	Macrophages produced long term
Senju et al., 2009	Mouse	iPSC	Co-culture with OP9 cells	3	22-25 days	No	GM-CSF, M-CSF	20% & 5%	No	400-600/ stem cell	Yield for dendritic cells, not macrophages
Choi et al., 2009	Human	ESC and iPSC	Co-culture with OP9 cells	3	15-24 days	No	GM-CSF, M-CSF, IL-1B	10%	No	15 per stem cell	
Subramanian et al., 2011	Human	ESC	EB	4	21-22 days	No	SCF, IL-3, IL-6, M-CSF, BMP-4, FLT3L, G-CSF, GM-CSF, M-CSF	20%	No	0.45/ CD34+ progenitor	
Choi et al., 2011	Human	ESC and iPSC	Co-culture with OP9 cells	3	16-19 days	No	GM-CSF, M-CSF, IL-1B	10%	No	1 per stem cell	optimized protocol of Choi et al. 2009
Senju et al., 2011	Human	iPSC	Co-culture with OP9 cells	3	31-44 days	No	GM-CSF, M-CSF	10-20%	No	N/A	
	Human	iPSC	Monolayer	3	>36 days	Yes	GM-CSF, M-CSF	No	Yes	N/A	
Salvagiotto et al., 2011	Human	ESC and iPSC	Monolayer	5	18-22 days	Yes	BMP4, FGF, TPO, SCF, FLT3L, IL-6, IL-3, GM-CSF, M-CSF, IL-1B	No	Yes	3 per CD34+ progenitor	
Klimchenko et al., 2011	Human	ESC	EB	3	15-40 days	No	BMP4, VEGF, SCF, FLT3L, TPO, IL-3, GM-CSF, M-CSF	15%	No	300,000	
Kambal et al., 2011	Human	iPSC	Co-culture with OP9 cells	3	>9 days	No	GM-CSF, M-CSF	10%	Yes	N/A	method based on Vodyanik et al., 2005
Zhuang et al., 2012	Mouse	ESC	EB	3	10-30 days	No	IL-3, M-CSF	10%	No	20-40/stem cell	

Author	Species	Stem Cell	Co-culture, EB, or monolayer?	# steps	Duration	Defined?	# supplemented growth factors	Serum?	Proprietary component?	Approx. Yield	Notes
Yanagimachi et al., 2013	Human	iPSC	Monolayer	5	22-38 days	No	BMP4, VEGF, SCF, FGF, IL-3, TPO, FLT3L, GM-CSF, M-CSF	10%	Yes	6x10^6/30 stem cell colonies	
van Wilgenburg et al., 2013	Human	iPSC	EB	3	2 weeks - 12 months	Yes	VEGF, BMP4, SCF, FGF, IL-3, M-CSF	No	Yes	50/ stem cell	Based on Karlsson et al. 2008 method.
Brault et al., 2014	Human	iPSC	Co-culture with OP9 cells	4	25-31 days	No	GM-CSF, M-CSF	10%	No	3/ stem cell	
Schwartz et al., 2015	Human	iPSC	Monolayer	6	17-25 days	No*	BMP4, Activin A, FGF, VEGF, TPO, SCF, GM-CSF, M-CSF, IL-1B	10%	No	N/A	*Haematopoietic progenitor differentiation in fully defined medium from Uenishi et al., 2014
Lachmann et al., 2015	Human	iPSC	EB	3	17 days - 5 months	No	FGF, IL-3, M-CSF	10%	No	1x10^6/week	
Zhang et al., 2015	Human	iPSC	EB	5	22 days	No	BMP4, VEGF, SCF, TPO, FLT3L, FGF, M-CSF	20%	Yes	2x10^6/6-well plate	
Mucci et al., 2016	Mouse	iPSC	EB	3	18-28 days	No	IL-3, SCF	10-15%	No	10/ stem cell	similar protocol to Lachmann et al. 2015
Takata et al., 2017	Mouse	iPSC	Monolayer	4	11-13 days	Yes	FGF, BMP4, Activin A, VEGF, SCF, IL-3, M-CSF	No	Yes	N/A	
	Human	iPSC	Monolayer	6	23 days	Yes	BMP4, VEGF, CHIR99021*, FGF, SCF, DKK1, IL-3, IL-6, M-CSF	No	Yes	10-20/stem cell	*CHIR99021 is a WNT agonist
Hansen et al., 2018	Human	iPSC	Monolayer	5	27-28 days	Yes	VEGF, BMP4, IL-3, IL-6, TPO, SCF, FLT-3, G-CSF, GM-CSF	No	Yes	2000/ stem cell	to make myeloid cells - not just macrophages
Ackermann et al., 2018	Human	iPSC	EB	4	10 days - 7 weeks	No	IL-3, M-CSF	10%	Yes	1-3x10^7/week	Similar method to Lachmann et al 2015
Mukherjee et al., 2018	Human	iPSC	EB	3	4 weeks – 8 months	No	IL-3, M-CSF	10%	Yes	N/A	Based on van Wilgenburg et al. 2013
Cao et al., 2019	Human	iPSC	Monolayer	5	19 days	Yes	BMP4, Activin A, CHIR99021*, VEGF, FGF, SCF, SB431542**, IL-6, TPO, IL-3, M-CSF	No	No	30-40/ stem cell	Media based on Uenishi et al. 2014. * CHIR99021 - WNT agonist. †SB431542 - TGFb inhibitor
Monkley et al., 2020	Human	iPSC	EB	5	13-39 days	No	BMP4, VEGF, SCF, FGF, IL-3, TPO, FLT3L, GM-CSF, M-CSF	10%	Yes	~40/stem cell	Based on Yanagimachi et al 2013
Vaughan-Jackson et al.	*Human*	*iPSC*	*EB*	*3*	*2 weeks - 4 months*	*Yes*	VEGF, BMP4, SCF, FGF, IL-3, M-CSF	*No*	*No*	*20-30/ stem cell*	*Method based on van Wilgenburg et al. 2013*

Table S1: Methodologies for differentiation of stem cells to macrophages. Related to the Introduction.

A list of main publications describing novel methods for differentiation of macrophages from stem cells. Not including specialised macrophage subsets e.g. microglia. Reviews including these can be found elsewhere (Lee et al., 2018; Rajab et al., 2018).

Table S2: iPSC and macrophage culture media. Related to Figure 1 and 2.

Media	Component	Final Concentration	Supplier	Catalogue number
**OXE8**	Advanced DMEM/F-12	97.4%	Gibco	12634010
	GlutaMAX (100x)	2mM	Gibco	35050-038
	Heparin solution 0.2%	100ng/mL	STEMCELL™	07980
	Ascorbic Acid-2-phosphate magnesium salt	0.22mM	Sigma Aldrich	A8960
	HEPES 1M pH 7.4	15mM	Gibco	15630080
	FGF-2 basic 145aa	100ng/mL	Bio-Techne (R&D Systems)	4114-TC-01M
	TGF-beta	2ng/mL	Peprotech	AF-100-21C
**XVIVO differentiation medium**	X-VIVO 15	97.8%	SLS (Lonza)	BE02-060F
	GlutaMAX (100x)	2mM	Gibco	35050-038
	2-Mercaptoethanol (1000x)	50uM	Gibco	31350-010
	IL-3	25ng/mL	Invitrogen	PHC0033
	M-CSF	50ng/mL	Invitrogen	PHC9501
	Penicillin-Streptomycin (P/S 100x)	1%	Gibco	15140-122
**XVIVO Macrophage medium**	X-VIVO 15	97.9%	SLS (Lonza)	BE02-060F
	GlutaMAX (100x)	2mM	Gibco	35050-038
	M-CSF	50ng/mL	Invitrogen	PHC9501
	Penicillin-Streptomycin (P/S 100x)	1%	Gibco	15140-122
**OXM differentiation medium**	Advanced DMEM/F-12	96.3%	Gibco	12634010
	GlutaMAX (100x)	2mM	Gibco	35050-038
	HEPES 1M pH 7.4	15mM	Gibco	15630080
	Human recombinant Insulin solution	5µg/mL	Sigma	I9278-5ML
	Tropolone	15µM	Sigma	T89702-1G
	IL-3	25ng/mL	Invitrogen	PHC0033
	M-CSF	50ng/mL	Invitrogen	PHC9501
	Penicillin-Streptomycin (P/S 100x)	1%	Gibco	15140-122
**OXM Macrophage medium**	Advanced DMEM/F-12	96.5%	Gibco	12634010
	GlutaMAX (100x)	2mM	Gibco	35050-038
	HEPES 1M pH 7.4	15mM	Gibco	15630080
	M-CSF	50ng/mL	Invitrogen	PHC9501
	Penicillin-Streptomycin (P/S 100x)	1%	Gibco	15140-122

Complete composition of iPSC and macrophage differentiation medium including supplier and catalogue number. Advanced DMEM/F-12 formulation can be found at the suppliers site at https://www.thermofisher.com/uk/en/home/technical-resources/media-formulation.227.html (accessed 11/10/2019

Table S3: List of metabolites present in both media. Related to Figure 1.

Most likely molecule	OXM		XVIVO			measured	theoretical
	Intensity	concentration (mol/L)	Intensity	calculated concentration (mol/L)	Ion	m/z	m/z
**Tropolone**	8.10E+05	1.50E-05	1.00E+06	1.85E-05	[M-H]	121.0293	121.0295
**Pyruvic acid**	8.00E+08	1.00E-03	6.70E+06	8.38E-06	[M-H]	87.009	87.0087
**Leucine/isoleucine**	3.00E+06	4.50E-04	nd	0.00E+00	[M-H]	130.0524	130.0509
**L-Aspartic acid**	4.70E+07	5.00E-05	nd	0.00E+00	[M-H]	132.0301	132.0302
**Glutamic acid**	1.80E+09	5.00E-05	2.00E+07	5.56E-07	[M-H]	146.0458	146.0458
**Thymidine**	7.00E+05	1.50E-06	1.40E+07	3.00E-05	[M-H]	241.083	241.083
**Phenylalanine**	1.80E+05	2.15E-04	3.00E+04	3.58E-05	[M-H]	164.0716	164.0717
**C6 sugars (1)**	2.60E+08	---	5.40E+08	---	[M-H]	179.0559	179.05611
**C6 sugars (2)**	4.90E+09	---	1.00E+07	---	[M-2H]	89.0243	89.024
**Tryptophan**	1.10E+05	4.40E+05	1.60E+07	6.40E+07	[M-H]	203.0827	203.0826

Note: the C6 sugars (1) and (2) are different because the intensity ratio (C6 (1)/C6 (2)) is different between OXM and XVIVO.

Supplemental Experimental Procedures

iPSC lines

The derivation and characterisation of the iPSC lines used in this study is described elsewhere: SFC840-03-03 (Fernandes et al., 2016), SFC841-03-01 (Dafinca et al., 2016) SFC856-03-04 (Haenseler et al., 2017)). All lines were derived from dermal fibroblasts from disease-free donors recruited through StemBANCC (Morrison et al., 2015) and the Oxford Parkinson's Disease Centre: participants were recruited to this study having given signed informed consent, which included derivation of hiPSC lines from skin biopsies (Ethics Committee: National Health Service, Health Research Authority, NRES Committee South Central, Berkshire, UK, who specifically approved this part of the study (REC 10/H0505/71)). The iPSC lines were all derived using non-integrating Sendai reprogramming vectors (Cytotune, Life Technologies), cultured in mTeSR™1 (StemCell Technologies, #85850) on hESC-qualified Matrigel-coated plates (BD, #356234), passaging as clumps using 0.5 mM EDTA in PBS (Beers et al., 2012). Large-scale, low-passage frozen SNP-QCed batches were used for experiments to ensure consistency.

Cell culture reagent preparation: OXE8

Ascorbic acid was dissolved in PBS to make a 0.22 M stock and aliquoted at 1mL for storage at -20ºC indefinitely, or 4ºC for up to 3 months. FGF-2 was reconstituted according to manufacturer's instructions and a 1 mg/mL stock made in 0.1% HSA (improves stability of storage) and aliquoted at 50 µL for storage at -80ºC. 10 µg Lyophilised TGF-β was first centrifuged to pellet the powder before reconstitution in 100 µL ultra-pure sterile water. This was then transferred into 4.8 mL 0.1% HSA solution to make a 2 µg/mL stock and aliquoted at 0.5 mL for storage at -80ºC. Both FGF-2 and TGF-β once thawed are considered stable for 2 weeks at 4ºC. Avoid freeze/thaw cycles. To make OXE8 was as follows: 500 mL aDMEM/F-12, 5 mL GlutaMAX, 25 µL Heparin, 0.5 mL Ascorbic acid, 7.5 mL HEPES pH 7.4, 50 µL FGF-2, and 0.5 mL TGF-β.

Cell culture reagent preparation: OXM

The desired mass of powdered Tropolone was dissolved in ultra-pure water and filter sterilised by passing through a 0.22 µm filter. It was aliquoted at 1.5 mL and stored at -20ºC indefinitely, or 4ºC for up to 3 months. IL-3 was reconstituted according to manufacturer's instructions in ultra-pure sterile water and aliquoted at 0.5 mL for storage at -80ºC. Once thawed, it is considered stable for 1 month at 4ºC. M-CSF was reconstituted as needed according to manufacturer's instructions in 20 mM Tris-HCL pH 8.0. Each vial was resuspended in 1 mL and could be stored at for 1 month at 4ºC or stored at -80ºC. To make OXM differentiation media was as follows: 500 mL aDMEM/F-12, 5 mL GlutaMAX, 5 mL P/S, 0.25 mL Insulin solution, 7.5 mL HEPES pH 7.4, 0.75mL Tropolone, 0.5 mL M-CSF, and 0.25 mL IL-3. To make OXM macrophage medium Tropolone and IL-3 were excluded.

Cell culture

iPSC were cultured in mTeSR™ 1 on Geltrex™ (Gibco, #A1413201)-coated tissue culture dishes and passaged using TrypLE™ Express (Gibco, #12604013). For 24 hours after plating, media was supplemented with 10 µM Y-27632 (Abcam, ab120129). Vero-76 and HEK293T cells were cultured in DMEM made to 10% FBS (Sigma, #F9665) and 1% Pen/Strep (Gibco, #15140122). All cells were incubated at 37ºC, 5% CO_2.

Macrophage differentiation

iPSC were differentiated into macrophages using a protocol based on that previously described (van Wilgenburg et al., 2013). The updated method is as follows. Aggrewell™ 800 plates (Stemcell technologies, #34815) were prepared by addition of 0.5 mL Anti-Adherence Rinsing solution (Stemcell technologies, #07010) and centrifugation at 3000g for 3 minutes to remove bubbles from the microwells. Rinse solution was then aspirated and replaced with 1 mL of 2X concentrated EB medium (1X EB media: OXE8 media supplemented with 50 ng/mL BMP4 (Peprotech, #PHC9534), 50 ng/mL VEGF (Peprotech, #PHC9394), and 20 ng/mL SCF (Miltenyi Biotec, #130-096-695) supplemented with 10 µM Y-27632. iPSC were resuspended by washing with PBS, incubating in TrypLE Express for 3-5 minutes

at 37°C, 5% CO_2, followed by gentle lifting in Advanced-DMEM/F12 to achieve single cell suspension. Cells were counted and pelleted by centrifugation at 400g for 5 minutes. After centrifugation, cells were resuspended at $4x10^6$ cells/mL in OXE8 supplemented with 10 µM Y-27632 and 1 mL added to the Aggrewell. The Aggrewell plate was then spun at 100g for 3 minutes with no braking to encourage even distribution of cells across microwells. Cells were incubated for 4 days at 37°C, 5% CO_2 with daily feeding of 75% media change with EB medium, by aspiration of 1 mL by pipette and gentle addition of 1 mL fresh media twice to avoid disturbance to the microwells. After 4 days, EBs were lifted from the plate using a Pasteur pipette and passed over a 40 µm cell strainer to remove dead cells, before washing into tissue culture plate with differentiation media (Supplementary Table 2). EBs were divided evenly into 2 T175 flasks and topped up to 20 mL with differentiation medium. Differentiation cultures were incubated at 37°C, 5% CO_2, with weekly feeding of an additional 10 mL until macrophage precursors (PreMac) cells started to be produced. After this point PreMac cells were collected weekly and a minimum of equal volumes of media to the volume removed were replaced into the differentiation cultures. Each harvest involved a 25-50% media change. Harvested cells were either used directly or plated in appropriately sized tissue culture plates for further culturing in XVIVO or OXM Macrophage medium (Supplementary Table 2) for a further 7 days, with a 50% media change on day 4.

Cell count, size, and viability measurements

Day 7 macrophages were resuspended by replacing media with StemPro™ Accutase™ Cell Dissociation Reagent (Stemcell technologies, #A1110501) and incubating 3-5 minutes at 37°C, 5% CO_2. Cells lifted in Accutase were diluted in macrophage media 1:10 to maintain accurate viability throughout measurements. Cell count, size, and viability were measured using the Nucleocounter® NC-3000 (Chemometec) after staining for live/dead cells using Solution-13 AO-DAPI stain (Chemometec, #910-3013) mixed 1:20 dye to cell suspension.

Adhesion assay

$5x10^4$ PreMacs were plate in triplicate in a 96-well plate in differentiation or macrophage medium for terminal differentiation. To assess adhesion, differentiated cells were incubated with StemPro™ Accutase™ for the indicated period of time before washing once with PBS followed by nuclear staining with NucBlue™ (Life Technologies, R37605) in live cell imaging solution (Life technologies, A14291DJ) for 15 minutes at room temperature. NucBlue™ solution was then reomoved and cells fixed in 2% PFA in PBS. Adherent cells were then counted by fluorescent imaging on the EVOS Fl auto and analysed on ImageJ. Percentage adherent cells was calculated by normalisation against an Accutase™ untreated and unwashed, stained and fixed only control.

Flow Cytometry

Cells were lifted using Accutase as described above as no difference in surface marker levels using this method versus 5 mM EDTA/12 mM Lidocaine has been observed (Carter et al 2009, Haenseler et al 2018). Cells in suspension were kept at 4°C or on ice throughout staining. For surface marker expression, cells were stained directly without fixation in FACS buffer (PBS supplemented to 1% FBS, 10 µg/mL human-IgG (Sigma, #I8640-100MG), and 0.01% Sodium azide). For total marker staining, cells were first fixed for 10 minutes in 2% PFA over ice. They were then permeabilised by treatment with 0.1% saponin in PBS for 1 hour at room temperature. Staining was then carried out in FACS buffer supplemented with 0.1% saponin. Isotype controls with the same fluorophores from the same company were used at the same concentration. Fluorescence was measured using the BD LSRFortessa™ X-20 (BD Biosciences) and analysed using FlowJo version 10. Antibodies used for flow cytometry of surface markers were all mouse primary conjugated antibodies. Antibodies used were as follows: CD4 (APC, clone 11830, R&D systems, #FAB3791A), CD11b (APC, clone ICRF44, Bio-legend, #301310) CD206 (APC, clone 15-2, Bio-legend, #321109), CCR5 (PE, clone 45531, R&D, #FAB182P), CD16 (PE, clone LNK16, Immunotools, #21279164), CD86 (PE, clone IT2.2, Bio-legend, 305405), CD163 (PE, clone 215927, R&D, #FAB1607P), CXCR4 (PE, clone 44717, R&D, FAB173P), CD14 (FITC, clone MEM-18, Immunotools, #21270143), CD45 (FITC, clone MEM-28, Immunotools, #21270453X2), CD68 (FITC, clone Ki-M7, BIO-RAD, #MCA2375F), CD86 (FITC, clone BU63, Immunotools, #21480863), HLA-DR (FITC, clone MEM-12, Immunotools, #21388993), IgG1κ (APC, clone MOPC-21, Bio-legend, #400120), IgG2a (APC, clone 20102, R&D, #IC003A), IgG1 (PE, clone PPV-06, Immunotools, #21275514),

IgG2bk (PE, clone MPC-11, Bio-legend, #400311), IgG1 (PE, clone 11711, R&D, #IC002P), IgG2b (PE, clone 133303, R&D, #IC0041P), IgG1 (FITC, clone PPV-06, Immunotools, #21335013), and IgG1 (FITC, BIO-RAD, #MCA928F). Staining for KI67 used a mouse primary antibody (Abcam, #ab15580) or isotype control primary IgG (Abcam, #ab172730) followed with a goat anti-mouse Alexa-488 conjugated secondary antibody (Invitrogen, #A-21131).

Cytokine and Chemokine release

As previously described (van Wilgenburg et al., 2013), 2.8x10^5 macrophages plated in a 24-well plate were stimulated with 100 ng/mL LPS (Invivogen, #tlrl-eklps) and 20 ng/mL IFNγ (Gibco, #PHC4031), or 50 ng/mL IL-4 (Gibco, #PHC0044) for 16 hours after which supernatants were collected and spun at 400g for 5 minutes to remove dead and floating cells, and stored at -80C until required. Cytokine release was measured using the Proteome Profiler Human Cytokine Array Kit (R&D systems, #ARY005B) modified to use IRDye 800CW Streptavidin (LI-COR, #926-32230) according to R&D systems protocol (https://www.rndsystems.com/resources/technical/use-proteome-profiler-arrays-li-cor-detection) for visualizing results on the LI-COR Odyssey 9260 and quantification using Image Studio Lite version 5.2. To quantify TNFα, 5x104 macrophages plated in a 96-well plates were stimulated with 100 ng/mL LPS or 50 ng/mL IL-4 in 100 µL macrophage medium for 16 hours. Supernatants were spun and stored at -80°C until quantification by ELISA according to manufacturer's instructions (Invitrogen, 88-7346-88).

Phagocytosis

Macrophages were cultured at 2x10^5 cells/well in a 24-well plate. Control cells were pre-treated for 1 hour with 10 µM Cytochalasin D (Cambridge Bioscience, #11330-1 mg-CAY). 12.5 µg Zymosan A (*S. cerevisiae*) BioParticles™ Alexa Fluor-488 (ThermoFisher, #Z23373) were added to each well of cells and incubated for 30 minutes at 37°C, 5% CO_2. Particles not taken up were quenched with 0.025% (v/v) Trypan Blue (Sigma, #T8154) in PBS before lifting the cells with Accutase as described above. Cells were fixed with 4% paraformaldehyde (PFA) (Alfa Aesar, #J61899.AK) before measuring fluorescence with the BD LSRFortessa™ X-20.

Lentivirus preparation and Infection assay

Lentiviral vectors were produced using PEI-mediated transfection of HEK 293T cells with pNL4.3R-E-eGFPT2ANef and pBa-L. Lentiviral containing supernatant was harvested at 48 hours, filtered, and concentrated 100X using PEG6000.

5x10^4 macrophages plated in 96-well format were infected with the stated volumes of virus diluted to 100 µL and incubated for 72 hours. Cells were resuspended by pipetting after using 5 mM EDTA in PBS at 4°C for 30 minutes, and fixed in 1% PFA. GFP positive cells were measured by flow cytometry on the BD LSRFortessa™ X-20.

One-step growth of Zika virus (ZIKV) in macrophages.

ZIKV isolate MR1766, Uganda 1947, was obtained from EVAg, the European Virus Archive. 2x10^5 macrophages in a 24-well dish were infected with 2 x 10^6 pfu of ZIKV. After 4 hours at 37°C, 5% CO_2, supernatants were aspirated and replaced with 700 µL fresh media, and incubation continued. 200 µL samples of supernatant were harvested at intervals from 6h to 48 hours post-infection, clarified by centrifugation and stored at -80°C.

Titration of ZIKV infectivity.

Virus-containing supernatants were serially diluted 10-fold and 100 µL added to each well of a 24-well plate. 2.5x10^5 Vero-76 Cells in maintenance media (DMEM containing 1% FBS and 1% Pen-Strep to maintain cell viability without proliferation) were then added. Cells were incubated with virus for 2 hours at 37°C, 5% CO_2, before adding a semi-solid overlay of 1.5% Carboxymethyl cellulose sodium salt, low viscosity (Sigma, #C5678) in Vero maintenance media and returned to 37°C, 5% CO_2. At 72 hours, the semi-solid overlay was removed, the cell monolayers washed with PBS, and plaques were revealed with Amido black stain.

RNA-seq sample preparation, library construction, and data generation

RNA was extracted from 1x10^6 macrophages (SFC840-03-03) differentiated from PreMac cells harvested at week 8, 10, and 12 of differentiation, using the RNeasy Mini Kit (QIAGEN, # 74104) according to manufacturer's directions, including optional DNase treatment step. Samples were sent to Novogene on dry ice for analysis. RNA degradation and contamination was monitored on 1% agarose gels. RNA purity was checked using the NanoPhotometer® spectrophotometer (IMPLEN, CA, USA). RNA integrity and quantitation were assessed using the RNA Nano 6000 Assay Kit of the Bioanalyzer 2100 system (Agilent Technologies, CA, USA). A total amount of 1 µg RNA per sample was used as input material for the RNA sample preparations. Sequencing libraries were generated using NEBNext® Ultra TM RNA Library Prep Kit for Illumina® (NEB, USA) following manufacturer's recommendations and index codes were added to attribute sequences to each sample. Briefly, mRNA was purified from total RNA using poly-T oligo-attached magnetic beads. Fragmentation was carried out using divalent cations under elevated temperature in NEBNext First Strand Synthesis Reaction Buffer (5X). First strand cDNA was synthesized using random hexamer primer and M-MuLV Reverse Transcriptase (RNase H-). Second strand cDNA synthesis was subsequently performed using DNA Polymerase I and RNase H. Remaining overhangs were converted into blunt ends via exonuclease/polymerase activities. After adenylation of 3' ends of DNA fragments, NEBNext Adaptor with hairpin loop structure were ligated to prepare for hybridization. In order to select cDNA fragments of preferentially 150~200 bp in length, the library fragments were purified with AMPure XP system (Beckman Coulter, Beverly, USA). Then 3 µl USER Enzyme (NEB, USA) was used with size-selected, adaptorligated cDNA at 37 °C for 15 min followed by 5 min at 95 °C before PCR. Then PCR was performed with Phusion High-Fidelity DNA polymerase, Universal PCR primers and Index (X) Primer. At last, PCR products were purified (AMPure XP system) and library quality was assessed on the Agilent Bioanalyzer 2100 system. The clustering of the index-coded samples was performed on a cBot Cluster Generation System using PE Cluster Kit cBot-HS (Illumina) according to the manufacturer's instructions. After cluster generation, the library preparations were sequenced on an Illumina platform and paired-end reads were generated.

RNA-Seq data analysis

Raw reads were pre-processed using fastp (v.0.20.1) software (Chen et al., 2018). In addition to the default filtering parameters, polyN sequences in read tails and adapter sequences were trimmed, and mismatched base pairs were corrected by overlap analysis. Filtered and trimmed reads were mapped to a full decoy-aware transcriptome using the Gencode version 34 reference human transcriptome and GRCh38 primary assembly genome. Salmon (v.1.3.0) selective alignment was used for transcript quantification with optional flags correcting for GC and random hexamer priming biases (Srivastava et al., 2019). All downstream analyses were done using the R (v.4.0) programming language (R Core Team 2020). The raw counts were imported, scaled to the average transcript length over samples and the library size ('LengthScaledTPM'), and summarised to the gene level using the tximport package (v.1.16.0) (Soneson et al., 2016). Lowly expressed genes with length-scaled abundance estimates less than 10 in 3 samples were filtered out. Differential expression was tested using limma-voom (Law et al., 2014). GO enrichment analysis was conducted using the EGSEA package (Alhamdoosh et al., 2017). GO terms with less than 10 genes were excluded from the enrichment analysis. The background gene set used for the analysis was the total number of the unique genes observed in the experiment. A publicly available dataset of human *ex vivo* and iPSC-derived microglia was processed as above and used as a reference (Abud et al., 2017). Principal component analysis was performed with the prcomp package to represent the level of transcriptomic similarity amongst the samples from both studies. To visualise expression of genes from a particular GO term, the length-scaled gene abundance estimates from both studies were transformed to counts per million (TMM-adjusted), Z-score standardise and presented on a heatmap using the pheatmap package.

Liquid chromatography-mass spectrometry (LC-MS) analysis of media

Samples of the media were prepared for LC-MS analysis as described before (Ebrahimi et al., 2020). Briefly, 500 µL of each media was filtered using Amicon Ultracentrifugal filters (3 or 10 kDa cut off). The flow through was used for analysis of metabolites using high-resolution negative ion LC-MS (accuracy better than 5 ppm). The setting and methods were as described previously (Alldritt et al., 2019; Ebrahimi et al., 2020). The total ion chromatograms were analysed using MesReNova software. The MS peaks were putatively assigned using and the human metabolome database (HMDB).   The concentrations

in the XVIVO were estimated based on the known concentration of different metabolites in the OXM media the ratio of the intensities for each molecule (Concentration in XVIVO = Concentration in OXM*(intensity XVIVO / intensity OXM).

Quantification of glucose concentration

A modification of the protocol for the detection of glucose using the Glucose (HK) Assay Kit (Supelco, # GAHK20-1KT) from culture media was done as follows: 100 µL of culture media were diluted 1:10 and 1:20 in dH2O. 20 µL of each dilution were added onto a well of a 96-well flat-bottom cell culture treated plate. Glucose solution was freshly made up by resuspending the vial using 20 mL of dH2O. 100 µL of glucose solution were added per well. Plates were incubated at 35ºC for 15 minutes before measuring absorbance at 340 nm using a plate reader. Values were compared to known values from a standard curve. The standard curve was done using seven serials dilutions (top 2.5 mM) of media culture with known composition (Dulbecco's Modified Eagle Medium – 25 mM). Each sample was run in duplicates on the plate.

Supplemental References

Abud, E.M., Ramirez, R.N., Martinez, E.S., Healy, L.M., Nguyen, C.H.H., Newman, S.A., Yeromin, A. V., Scarfone, V.M., Marsh, S.E., Fimbres, C., et al. (2017). iPSC-Derived Human Microglia-like Cells to Study Neurological Diseases. Neuron *94*, 278-293.e9.

Ackermann, M., Kempf, H., Hetzel, M., Hesse, C., Hashtchin, A.R., Brinkert, K., Schott, J.W., Haake, K., Kühnel, M.P., Glage, S., et al. (2018). Bioreactor-based mass production of human iPSC-derived macrophages enables immunotherapies against bacterial airway infections. Nat. Commun. *9*, 1–13.

Alhamdoosh, M., Law, C.W., Tian, L., Sheridan, J.M., Ng, M., and Ritchie, M.E. (2017). Easy and efficient ensemble gene set testing with EGSEA. F1000Research *6*, 1–38.

Alldritt, I., Whitham-Agut, B., Sipin, M., Studholme, J., Trentacoste, A., Tripp, J.A., Cappai, M.G., Ditchfield, P., Devièse, T., Hedges, R.E.M., et al. (2019). Metabolomics reveals diet-derived plant polyphenols accumulate in physiological bone. Sci. Rep. *9*, 1–12.

Anderson, J.., Bandi, S., Kaufman, D.., and Akkina, R. (2006). Derivation of normal macrophages from human embryonic stem (hES) cells for applications in HIV gene therapy. Retrovirology *3*.

Brault, J., Goutagny, E., Telugu, N., Shao, K., Baquié, M., Satre, V., Coutton, C., Grunwald, D., Brion, J.P., Barlogis, V., et al. (2014). Optimized generation of functional neutrophils and macrophages from patient-specific induced pluripotent stem cells: Ex vivo models of X0-Linked, AR220- and AR470-chronic granulomatous diseases. Biores. Open Access *3*, 311–326.

Cao, X., Yakala, G.K., van den Hil, F.E., Cochrane, A., Mummery, C.L., and Orlova, V. V. (2019). Differentiation and Functional Comparison of Monocytes and Macrophages from hiPSCs with Peripheral Blood Derivatives. Stem Cell Reports *12*, 1282–1297.

Chen, S., Zhou, Y., Chen, Y., and Gu, J. (2018). Fastp: An ultra-fast all-in-one FASTQ preprocessor. Bioinformatics *34*, i884–i890.

Choi, K., Vodyanik, M.A., and Slukvin, I.I. (2009). Generation of mature human myelomonocytic cells through expansion and differentiation of pluripotent stem cell–derived lin–CD34+CD43+CD45+ progenitors. J. Clin. Invest. *119*, 2818.

Choi, K.D., Vodyanik, M., and Slukvin, I.I. (2011). Hematopoietic differentiation and production of mature myeloid cells from human pluripotent stem cells. Nat. Protoc. *6*, 296–313.

Dafinca, R., Scaber, J., Ababneh, N., Lalic, T., Weir, G., Christian, H., Vowles, J., Douglas, A.G.L., Fletcher-Jones, A., Browne, C., et al. (2016). C9orf72 Hexanucleotide Expansions Are Associated with Altered Endoplasmic Reticulum Calcium Homeostasis and Stress Granule Formation in Induced Pluripotent Stem Cell-Derived Neurons from Patients with Amyotrophic Lateral Sclerosis and Frontotemporal Demen. Stem Cells *34*, 2063–2078.

Ebrahimi, K.H., Howie, D., Rowbotham, J.S., McCullagh, J., Armstrong, F.A., and James, W.S. (2020). Viperin, through its radical-SAM activity, depletes cellular nucleotide pools and interferes with mitochondrial metabolism to inhibit viral replication. FEBS Lett. *594*, 1624–1630.

Fernandes, H.J.R., Hartfield, E.M., Christian, H.C., Emmanoulidou, E., Zheng, Y., Booth, H., Bogetofte, H., Lang, C., Ryan, B.J., Sardi, S.P., et al. (2016). ER Stress and Autophagic Perturbations Lead to Elevated Extracellular α-Synuclein in GBA-N370S Parkinson's iPSC-Derived Dopamine Neurons. Stem Cell Reports *6*.

Haenseler, W., Sansom, S.N., Buchrieser, J., Newey, S.E., Moore, C.S., Nicholls, F.J., Chintawar, S., Schnell, C., Antel, J.P., Allen, N.D., et al. (2017). A Highly Efficient Human Pluripotent Stem Cell Microglia Model Displays a Neuronal-Co-culture-Specific Expression Profile and Inflammatory Response. Stem Cell Reports *8*, 1727–1742.

Hansen, M., Varga, E., Aarts, C., Wust, T., Kuijpers, T., von Lindern, M., and van den Akker, E. (2018). Efficient production of erythroid, megakaryocytic and myeloid cells, using single cell-derived iPSC colony differentiation. Stem Cell Res. *29*, 232–244.

Kambal, A., Mitchell, G., Cary, W., Gruenloh, W., Jung, Y., Kalomoiris, S., Nacey, C., McGee, J., Lindsey, M., Fury, B., et al. (2011). Generation of HIV-1 resistant and functional macrophages from

hematopoietic stem cell-derived induced pluripotent stem cells. Mol. Ther. *19*, 584–593.

Karlsson, K.R., Cowley, S., Martinez, F.O., Shaw, M., Minger, S.L., and James, W. (2008). Homogeneous monocytes and macrophages from human embryonic stem cells following coculture-free differentiation in M-CSF and IL-3. Exp. Hematol. *36*, 1167–1175.

Klimchenko, O., Di Stefano, A., Geoerger, B., Hamidi, S., Opolon, P., Robert, T., Routhier, M., El-Benna, J., Delezoide, A.L., Boukour, S., et al. (2011). Monocytic cells derived from human embryonic stem cells and fetal liver share common differentiation pathways and homeostatic functions. Blood *117*, 3065–3075.

Lachmann, N., Ackermann, M., Frenzel, E., Liebhaber, S., Brennig, S., Happle, C., Hoffmann, D., Klimenkova, O., Lüttge, D., Buchegger, T., et al. (2015). Large-scale hematopoietic differentiation of human induced pluripotent stem cells provides granulocytes or macrophages for cell replacement therapies. Stem Cell Reports *4*, 282–296.

Law, C.W., Chen, Y., Shi, W., and Smyth, G.K. (2014). Voom: Precision weights unlock linear model analysis tools for RNA-seq read counts. Genome Biol. *15*, 1–17.

Lee, C.Z.W., Kozaki, T., and Ginhoux, F. (2018). Studying tissue macrophages in vitro: are iPSC-derived cells the answer? Nat. Rev. Immunol. *18*, 716–725.

Lindmark, H., Rosengren, B., Hurt-Camejo, E., and Bruder, C.E.G. (2004). Gene expression profiling shows that macrophages derived from mouse embryonic stem cells is an improved in vitro model for studies of vascular disease. Exp. Cell Res. *300*, 335–344.

Monkley, S., Krishnaswamy, J.K., Goransson, M., Clausen, M., Mueller, J., Thorn, K., Hicks, R., Delaney, S., and Stjernborg, L. (2020). Optimised generation of iPSC-derived macrophages and dendritic cells that are functionally and transcriptionally similar to their primary counterparts. PLoS One 1–17.

Moore, K.J., Fabunmi, R.P., Andersson, L.P., and Freeman, M.W. (1998). In Vitro–Differentiated Embryonic Stem Cell Macrophages. Arterioscler. Thromb. Vasc. Biol. *18*, 1647–1654.

Mucci, A., Kunkiel, J., Suzuki, T., Brennig, S., Glage, S., Kühnel, M.P., Ackermann, M., Happle, C., Kuhn, A., Schambach, A., et al. (2016). Murine iPSC-Derived Macrophages as a Tool for Disease Modeling of Hereditary Pulmonary Alveolar Proteinosis due to Csf2rb Deficiency. Stem Cell Reports *7*, 292–305.

Mukherjee, C., Hale, C., and Mukhopadhyay, S. (2018). A Simple Multistep Protocol for Differentiating Human Induced Pluripotent Stem Cells into Functional Macrophages. In Macrophages: Methods and Protocols, G. Rousselet, ed. (New York, NY: Springer New York), pp. 13–28.

Nakano, T., Kodama, H., and Honjo, T. (1994). Generation of lymphohematopoietic cells from embryonic stem cells in culture. Science (80-.). *265*, 1098–1101.

Odegaard, J.I., Vats, D., Zhang, L., Ricardo-Gonzalez, R., Smith, K.L., Sykes, D.B., Kamps, M.P., and Chawla, A. (2007). Quantitative expansion of ES cell-derived myeloid progenitors capable of differentiating into macrophages. J. Leukoc. Biol. *81*, 711–719.

Rajab, N., Rutar, M., Laslett, A.L., and Wells, C.A. (2018). Designer macrophages: Pitfalls and opportunities for modelling macrophage phenotypes from pluripotent stem cells. Differentiation *104*, 42–49.

Salvagiotto, G., Burton, S., Daigh, C.A., Rajesh, D., Slukvin, I.I., and Seay, N.J. (2011). A defined, feeder-free, serum-free system to generate In Vitro hematopoietic progenitors and differentiated blood cells from hESCs and hiPSCs. PLoS One *6*.

Schwartz, M.P., Hou, Z., Propson, N.E., Zhang, J., Engstrom, C.J., Costa, V.S., Jiang, P., Nguyen, B.K., Bolin, J.M., Daly, W., et al. (2015). Human pluripotent stem cell-derived neural constructs for predicting neural toxicity. Proc. Natl. Acad. Sci. U. S. A. *112*, 12516–12521.

Senju, S., Haruta, M., Matsunaga, Y., Fukushima, S., Ikeda, T., Takahashi, K., Okita, K., Yamanaka, S., and Nishimura, Y. (2009). Characterization of dendritic cells and macrophages generated by directed differentiation from mouse induced pluripotent stem cells. Stem Cells *27*, 1021–1031.

Senju, S., Haruta, M., Matsumura, K., Matsunaga, Y., Fukushima, S., Ikeda, T., Takamatsu, K., Irie, A., and Nishimura, Y. (2011). Generation of dendritic cells and macrophages from human induced pluripotent stem cells aiming at cell therapy. Gene Ther. *18*, 874–883.

Soneson, C., Love, M.I., and Robinson, M.D. (2016). Differential analyses for RNA-seq: Transcript-level estimates improve gene-level inferences [version 2; referees: 2 approved]. F1000Research *4*, 1–22.

Srivastava, A., Malik, L., Sarkar, H., Zakeri, M., Almodaresi, F., Soneson, C., Love, M., Kingsford, C., and Patro, R. (2019). Alignment and mapping methodology influence transcript abundance estimation. BioRxiv 1–22.

Subramanian, A., Guo, B., Marsden, M.D., Galic, Z., Kitchen, S., Kacena, A., Brown, H.J., Cheng, G., and Zack, J.A. (2011). Macrophage differentiation from embryoid bodies derived from human embryonic stem cells. Hum. Mesenchymal Embryonic Stem Cells *4*, 211–232.

Takata, K., Kozaki, T., Lee, C.Z.W., Thion, M.S., Otsuka, M., Lim, S., Utami, K.H., Fidan, K., Park, D.S., Malleret, B., et al. (2017). Induced-Pluripotent-Stem-Cell-Derived Primitive Macrophages Provide a Platform for Modeling Tissue-Resident Macrophage Differentiation and Function. Immunity *47*, 183-198.e6.

Uenishi, G., Theisen, D., Lee, J.H., Kumar, A., Raymond, M., Vodyanik, M., Swanson, S., Stewart, R., Thomson, J., and Slukvin, I. (2014). Tenascin C promotes hematoendothelial development and T lymphoid commitment from human pluripotent stem cells in chemically defined conditions. Stem Cell Reports *3*, 1073–1084.

Vodyanik, M.A., Bork, J.A., Thomson, J.A., and Slukvin, I.I. (2005). Human embryonic stem cell-derived CD34+ cells: Efficient production in the coculture with OP9 stromal cells and analysis of lymphohematopoietic potential. Blood *105*, 617–626.

Wiles, M. V., and Keller, G. (1991). Multiple hematopoietic lineages develop from embryonic stem (ES) cells in culture. Development *111*, 259–267.

van Wilgenburg, B., Browne, C., Vowles, J., and Cowley, S.A. (2013). Efficient, Long Term Production of Monocyte-Derived Macrophages from Human Pluripotent Stem Cells under Partly-Defined and Fully-Defined Conditions. PLoS One *8*.

Yanagimachi, M.D., Niwa, A., Tanaka, T., Honda-Ozaki, F., Nishimoto, S., Murata, Y., Yasumi, T., Ito, J., Tomida, S., Oshima, K., et al. (2013). Robust and Highly-Efficient Differentiation of Functional Monocytic Cells from Human Pluripotent Stem Cells under Serum- and Feeder Cell-Free Conditions. PLoS One *8*, 1–9.

Zhang, H., Xue, C., Shah, R., Bermingham, K., Hinkle, C.C., Li, W., Rodrigues, A., Tabita-Martinez, J., Millar, J.S., Cuchel, M., et al. (2015). Functional Analysis and Transcriptomic Profiling of iPSC-Derived Macrophages and Their Application in Modeling Mendelian Disease. Circ. Res. *117*, 17–28.

Zhuang, L., Pound, J.D., Willems, J.J.L.P., Taylor, A.H., Forrester, L.M., and Gregory, C.D. (2012). Pure populations of murine macrophages from cultured embryonic stem cells. Application to studies of chemotaxis and apoptotic cell clearance. J. Immunol. Methods *385*, 1–14.
