# Supplementary material for: Differentiation of human induced pluripotent stem cells to authentic macrophages using a defined, serum-free, open-source medium
Source: Stem Cell Reports. 2021 Jun 24;16(7):1735–48. doi: 10.1016/j.stemcr.2021.05.018 (PMC8282471; doi:10.1016/j.stemcr.2021.05.018)
Supplement: Document S2. Article plus supplemental information [file mmc2.pdf]

Stem Cell Reports
Article

OPEN ACCESS

Differentiation of human induced pluripotent stem cells to authentic macrophages using a defined, serum-free, open-source medium

Alun Vaughan-Jackson,[1,4,*] Szymon Stodolak,[1] Kourosh H. Ebrahimi,[2] Cathy Browne,[1] Paul K. Reardon,[3] Elisabete Pires,[2] Javier Gilbert-Jaramillo,[1] Sally A. Cowley,[1] and William S. James[1,*]
[1]Sir William Dunn School of Pathology, University of Oxford, South Parks Road, Oxford OX1 3RE, UK
[2]Chemistry Research Laboratory, Department of Chemistry, University of Oxford, Oxford OX1 3TA, UK
[3]Vagelos College of Physicians and Surgeons, Columbia University, New York, NY 10032, USA
[4]Twitter: @VaughanAlun
*Correspondence: alun.v.j@gmail.com (A.V.-J.), william.james@path.ox.ac.uk (W.S.J.)
https://doi.org/10.1016/j.stemcr.2021.05.018

SUMMARY

Human induced pluripotent stem cells (iPSCs) and macrophages derived from them are increasingly popular tools for research into both infectious and degenerative diseases. However, as the field strives for greater modeling accuracy, it is becoming ever more challenging to justify the use of undefined and proprietary media for the culture of these cells. Here, we describe a defined, serum-free, open-source medium for the differentiation of iPSC-derived macrophages. This medium is equally capable of maintaining these cells compared with commercial alternatives. The macrophages differentiated in this medium display improved terminally differentiated cell characteristics, reduced basal expression of induced antiviral response genes, and improved polarization capacity. We conclude that cells cultured in this medium are an appropriate and malleable model for tissue-resident macrophages, on which future differentiation techniques can be built.

INTRODUCTION

Resident phagocytes are an evolutionarily conserved cell type in metazoans. In mammals, resident macrophages support tissue homeostasis through a wide range of specialized trophic, remodeling, and defense functions, whose importance is illustrated by their failure in malignant, degenerative, and infectious diseases (Gordon et al., 2014; Steinman and Moberg, 1994). Numerous methods for the differentiation of macrophages from stem cells have been developed over the years, building upon advances in our understanding of the requirements for hematopoiesis (Rajab et al., 2018; Rowe et al., 2016). These can be broadly categorized into three types: monolayer cultures, co-cultures with xeno cells, and via embryoid body (EB) intermediates. Each vary in number of growth factors applied, timing, complexity, yield, and cost. A summary of these methodologies can be found in Table S1. To investigate the molecular pathways involved in the pathogenesis of both infectious diseases, such as those caused by human immunodeficiency virus-1 (HIV-1) (van Wilgenburg et al., 2013) and *Mycobacterium tuberculosis* (Härtlova et al., 2018), and degenerative diseases, especially Parkinson disease (Haenseler et al., 2017a; Lee et al., 2020) and Alzheimer disease (Brownjohn et al., 2018; Garcia-Reitboeck et al., 2018), we developed a pathophysiologically authentic, yet genetically tractable, model of human tissue macrophages derived from pluripotent stem cells (Karlsson et al., 2008; van Wilgenburg et al., 2013). We have shown that this model resembles an MYB-independent early wave of myelopoiesis *in vivo* that gives rise to resident macrophages in a number of tissues (Bian et al., 2020; Buchrieser et al., 2018). Our first method involved spontaneous differentiation of mesoderm from embryonic stem cells via EBs, followed by myeloid differentiation using macrophage colony stimulating factor (M-CSF) (CSF-1) and interleukin-3 (IL-3) in a serum-supplemented medium (Karlsson et al., 2008). Since this was not effective for all pluripotent stem cell lines, and to remove the undefined serum component, we subsequently developed a serum-free method, using BMP4, VEGF, and SCF to promote mesodermal lineage and prime hemogenic endothelium differentiation during EB formation, and the serum-free X-VIVO 15 (XVIVO) medium (Lonza) during the M-CSF/IL-3-directed myeloid differentiation stage (van Wilgenburg et al., 2013).

With a greater focus than ever on ensuring research integrity in the life sciences through the adoption of open practices in data curation, publication, and the sharing of materials (Cech et al., 2003; Morey et al., 2016; National Academy of Sciences, National Academy of Engineering (US) and Institute of Medicine (US) Committee on Science, Engineering, and Public Policy, 2009), it is desirable to replace proprietary media used for culturing and differentiating human pluripotent stem cells with ones that are both defined and fully open-source. While great improvements have been made to move away from xeno-material containing co-culture and serum-based methodologies toward well-defined monolayer and EB protocols, of the methods described in Table S1, only one has been described to utilize fully defined and open-sourced media throughout differentiation (Cao et al., 2019). The

This is an open access article under the CC BY license (http://creativecommons.org/licenses/by/4.0/).

remainder either supplement cultures with serum, or rely on commercial, serum-free alternatives of undisclosed composition. The suppliers of both the commercial induced pluripotent stem cell (iPSC) culture medium, based on previously published medium (Chen et al., 2011), TeSR-E8 (STEMCELL Technologies), and X-VIVO 15 (Life Technologies) decline to publish the composition of these optimized materials on commercial grounds. Consequently, the scientific community does not know whether components that may have a material effect on macrophage physiology are present, nor their concentration. Examples could include, but are not limited to, anti-infectives, anti-inflammatories, cell-permeable iron-chelators cryoprotectants, and excessive concentrations of glucose. Accordingly, we have sought to base replacement media on widely available and open-source materials, enabling others to reproduce our experiments conveniently and without undue dependence on particular suppliers.

In this paper, we describe OXM, an open-source alternative macrophage differentiation medium based on Advanced DMEM/F-12 (aDMEM/F-12). We show that OXM generates homogeneous macrophage precursors very comparable to those produced by our earlier methods, both phenotypically and transcriptionally. We note that these cells show signatures consistent with more complete terminal differentiation, including improved morphology and cell-cycle arrest, and have lower basal expression levels of interferon-inducible genes while being more responsive to inflammatory stimulation. This method therefore generates cells that are even closer models of the “surveilling” state of homeostatic tissue macrophages.

RESULTS

XVIVO medium, but not OXM medium, contains undisclosed molecules

We first developed a serum-free, defined, open-source medium, named OXM, for differentiation of iPSCs to macrophages. Previously, we described methodologies for differentiation (Karlsson et al., 2008; van Wilgenburg et al., 2013) in which Advanced DMEM or RPMI supplemented with 10% fetal calf serum (FCS), or a serum-free alternative, XVIVO, were used to culture the cells. However, the composition of XVIVO is proprietary, we therefore could not know whether it contained additives that may affect the phenotype of cells differentiated in this medium. We therefore decided to use aDMEM/F-12 buffered with HEPES as the base of OXM. To replace FCS, additional insulin and tropolone, a cell-permeable iron chelator shown to be a suitable substitute for transferrin in cell culture (Field, R.P., Lonza Group, 2003. Animal cell culture. US Patent 6593140), were included. M-CSF and IL-3 were supplemented independently. For full composition, see Table S2.

To identify possible causes of differences between XVIVO- and OXM-cultured macrophages, we carried out a limited investigation into the chemical composition of each medium using high-resolution negative-ion liquid chromatography-mass spectrometry (LC-MS), specifically screening for metabolites and polar molecules, but not proteins or non-polar molecules. A comparison of the total ion chromatograms (TIC) shows that there are differences in the chemical composition between OXM and XVIVO (Figure 1A), as indicated by the intensity of individual peaks. Each peak was analyzed and putatively assigned as described in the supplemental experimental procedures. A list of predicted metabolites is given in the supplemental information (Table S3). Specifically, we noted a 2-fold difference in C6 sugar concentration between XVIVO and OXM. We subsequently independently confirmed this by enzymatic assay, finding 34.3 mM glucose in XVIVO and 16.7 mM in aDMEM/F-12 (Figure 1B). LC-MS also predicted that the levels of amino acids were at higher concentrations in OXM than in XVIVO except for tryptophan (44 nM in OXM, 6.3 μM in XVIVO) (Table S3). In addition, we noted that three peaks in the TIC of the XVIVO media were absent in that of the OXM media (Figure 1A). The best predicted molecules for these peaks are isolariciresinol 9′-O-alpha-L-arabinofuranoside (CID: 131751348, Figure 1C), methyl glucosinolate (CID: 9573942, Figure 1D) and dithinous acid (CID: 24490, Figure 1E). These molecules were either absent from the OXM media or below the detection limit.

Differentiation of iPSCs to macrophages in novel fully defined, open-source medium, OXM

We next sought to compare the maturation states of OXM-differentiated and cultured macrophages against those differentiated and cultured in XVIVO. EBs were formed and cultured for 4 days according to our previous publication (van Wilgenburg et al., 2013). Minor modifications to methodology were made, including culturing in an in-house, defined, open-source medium derived from E8 (Chen et al., 2011) we call OXE8. Note that, while the published composition is open-source, commercial media based on E8 have been modified and have proprietary compositions. OXE8 contains FGF-2 and transforming growth factor β and further supplements of VEGF, BMP4, and SCF (for composition see Table S2). After EB formation, as in our earlier methods, EBs were transferred into OXM- or XVIVO-based differentiation medium supplemented with IL-3 and M-CSF. For terminal macrophage differentiation media, IL-3 and tropolone were then omitted (Figure 2A; Table S2). Morphological differences between differentiation cultures were immediately apparent. EBs produced

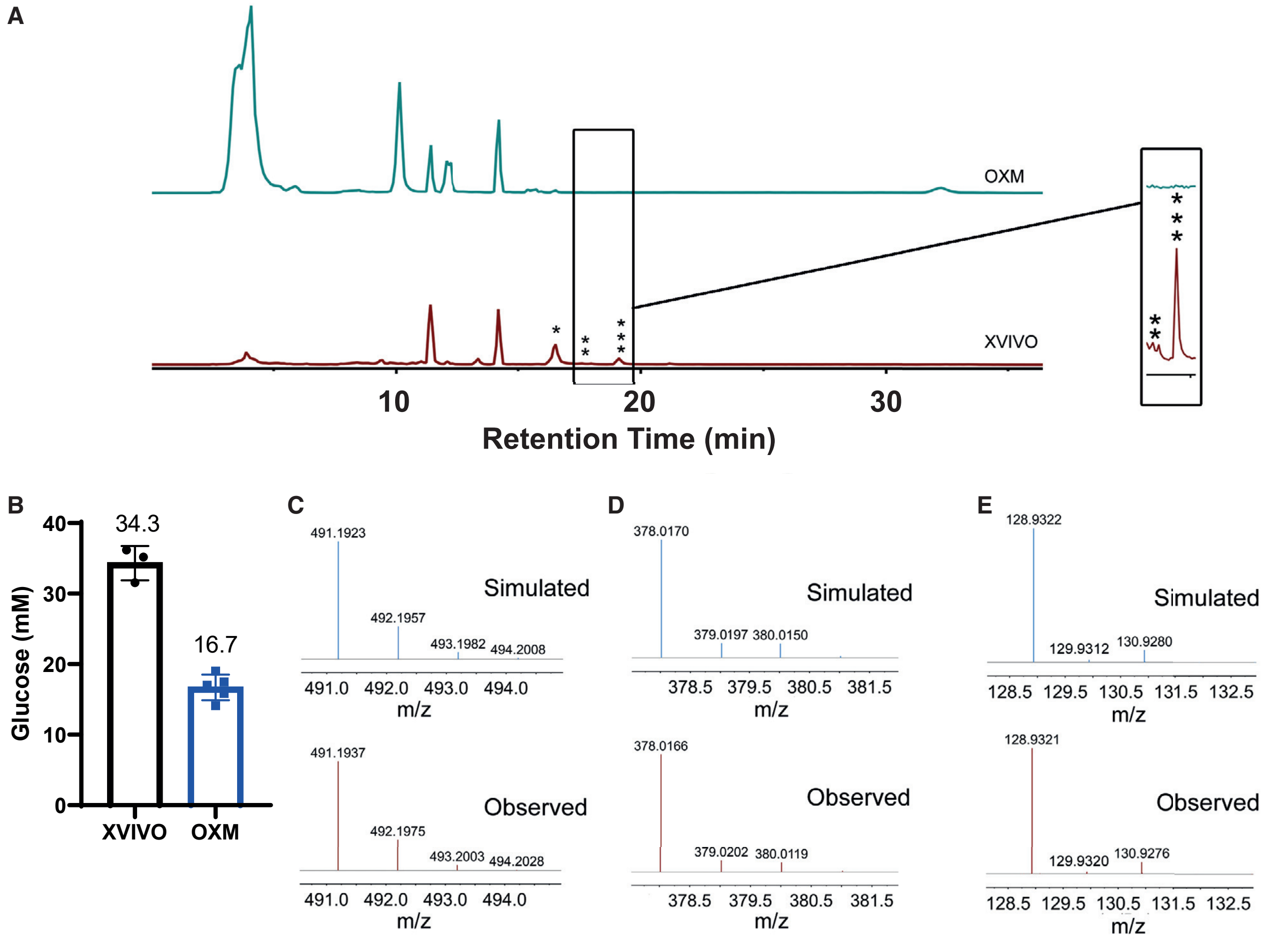

Figure 1. XVIVO medium, but not OXM medium, contains undisclosed molecules

Negative-ion high-resolution LC-MS spectroscopy was used to analyze different compounds in the media.

(A) Total ion chromatogram of the molecules in the XVIVO medium is compared with that of molecules in the OXM medium.

(B) Concentration of glucose in XVIVO and OXM media. n = 3 (XVIVO) and 5 (OXM) independent experiments. Error bars represent ±SD.

(C–E) (C) Comparison of the observed MS spectrum of the main peaks absent in OXM with the simulated spectrum of $[M\text{-}H]^-$ adduct of isolariciresinol 9′-O-alpha-L-arabinofuranoside (*), (D) that of [M + FA-H] – adduct of methyl glucosinolate (**), and (E) that of $[M\text{-}H]^-$ adduct of dithionous acid (***).

larger cyst-like structures and greater adherent stromal growth in OXM medium (Figure 2A). By week 3 of differentiation, monocyte-like macrophage precursors cells (PreMacs) had started to be released into the supernatant. This continued for >16 weeks (Figures 2B and 2C). Interestingly, OXM differentiation cultures produced a large number of PreMacs in the first 3 weeks of production before their yield reduced to 7–8 × 10^4 cells/mL/week. XVIVO differentiation cultures were slower to yield PreMacs, but by week 6 produced approximately 2-fold more cells, an average of 1.4 × 10^5 cells/mL/week (Figure 2B). After 16 weeks the average total yield was (4.48 ± 1.26) × 10^7 cells in OXM and (7.99 ± 2.61) × 10^7 cells in XVIVO (Figure 2C). PreMacs produced in OXM were significantly smaller than in XVIVO (range 15.8–19.5 μm, mean 17.9 μm, versus 13.8 to ≥20 μm, mean 19.5 μm, respectively) (Figure 2D). Note that owing to limitations of the NucleoCounter NC-3000, 20 μm is the upper limit of cell size, so XVIVO-produced cells may have been larger.

For terminal differentiation of PreMacs into macrophages, cells were cultured for a further 7 days in macrophage differentiation medium. After 7 days, XVIVO-cultured cells remain rounded or acquire a bipolar morphology, with large vesicle-like structures visible. OXM-cultured cells were morphologically heterogeneous with rounded cells, large, flattened cells, and cells with multiple projections (Figure 2A, expanded view in 2E). The size of adherent macrophages was measured after

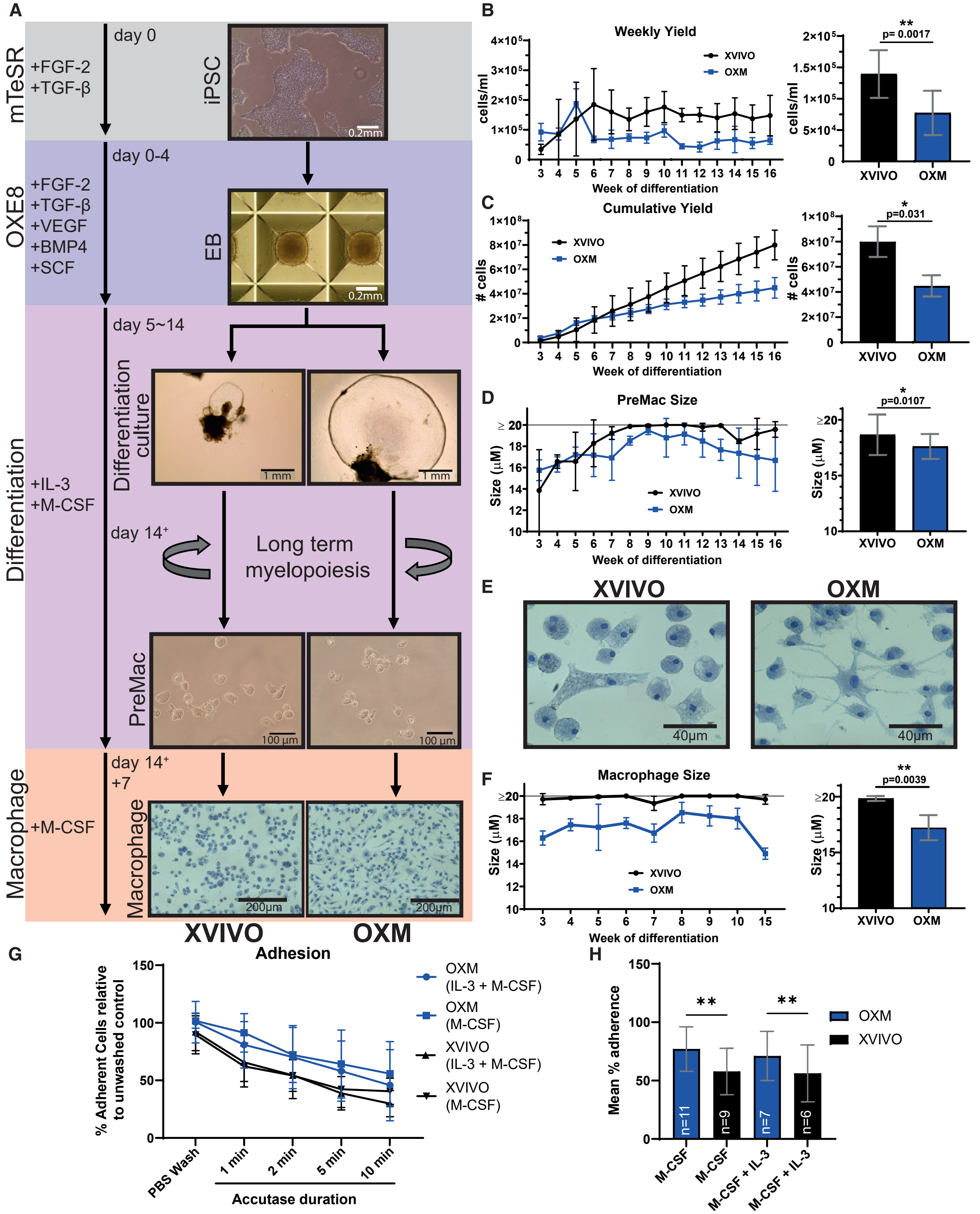

(legend on next page)

resuspension. OXM-cultured macrophages were significantly smaller than those in XVIVO: mean 17.51 μm (range 16.28–18.53 μm) versus mean 19.85 μm (range 19.35 to ≥20 μm) (Figure 2F). Differences in time taken to resuspend OXM versus XVIVO cells showed that OXM cells were more adherent, both when cultured in macrophage medium or in fresh differentiation medium (Figures 2G and 2H). In summary, OXM supports the production of more adherent, smaller macrophages than XVIVO, albeit with a lower cumulative yield.

Macrophages cultured in OXM are phenotypically similar to, but distinct from, XVIVO-cultured cells

To determine macrophage phenotype, surface marker abundance was determined by flow cytometry. Key markers of macrophage lineage, the lipopolysaccharide (LPS) co-receptor CD14 (Figure 3A), and the pan-leukocyte marker CD45 (Figure 3B), were highly expressed in macrophages derived using both media, with no significant differences. Nor was a difference observed with the expression of scavenger receptor CD163 (Figure 3C). Levels of the Fcγ immunoglobulin G (IgG) receptor, CD16, were significantly higher in OXM-cultured cells (Figure 3D). Conversely, both the β2-integrin receptor, CD11b, and the costimulatory receptor, CD86, were more highly expressed in XVIVO-cultured cells, suggesting a more M1-like phenotype (Mosser, 2003) (Figures 3E and 3F). Consistent with our previous reports (Haenseler et al., 2017b; Karlsson et al., 2008; van Wilgenburg et al., 2013), major histocompatibility complex class II (human leukocyte antigen-DR [HLA-DR]) expression is low in these unstimulated cells in either condition (Figure 3G). Expression of the tissue-resident macrophage marker CD68 was observed intracellularly but not on the cell surface, consistent with earlier literature (Gordon and Plüddemann, 2017), and was significantly higher in OXM-cultured cells (Figures 3H and S1H).

PreMac cells gave broadly similar results to those of fully differentiated macrophages, although with differences in CD14 and CD16 expression (Figure S2). Overall, the PreMacs and macrophages produced using both OXM and XVIVO media display a macrophage phenotype, but show small and consistent differences that may reflect differences in polarization.

Finally, to test the phagocytic capacity of macrophages produced under the two conditions, we measured phagocytic uptake of Alexa 488-conjugated zymosan, a yeast-derived particulate glycan. Phagocytic uptake after 30 min was not significantly different between conditions across three genetic backgrounds (Figure S3), indicating that macrophages cultured in OXM are phagocytically competent.

While transcriptionally very similar, OXM-cultured macrophages have a more homeostatic, and XVIVO-cultured macrophages a more immunologically active, signature

We used RNA sequencing (RNA-seq) to investigate the detailed expression profile of our iPSC-derived tissue macrophages. We first compared the macrophages cultured in XVIVO or OXM to a published dataset of iPSC-derived human microglia (tissue-resident macrophages of the brain) that had been differentiated via induced hematopoietic progenitor cells (iHPCs) derived using modified protocols from previous reports (Abud et al., 2017). Principal-component analysis , in which PC1 explained 27% of the variance, and PC2 explained 18%, showed tight clustering of OXM- and XVIVO-cultured cells together. The iPSC-derived macrophages showed closest similarity to microglia rather than iHPCs or monocyte-derived macrophages (MDMs), and least similarity to pluripotent cells and definitive hematopoietic lineages, such as dendritic cells (Figure 4A). This was expected because iPSC macrophages have a primitive ontogeny, similar to that of microglia,

Figure 2. Morphology throughout macrophage differentiation

(A) Schematic of iPSC-derived macrophage differentiation. Base medium is displayed on the left with main growth factor supplements adjacent.

(B) Average weekly yield (left) and overall average weekly yield (right) of PreMac cells per mL of medium removed from differentiation cultures.

(C) Cumulative yield of PreMac cells over 16 weeks of differentiation (left) and final total yield (right).

(D) PreMac size over the lifespan of the differentiation culture (left) and overall average PreMac size (right).

(E) Methylene blue (1%, w/v) staining to show macrophage morphology.

(F) Macrophage size over multiple weeks of differentiation (left) and overall average macrophage size (right).

(G) Percentage adherent cells cultured in fresh differentiation medium (M-CSF + IL-3) or macrophage medium (M-CSF) after incubation with Accutase normalized to untreated control.

(H) Average of adherent cells over time course shown in (G). (B–H) XVIVO-cultured cells represented in black versus OXM-cultured cells in blue, mean ± SD. (B–C) n = 2 experiments in each of 3 independent donors' cell lines. (D and F) n = 1 experiment in each of 3 independent donors' cell lines. (G–H) n is displayed within the bars of (H) and made up from 2 independent donors' cell lines. (B–F) Significance was calculated by Wilcoxon matched-pairs signed rank t test. (H) Significance was calculated by two-way ANOVA, Tukey's multiple comparison test. Significance is shown when *$p < 0.05$, ** $p < 0.01$, *** $p < 0.001$.

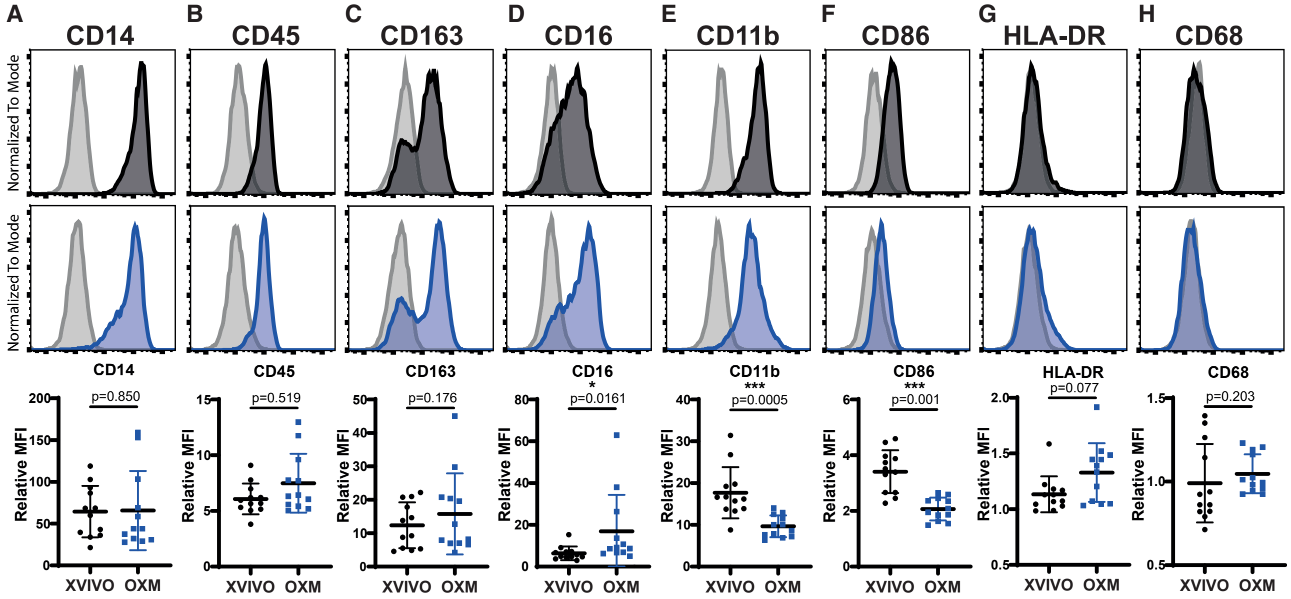

Figure 3. Macrophage surface marker phenotype

Surface expression of (A) CD14, (B) CD45, (C) CD163, (D) CD16, (E) CD11b, (F) CD86, (G) HLA-DR, and (H) CD68 as measured by flow cytometry on macrophages. Histograms show fluorescence intensity (x axis) normalized to the mode (y axis) for macrophages cultured in XVIVO (black) or OXM (blue), relative to the isotype control (gray). Dot plots show ratio of the geometric mean fluorescence intensity (MFI) compared with the isotype control. Bars display mean ± SD. n = 4 experiments in each of 3 independent donors' cell lines. Significance was calculated by Wilcoxon matched-pairs signed rank t test. Significance is shown when $^*p < 0.05$, $^{**}\ p < 0.01$, $^{***}\ p < 0.001$.

which develop from primitive, yolk sac/embryonic fetal macrophages, unlike adult definitive MDMs that develop from hematopoietic progenitors in the bone marrow (Buchrieser et al., 2017; Ginhoux and Guilliams, 2016; Vanhee et al., 2015). Further differential expression analysis found 15,951 genes shared in both OXM and XVIVO populations, and 2,335 genes unique to either OXM (1,331) or XVIVO (1,004) (Figure 4B). Analysis of the sources of this variation showed that the media used explained approximately 30% of the variation between samples, while the age of the differentiation culture (as indicated by the week of PreMac harvesting) accounted for approximately 15%, and the remainder was due to residual, undefined sources (Figure 4C).

The three genes most significantly upregulated in OXM versus XVIVO were the carbohydrate binding lectin, *CHI3L1*, the tetraspanin, *CD37*, and the protease, *HTRA4* (p = 0.0028, 0.0049, and 0.006, respectively). In XVIVO, the most upregulated genes were the interferon-inducible protein *IFIT3*, the endonuclease *RNASE1*, and the epoxidase *SQLE* (p = 0.0028, 0.006, and 0.006, respectively). The most enriched gene ontology (GO) term in XVIVO compared with OXM-cultured cells, was "response to virus" (Figures 4D and 4E). Multiple interferon-inducible genes, e.g., *MX2*, *RSAD2*, and the *IFIT* family of proteins, were more highly expressed in XVIVO-cultured cells. However, some antiviral receptors, such as *TLR8*, are more highly expressed in OXM-cultured cells. We also found enrichment of metabolism-related processes. In OXM-cultured cells, the most highly enriched GO term was "triglyceride homeostasis" (Figure S4A), and in XVIVO-cultured cells the "cholesterol biosynthetic process" was highly enriched with 23/33 genes in this term upregulated in these cells (Figures 4D and 4F). The term "DNA replication initiation" was enriched in XVIVO-cultured cells (Figure S4B). To test whether XVIVO cells may therefore be in cell cycle, we measured total KI67, which transcriptionally has 2.66-fold higher expression in XVIVO-cultured cells (Figure S4C), by immunostaining and flow cytometry. This confirmed expression is higher but note that only a small proportion of cells (XVIVO; 1.15% ± 0.90%, OXM; 0.54% ± 0.55%) are in cell cycle (Figure S4D). In OXM, the inflammation-related term "complement activation, classical pathway," cell adhesion, and several developmental terms are enriched (Figures 4E and S4E). The enrichment of the cell adhesion term supports observations that OXM-cultured cells were more adherent than XVIVO-cultured cells (Figures 2G and 2H). Overall, when compared against adult microglia, both OXM- and XVIVO-cultured cells show similar but distinct expression profiles of genes within the GO terms mentioned above (Figure S5). However, OXM macrophages, in particular, display closer levels

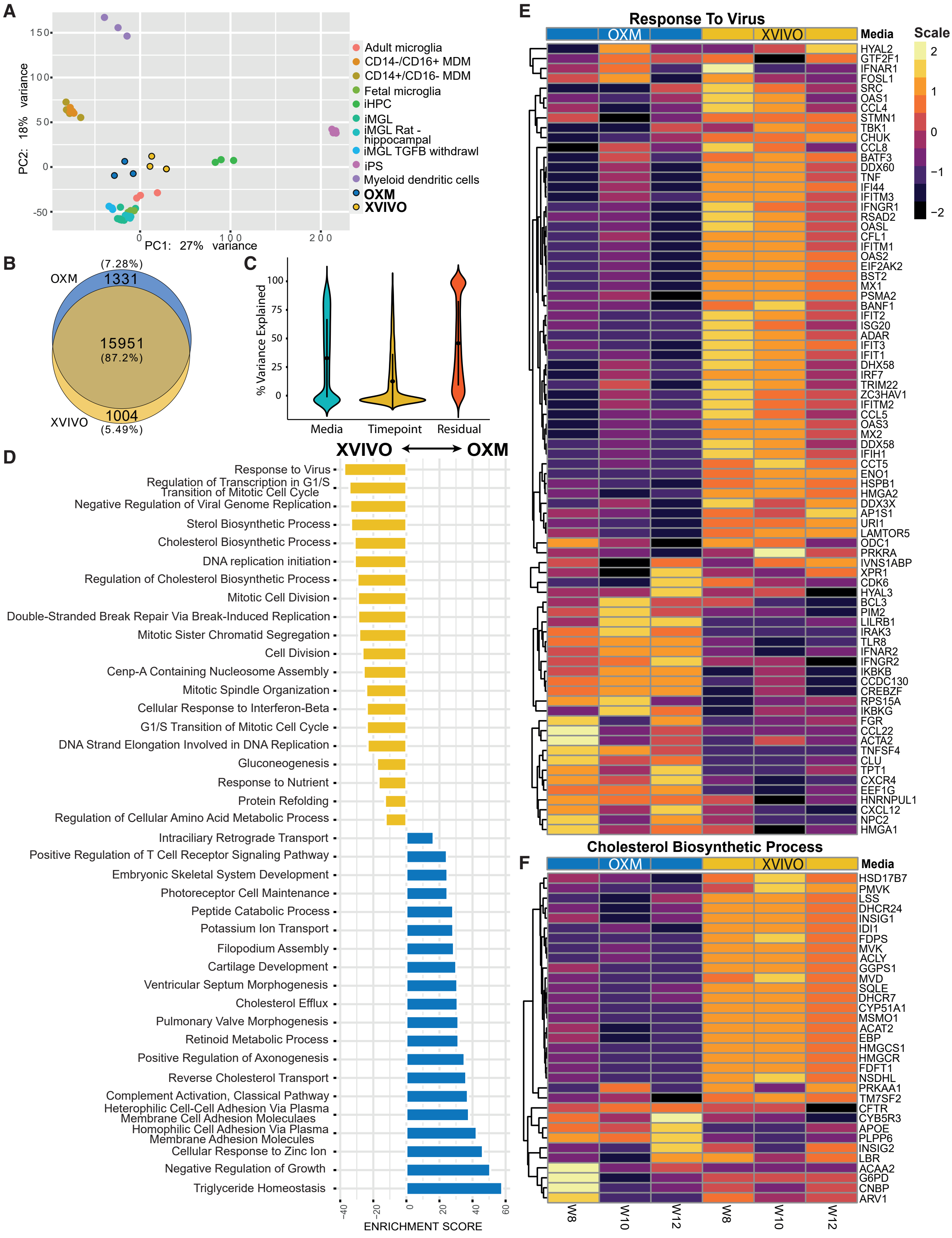

(legend on next page)

of expression of genes regulating the cell cycle and cholesterol metabolism compared with microglia (Figures S5C and S5D). Transcriptomic analysis has shown that, although cells cultured in these different media are highly similar, XVIVO-cultured macrophages display a greater tendency toward proliferation and an activated antiviral response, and OXM-cultured macrophages toward homeostasis, adhesion, and inflammation.

OXM- and XVIVO-cultured macrophages have different classical and alternative activation profiles

To test whether the subtle transcriptomic differences between the cells differentiated in the two media resulted in functional differences, we measured cytokine secretion and polarization after stimulation. Cytokine secretion was measured at resting state and after classical (LPS and INF-γ) or alternative (IL-4) activation, as described previously (Jiang et al., 2012; van Wilgenburg et al., 2013). Cytokine profiles under both conditions were consistent with the previous reports (Figure 5A). OXM-cultured cells produced noticeably more GROα, ICAM-1, IL-1ra, and IL-8 in the resting state, while XVIVO-cultured cells produced more IP-10 (CXCL10). After classical activation, XVIVO-cultured cells produced more tumor necrosis factor alpha (TNF-α), as well as low levels of IL-12p70, while OXM-cultured cells produced more RANTES (CCL5) and small quantities of IL-1β. Alternative activation suppressed secretion of resting state cytokines in both media. Consistent with the results above, LPS-induced TNF-α secretion from OXM-cultured cells quantified by enzyme-linked immunosorbent assay (ELISA) was lower than from XVIVO cells (Figure 5B).

Considering the differences in cytokine production, we next assessed macrophage surface marker polarization toward classically defined M1 and M2 states after activation. This was measured by immunostaining for CD86 and CD206, respectively (Mosser, 2003; Roszer, 2015) (Figures 5C and 5D). Although activation had no effect on CD45 expression in both conditions, CD86 expression significantly increased in OXM-cultured cells compared with XVIVO-cultured cells after classical activation (4.75- versus 0.81-fold). CD206 expression increased in both conditions after alternative activation and was not significantly different between conditions (1.86- versus 2.16-fold) (Figures 5C and 5D). In conclusion, although cells cultured under either condition can respond well to stimuli, they differ in their cytokine profile, and cells cultured in OXM display greater changes in typical macrophage polarization surface markers than those in XVIVO.

OXM-cultured macrophages are more susceptible to HIV-1 infection, but not to Zika virus

Due to their positioning as sentinel cells throughout the body, tissue-resident macrophages are often the first members of the immune system to respond to infection. Consequently, several pathogens have evolved to use these cells as a host during early infection, including viruses, such as HIV-1 and Zika virus (ZIKV). In the case of HIV-1, macrophages are hypothesized to act as a potential reservoir for latent infection due to the long-lived, non-replicating state of these cells (Honeycutt et al., 2016, 2017). To determine whether iPSC macrophages would be a suitable model for modeling this disease, macrophages were infected with a macrophage-tropic (CCR5-tropic Ba-L envelope), GFP expressing, HIV-1 pseudotype virus. Infectivity was measured by expression of GFP after 72 h by flow cytometry. A significantly higher proportion of macrophages cultured in OXM than in XVIVO were infected (6.76% ± 3.69% compared with 1.77% ± 1.23%; Figure 6A). Expression of the HIV-1 entry receptors CD4 and CXCR4 was not significantly different between conditions, and CCR5 was higher in XVIVO-cultured macrophages (mean flouresence intensity (MFI) relative to isotype: 6.16 ± 3.18 XVIVO versus 2.67 ± 1.52 OXM; Figure 6B). This difference in viral entry may be explained by the higher expression of known HIV-1 restriction factors in XVIVO-cultured cells, including the *IFITM* and the *APOBEC* families of proteins, and *TRIM5α* (Figure 4F). To test whether differences in susceptibility

Figure 4. Transcriptome of iPSC-derived macrophages

Transcriptome of macrophages as measured by RNA-seq of three biological replicates per medium condition from one genetic background (SFC840-03-03).

(A) Principal-component analysis (PCA) plot of transcriptional profile for iPSC-derived macrophages cultured in XVIVO or OXM compared with iPSC, induced hematopoietic progenitor cells (iHPCs), microglia (MGL) (adult, fetal, iPSC-derived, and iPSC-derived with TGFB withdrawal), monocytes (MDM), and dendritic cells reported previously (Abud et al., 2017).

(B) Venn diagram representing total number of genes expressed by iPSC-derived macrophages in either medium composition.

(C) Violin plot of the percentage variance attributed to each variable (medium, time point, and residual).

(D) GO term enrichment between media compositions. Top 15 most enriched results in each medium are shown. Yellow represents terms enriched in XVIVO-cultured cells, and blue in OXM-cultured cells.

(E and F) Heatmaps of GO terms for; "response to virus" and "cholesterol biosynthetic process." Results across three repeat measurements are shown. XVIVO in yellow, OXM in blue. The color in each row represents the *Z* score of the $\log_2$ fold difference from the mean TPM value for that gene.

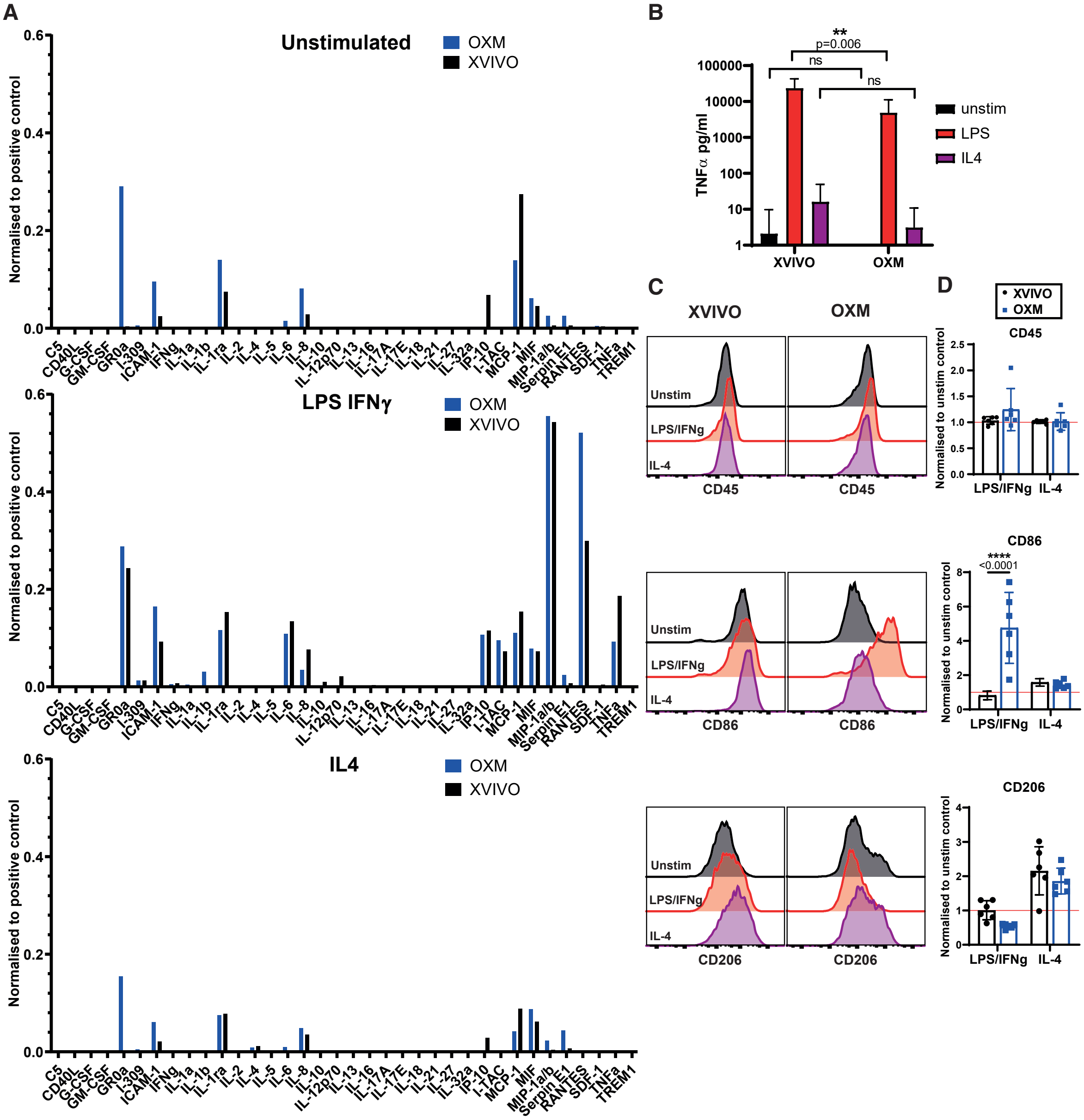

Figure 5. Macrophage activation states between culture media

(A) Cytokine secretion into the supernatant under resting conditions (top), or stimulated with 100 ng/mL LPS + 20 ng/mL IFN-γ (middle) or 50 ng/mL IL-4 (bottom) normalized to a positive control. n = 1 experiment.

(B) TNF-α secretion from unstimulated (black), or 100 ng/mL LPS (red) or 50 ng/mL IL-4 (purple) stimulated cells. Mean ± SD, n = 5 experiments in each of 3 independent donors' cell lines.

(C) Surface expression of CD45, CD86, or CD206 measured by flow cytometry after stimulation, colored according to (B). Histograms show fluorescence intensity (x axis) normalized to the mode (y axis).

(D) Geometric MFI relative to unstimulated control.

Mean ± SD, n = 3 experiments in each of 2 independent donors' cell lines. (B–D) Significance was calculated by two-way ANOVA, Sidak's multiple comparison test. Significance is shown when $*p < 0.05$, $**p < 0.01$, $***p < 0.001$, $****p < 0.0001$.

are restricted to lentiviruses, we also assessed replication ZIKV strain MR-766 (Uganda, 1947) in these cells. Cells produced in both media released virus after an eclipse phase of ~6 h post infection with no difference in output titer ([3.36 ± 1.83] $\times 10^7$ pfu/mL XVIVO, [2.26 ± 1.14] $\times 10^7$ pfu/mL OXM at 48 h post infection; Figure 6C).

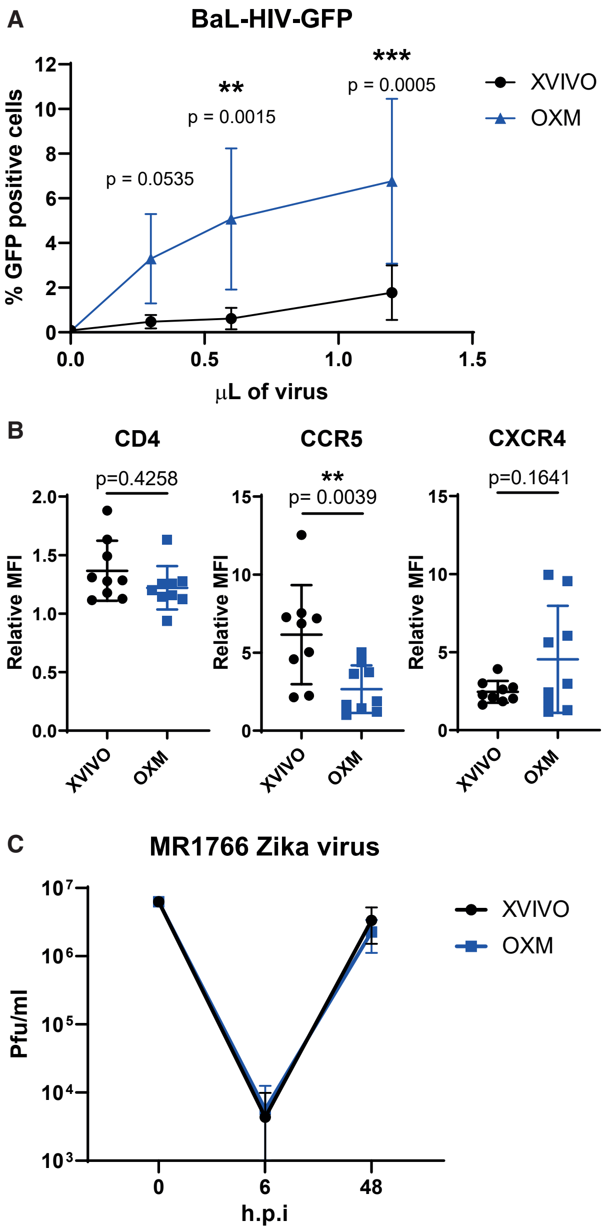

Figure 6. Macrophage infection by HIV-1 or Zika virus under different culture conditions

(A) Percentage of macrophages infected with a GFP-encoding, single-round HIV-1 pseudotyped virus measured by flow cytometry.
(B) CD4, CCR5, and CXCR4 surface expression measured by flow cytometry shown as ratio of geometric MFI to the isotype control.
(C) MR1766 Zika virus titer from infected macrophages shown in plaque-forming units (pfu)/mL.
Significance was calculated by (A) two-way ANOVA, Sidak's multiple comparison test, (B) Wilcoxon matched-pairs signed rank t test.
(A–C) Mean ± SD n = 4 experiments in each of 2 independent donors' cell lines. Significance is shown when $*p < 0.05$, $** p < 0.01$, $*** p < 0.001$.

Therefore, OXM-cultured macrophages are a good model for HIV-1 infection, and differences in viral susceptibility between culturing conditions are not pan-viral.

DISCUSSION

The need to define an open-source medium for differentiating macrophages from iPSC was driven by two requirements. Firstly, to understand the finer changes in macrophage response to stimuli we must have a clear understanding of the resting conditions of these cells. Secondly, LC-MS analysis indicated the potential presence of several undisclosed molecules in the commercial serum-free alternative medium, XVIVO 15, some of which could inhibit, or polarize the macrophage response if present. Our analysis putatively identified isolariciresinol 9′-O-alpha-L-arabinofuranoside, a lignan glycoside, which is known to have anti-inflammatory effects (Liu et al., 2018), as well as methyl glucosinolate and dithionous acid. While the precise functions of these molecules are unknown, it is worth noting that indole glucosinolates have been reported to have potent anti-inflammatory effects (Vo et al., 2014). In its ionized form (dithionite), dithionous acid is a strong reducing agent capable of affecting the redox state of heme-containing enzymes, such as cytochrome *b*, which may affect cell physiology (Berton et al., 1986).

We found that basing our new medium, OXM, on aDMEM/F-12, did not prevent differentiation of macrophages from iPSCs. However, we did see considerable differences in both yield and morphology. OXM-cultured cells were significantly more adherent, with the increased expression of adherence-related genes. This appeared to limit the harvestability of the cells cultured in OXM to once per week, most probably due to inducing cell adherence to plastic seen when cultured in fresh differentiation medium, but does not explain the observed reduced yield of macrophages cultured in this medium. We have previously shown that XVIVO-cultured iPSC macrophages are significantly larger than blood MDMs (van Wilgenburg et al., 2013), which are closer in size to OXM-cultured cells. Considering the very high levels of glucose in XVIVO medium, we suspected that there could be major differences in cell metabolism. We were therefore unsurprised to see that metabolism-related GO terms were among the most abundant terms differentially enriched between the media types. Whereas OXM-cultured cells were enriched for triglyceride homeostasis, reverse cholesterol transport and peptide catabolic process, XVIVO-cultured cells upregulated many genes involved in lipid metabolism and cholesterol biosynthesis, such as fatty acid synthase (*FASN*), 7-dehydrocholesterol reductase (*DHCR7*), and acetyl-CoA acetyltransferase 2 (*ACAT2*).

Macrophage metabolism differs substantially between activated states (M1 versus M2), with fatty acid synthesis upregulated in M1 cells and fatty acid oxidation upregulated in M2 cells (Caputa et al., 2019). Unsurprisingly then, we observed that XVIVO-cultured cells had higher expression of markers of classical M1 (pro-inflammatory) polarization (Boyette et al., 2017; Mosser, 2003). It is possible that this activated state in XVIVO is responsible for their relative insensitivity to LPS and interferon γ-induced changes in CD86 expression, unlike OXM-cultured cells, which displayed significant changes in this marker, although this does not appear to impact the cytokine response by these cells. Conversely, OXM-cultured cells showed similarities to non-classical or inflammatory monocytes, with increased expression of CD16. This potential pro-inflammatory phenotype may therefore be linked to the constitutive secretion of some inflammatory cytokines, such as IL-6 and GROα, but its importance in cellular function is unclear. Nevertheless, this cytokine profile observed is consistent with our previous work (Haenseler et al., 2017b) in which iPSC-derived microglia released similar cytokines in monoculture that were reduced when co-cultured with neurons. This suggests that our tissue-resident macrophages likely receive anti-inflammatory signals from neighboring stromal cells as part of a regulatory feedback system. Overall, neither resting OXM- or XVIVO-cultured macrophages clearly fell into conventional phenotypes, such as "classical," "non-classical," "M1-activated," or "M2-activated." However, iPSC-derived macrophages have been shown to more closely resemble primitive macrophages, and therefore such categories, which are largely derived from work with adult blood MDMs cultured in serum-containing medium, are an imperfect system for the phenotypic characterization of these cells (Murray et al., 2014; Nahrendorf and Swirski, 2016). Instead, primitive macrophages, such as our iPSC-derived macrophages (Buchrieser et al., 2017), ought to be considered more along a spectrum of activation states, subtly influenced by nutrient availability and extracellular matrix and cell-cell interactions in the various tissue environments they may occupy (Guilliams et al., 2020; Martinez and Gordon, 2014; Murray et al., 2014).

One surprising observation was that XVIVO-cultured cells appear to be transcriptionally primed for an antiviral response. We found higher expression of many interferon-inducible genes and constitutive secretion of the interferon-inducible cytokine IP-10 (CXCL10). This clearly translated into a reduced infectivity of HIV-1 pseudotype virus in these cells compared with OXM-cultured cells, despite the cells having higher expression of the surface receptor CCR5. However, it did not result in a difference in ZIKV infection of these cells. This is supported by the fact that OXM-cultured cells had higher expression of the interferon receptors *IFNAR2* and *IFNGR2*, and therefore are likely capable of mounting a strong antiviral response. It is worth noting that, although expression of interferon genes was lower in OXM-cultured cells, it was not absent. These cells may therefore be a better model of pre-viral exposure macrophages.

To conclude, we describe the successful development of a defined, and open-source medium for culturing human iPSC-derived macrophages: OXM. Subtle differences in phenotype and function in macrophages cultured in a commercial alternative highlight the importance of transparency in culturing techniques and demonstrates how direct comparisons without validation between culturing methods may not always be appropriate. We hope that the open-source nature of this medium will enable it to be used as a foundation for future improved differentiation protocols not only for macrophages, but also other iPSC-derived cell types.

EXPERIMENTAL PROCEDURES

iPSC lines

The derivation and characterization of the iPSC lines used in this study are described elsewhere: SFC840-03-03 (Fernandes et al., 2016), SFC841-03-01 (Dafinca et al., 2016), and SFC856-03-04 (Haenseler et al., 2017b). See supplemental information for more information.

iPSC culture

iPSCs were cultured in their stated medium (Table S1) on Geltrex (Gibco, no. A1413201)-coated tissue culture dishes and passaged using TrypLE Express (Gibco, no. 12604013). For 24 h after plating, medium was supplemented with 10 μM Y-27632 (Abcam, ab120129). Cells were incubated at 37°C, 5% CO_2. See supplemental information for more information.

Macrophage differentiation

iPSCs were differentiated into macrophages using a protocol based on that described previously (van Wilgenburg et al., 2013). The updated method is as follows. AggreWell 800 plates (STEMCELL Technologies, no. 34815) were prepared by addition of 0.5 mL Anti-Adherence Rinsing Solution (STEMCELL Technologies, no. 07010) and centrifugation at 3,000 × *g* for 3 min to remove bubbles from the microwells. Rinse solution was then aspirated and replaced with 1 mL of 2× concentrated EB medium (1× EB medium: OXE8 medium supplemented with 50 ng/mL BMP4 (PeproTech, no. PHC9534), 50 ng/mL VEGF (PeproTech, no. PHC9394), and 20 ng/mL SCF (Miltenyi Biotec, no. 130-096-695) supplemented with 10 μM Y-27632. iPSCs were resuspended by washing with PBS, incubating in TrypLE Express for 3–5 min at 37°C, 5% CO_2, followed by gentle lifting in aDMEM/F-12 to achieve single-cell suspension. Cells were counted and pelleted by centrifugation at 400 × *g* for 5 min. After centrifugation, cells were resuspended at 4×10^6 cells/mL in OXE8 supplemented with 10 μM Y-27632 and 1 mL added to the AggreWell. The AggreWell plate was then

spun at 100 × g for 3 min with no braking to encourage even distribution of cells across microwells. Cells were incubated for 4 days at 37°C, 5% CO_2, with daily feeding of 75% medium change with EB medium, by aspiration of 1 mL by pipette, and gentle addition of 1 mL fresh medium twice to avoid disturbance to the microwells. After 4 days, EBs were lifted from the plate using a Pasteur pipette and passed over a 40 μm cell strainer to remove dead cells, before washing into a tissue culture plate with differentiation medium (Table S2). EBs were divided evenly into 2 T175 flasks and topped up to 20 mL with differentiation medium. Differentiation cultures were incubated at 37°C, 5% CO_2, with weekly feeding of an additional 10 mL until macrophage precursors (PreMac) cells started to be produced. After this point, PreMac cells were collected weekly and a minimum of equal volumes of media to the volume removed were replaced into the differentiation cultures. Each harvest involved a 25%–50% medium change. Harvested cells could be stored or transported on ice for up to 48 h without loss of viability, and were either used directly or plated in appropriately sized tissue culture plates for further culturing in XVIVO or OXM macrophage medium (Table S2) for a further 7 days, with a 50% medium change on day 4.

Cell count, size, and viability measurements

Cell count, size, and viability were measured using the NucleoCounter NC-3000 (ChemoMetec) after staining for live/dead cells using Solution-13 AO-DAPI stain (ChemoMetec, no. 910-3013). See the supplemental information for more details.

Cell adhesion

Cells were lifted by incubation with Accutase and remaining adherent cells stained with NucBlue for imaging and quantification of nuclei by fluorescent microscopy. See the supplemental information for more details.

Flow cytometry

Cells were stained directly without fixation in fluorescence-activated cell sorting buffer (PBS supplemented to 1% FBS, 10 μg/mL human-IgG (Sigma, no. I8640-100MG), and 0.01% sodium azide) and compared with isotype controls with the same fluorophores from the same company. Fluorescence was measured using the BD LSRFortessa X-20 (BD Biosciences) and analyzed on FlowJo version 10. See the supplemental information for more details.

Cytokine and chemokine release

Cytokine and chemokine secretion was assessed as described previously (van Wilgenburg et al., 2013) using the Proteome Profiler Human Cytokine Array Kit (R&D Systems, no. ARY005B). TNF-α was quantified by ELISA (Invitrogen, 88-7346-88) according to manufacturer's instructions. See the supplemental information for more details.

Phagocytosis

Phagocytosis was measured by uptake of zymosan A (*S. cerevisiae*) BioParticles Alexa Fluor 488 (Thermo Fisher Scientific, no. Z23373) after 30 min. Uptake was measured by measurement of fluorescence using the BD LSRFortessa X-20. See the supplemental information for more details.

HIV-1 infection assay

Seven-day differentiated macrophages were infected for 72 h with a GFP-expressing HIV-1 lentiviral vector pseudotyped with strain Ba-L envelope virus diluted in macrophage medium. Percentage infected cells was determined by measurement of fluorescence using the BD LSRFortessa X-20 flow cytometer. See the supplemental information for more details.

ZIKV infection assay

Macrophages were infected with 2×10^6 pfu of ZIKV isolate MR1766 for 4 h before replacing with fresh medium. Supernatant was collected at the stated time points and stored for later quantification of virus by plaque assaying on Vero-76 cells. See the supplemental information for more details.

RNA-seq

RNA was extracted from 1×10^6 macrophages cultured in a 6-well plate, differentiated from PreMac cells harvested at weeks 8, 10, and 12 of differentiation, and sequenced by Novogene. Results were analyzed in-house.

LC-MS analysis of media

Samples of the media were prepared for LC-MS analysis as described previously (Ebrahimi et al., 2020). See the supplemental information for more details.

Quantification of glucose concentration

Glucose concentration in the media was quantified using a modified protocol for the Glucose (HK) Assay Kit (Supelco, no. GAHK20-1KT). See the supplemental information for more details.

Statistical analysis

All statistical analysis used is reported in the figure legends and was carried out using GraphPad Prism version 8.2.1 or R version 4.0. In all cases data were considered significant when $p < 0.05$.

Data and code availability

RNA-seq data and analysis have been deposited with the NCBI Gene Expression Omnibus. Theaccession number for the data in this paper is GEO: GSE171313. See the supplemental information for more details.

SUPPLEMENTAL INFORMATION

Supplemental information can be found online at https://doi.org/10.1016/j.stemcr.2021.05.018.

AUTHOR CONTRIBUTIONS

Conceptualization, A.V.-J., S.A.C., and W.S.J.; methodology, A.V.-J. and W.S.J.; investigation, A.V.-J., K.H.E., C.B., E.P., J.G.-J., and W.S.J.; data curation, S.S.; formal analysis, A.V.-J., S.S., and K.H.E.; visualization, A.V.-J., S.S., K.H.E., and P.K.R.; supervision, P.K.R. and E.P.; writing – original draft, A.V.-J., K.H.E., and W.S.J.;

writing – review & editing, A.V.-J., S.A.C., and W.S.J.; funding acquisition, A.V.-J., S.A.C., and W.S.J.

ACKNOWLEDGMENTS

This project was principally funded by a Wellcome Trust Four-year PhD Studentship (203805/Z/16/Z). Liquid chromatography-mass spectrometry samples were kindly run in the facility of Prof. James McCullagh (Department of Chemistry). The work in the group of Prof. James McCullagh is supported by Wellcome Institutional Strategic Support Fund. Stem cell work was carried out in the James and Lillian Martin Centre for stem cell research, and has received financial support from the the Oxford Martin School (LC0910-004).

Received: November 6, 2020
Revised: May 21, 2021
Accepted: May 23, 2021
Published: June 24, 2021

REFERENCES

Abud, E.M., Ramirez, R.N., Martinez, E.S., Healy, L.M., Nguyen, C.H.H., Newman, S.A., Yeromin, A.V., Scarfone, V.M., Marsh, S.E., Fimbres, C., et al. (2017). iPSC-derived human microglia-like cells to study neurological diseases. Neuron *94*, 278–293.e9.

Berton, G., Cassatella, M.A., and Ross, F. (1986). Molecular basis of macrophage activation. Expression of the low potential cytochrome b and its reduction upon cell stimulation in activated macrophages. J. Immunol. *136*, 1393–1399.

Bian, Z., Gong, Y., Huang, T., Lee, C.Z.W., Bian, L., Bai, Z., Shi, H., Zeng, Y., Liu, C., He, J., et al. (2020). Deciphering human macrophage development at single-cell resolution. Nature *582*, 571–576.

Boyette, L.B., MacEdo, C., Hadi, K., Elinoff, B.D., Walters, J.T., Ramaswami, B., Chalasani, G., Taboas, J.M., Lakkis, F.G., and Metes, Di.M. (2017). Phenotype, function, and differentiation potential of human monocyte subsets. PLoS One *12*, 1–20.

Brownjohn, P.W., Smith, J., Solanki, R., Lohmann, E., Houlden, H., Hardy, J., Dietmann, S., and Livesey, F.J. (2018). Functional studies of missense TREM2 mutations in human stem cell-derived microglia. Stem Cell Reports *10*, 1294–1307.

Buchrieser, J., James, W., and Moore, M.D. (2017). Human induced pluripotent stem cell-derived macrophages share ontogeny with MYB-independent tissue-resident macrophages. Stem Cell Reports *8*, 334–345.

Buchrieser, J., Oliva-Martin, M.J., Moore, M.D., Long, J.C.D., Cowley, S.A., Perez-Simón, J.A., James, W., and Venero, J.L. (2018). RIPK1 is a critical modulator of both tonic and TLR-responsive inflammatory and cell death pathways in human macrophage differentiation. Cell Death Dis. *9*, 973.

Cao, X., Yakala, G.K., van den Hil, F.E., Cochrane, A., Mummery, C.L., and Orlova, V.V. (2019). Differentiation and functional comparison of monocytes and macrophages from hiPSCs with peripheral blood derivatives. Stem Cell Reports *12*, 1282–1297.

Caputa, G., Flachsmann, L.J., and Cameron, A.M. (2019). Macrophage metabolism: a wound-healing perspective. Immunol. Cell Biol. *97*, 268–278.

Cech, T.R., Eddy, S.R., Eisenberg, D., Hersey, K., Holtzman, S.H., Poste, G.H., Raikhel, N.V., Scheller, R.H., Singer, D.B., and Waltham, M.C. (2003). Sharing publication-related data and materials: responsibilities of authorship in the life sciences. Plant Physiol. *132*, 19–24.

Chen, G., Gulbranson, D., Hou, Z., Bolin, J., Ruotti, V., Probasco, M., Smuga-Otto, K., Howden, S., Diol, N., Propson, N., et al. (2011). Chemically defined conditions for human iPSC derivation and culture. Nat. Methods *8*, 424–429.

Dafinca, R., Scaber, J., Ababneh, N., Lalic, T., Weir, G., Christian, H., Vowles, J., Douglas, A.G.L., Fletcher-Jones, A., Browne, C., et al. (2016). C9orf72 hexanucleotide expansions are associated with altered endoplasmic reticulum calcium homeostasis and stress granule formation in induced pluripotent stem cell-derived neurons from patients with amyotrophic lateral sclerosis and frontotemporal demen. Stem Cells *34*, 2063–2078.

Ebrahimi, K.H., Howie, D., Rowbotham, J.S., McCullagh, J., Armstrong, F.A., and James, W.S. (2020). Viperin, through its radical-SAM activity, depletes cellular nucleotide pools and interferes with mitochondrial metabolism to inhibit viral replication. FEBS Lett. *594*, 1624–1630.

Fernandes, H.J.R., Hartfield, E.M., Christian, H.C., Emmanoulidou, E., Zheng, Y., Booth, H., Bogetofte, H., Lang, C., Ryan, B.J., Sardi, S.P., et al. (2016). ER stress and autophagic perturbations lead to elevated extracellular α-synuclein in GBA-N370S Parkinson's iPSC-derived dopamine neurons. Stem Cell Reports *6*, 342–356.

Garcia-Reitboeck, P., Phillips, A., Piers, T.M., Houlden, H., Hardy, J., and Pocock, J.M. (2018). Human induced pluripotent stem cell-derived microglia-like cells harboring TREM2 missense mutations show specific deficits in phagocytosis. Cell Rep. *24*, 2300–2311.

Ginhoux, F., and Guilliams, M. (2016). Tissue-resident macrophage ontogeny and homeostasis. Immunity *44*, 439–449.

Gordon, S., and Plüddemann, A. (2017). Tissue macrophages: heterogeneity and functions. BMC Biol. *15*, 1–18.

Gordon, S., Plüddemann, A., and Martinez Estrada, F. (2014). Macrophage heterogeneity in tissues: phenotypic diversity and functions. Immunol. Rev. *262*, 36–55.

Guilliams, M., Thierry, G.R., Bonnardel, J., and Bajenoff, M. (2020). Establishment and maintenance of the macrophage niche. Immunity *52*, 434–451.

Haenseler, W., Zambon, F., Lee, H., Vowles, J., Rinaldi, F., Duggal, G., Houlden, H., Gwinn, K., Wray, S., Luk, K.C., et al. (2017a). Excess α-synuclein compromises phagocytosis in iPSC-derived macrophages. Sci. Rep. *7*, 9003.

Haenseler, W., Sansom, S.N., Buchrieser, J., Newey, S.E., Moore, C.S., Nicholls, F.J., Chintawar, S., Schnell, C., Antel, J.P., Allen, N.D., et al. (2017b). A highly efficient human pluripotent stem cell microglia model displays a neuronal-Co-culture-specific expression profile and inflammatory response. Stem Cell Reports *8*, 1727–1742.

Härtlova, A., Herbst, S., Peltier, J., Rodgers, A., Bilkei-Gorzo, O., Fearns, A., Dill, B.D., Lee, H., Flynn, R., Cowley, S.A., et al. (2018). LRRK2 is a negative regulator of *Mycobacterium tuberculosis* phagosome maturation in macrophages. EMBO J. *37*, e98694.

Honeycutt, J.B., Wahl, A., Baker, C., Spagnuolo, R.A., Foster, J., Zakharova, O., Wietgrefe, S., Caro-vegas, C., Madden, V., Sharpe, G., et al. (2016). Macrophages sustain HIV replication in vivo independently of T cells. J. Clin. Invest. *126*, 1353–1366.

Honeycutt, J.B., Thayer, W.O., Baker, C.E., Ribeiro, R.M., Lada, S.M., Cao, Y., Cleary, R.A., Hudgens, M.G., Richman, D.D., and Garcia, J.V. (2017). HIV persistence in tissue macrophages of humanized myeloid-only mice during antiretroviral therapy. Nat. Med. *23*, 638–643.

Jiang, Y., Cowley, S.A., Siler, U., Melguizo, D., Tilgner, K., Browne, C., Dewilton, A., Przyborski, S., Saretzki, G., James, W.S., et al. (2012). Derivation and functional analysis of patient-specific induced pluripotent stem cells as an in vitro model of chronic granulomatous disease. Stem Cells *30*, 599–611.

Karlsson, K.R., Cowley, S., Martinez, F.O., Shaw, M., Minger, S.L., and James, W. (2008). Homogeneous monocytes and macrophages from human embryonic stem cells following coculture-free differentiation in M-CSF and IL-3. Exp. Hematol. *36*, 1167–1175.

Lee, H., Flynn, R., Sharma, I., Haberman, E., Carling, P.J., Nicholls, F.J., Stegmann, M., Vowles, J., Haenseler, W., Wade-Martins, R., et al. (2020). LRRK2 is recruited to phagosomes and co-recruits RAB8 and RAB10 in human pluripotent stem cell-derived macrophages. Stem Cell Reports *14*, 940–955.

Liu, L.T., Liang, L., Wang, W., Yan, C.Q., Zhang, J., Xiao, Y.C., Ye, L., Zhao, M.X., Huang, Q.S., Bian, J.J., et al. (2018). Isolariciresinol-9′-O-α-L-arabinofuranoside protects against hydrogen peroxide-induced apoptosis of human umbilical vein endothelial cells via a PI3K/Akt/Bad-dependent pathway. Mol. Med. Rep. *17*, 488–494.

Martinez, F.O., and Gordon, S. (2014). The M1 and M2 paradigm of macrophage activation: time for reassessment. F1000prime Rep. *6*, 1–13.

Morey, R.D., Chambers, C.D., Etchells, P.J., Harris, C.R., Hoekstra, R., Lakens, D., Lewandowsky, S., Morey, C.C., Newman, D.P., Schönbrodt, F.D., et al. (2016). The Peer Reviewers' Openness Initiative: incentivizing open research practices through peer review. R. Soc. Open Sci. *3*, 150547.

Mosser, D.M. (2003). The many faces of macrophage activation. J. Leukoc. Biol. *73*, 209–212.

Murray, P.J., Allen, J.E., Biswas, S.K., Fisher, E.A., Gilroy, D.W., Goerdt, S., Gordon, S., Hamilton, J.A., Ivashkiv, L.B., Lawrence, T., et al. (2014). Macrophage activation and polarization: nomenclature and experimental guidelines. Immunity *41*, 14–20.

Nahrendorf, M., and Swirski, F.K. (2016). Abandoning M1/M2 for a network model of macrophage function. Circ. Res. *119*, 414–417.

National Academy of Sciences; National Academy of Engineering (US) and Institute of Medicine (US) Committee on Science, Engineering, and Public Policy (2009). On Being a Scientist: A Guide to Responsible Conduct in Research, Third Edition (National Academies Press).

Rajab, N., Rutar, M., Laslett, A.L., and Wells, C.A. (2018). Designer macrophages: pitfalls and opportunities for modelling macrophage phenotypes from pluripotent stem cells. Differentiation *104*, 42–49.

Roszer, T. (2015). Understanding the mysterious M2 macrophage through activation markers and effector mechanisms. Mediators Inflamm. *2015*, 16–18.

Rowe, R.G., Mandelbaum, J., Zon, L.I., and Daley, G.Q. (2016). Engineering hematopoietic stem cells: lessons from development. Cell Stem Cell *18*, 707–720.

Steinman, R.M., and Moberg, C.L. (1994). Zanvil Alexander Cohn 1926–1993. J. Exp. Med. *179*, 1–30.

Vanhee, S., De Mulder, K., Van Caeneghem, Y., Verstichel, G., Van Roy, N., Menten, B., Velghe, I., Philippé, J., De Bleser, D., Lambrecht, B.N., et al. (2015). In vitro human embryonic stem cell hematopoiesis mimics MYB-independent yolk sac hematopoiesis. Haematologica *100*, 157–166.

Vo, Q.V., Trenerry, C., Rochfort, S., Wadeson, J., Leyton, C., and Hughes, A.B. (2014). Synthesis and anti-inflammatory activity of indole glucosinolates. Bioorg. Med. Chem. *22*, 856–864.

van Wilgenburg, B., Browne, C., Vowles, J., and Cowley, S.A. (2013). Efficient, long term production of monocyte-derived macrophages from human pluripotent stem cells under partly-defined and fully-defined conditions. PLoS One *8*, e71098.

Stem Cell Reports, Volume *16*

Supplemental Information

Differentiation of human induced pluripotent stem cells to authentic macrophages using a defined, serum-free, open-source medium

Alun Vaughan-Jackson, Szymon Stodolak, Kourosh H. Ebrahimi, Cathy Browne, Paul K. Reardon, Elisabete Pires, Javier Gilbert-Jaramillo, Sally A. Cowley, and William S. James

Supplemental Items

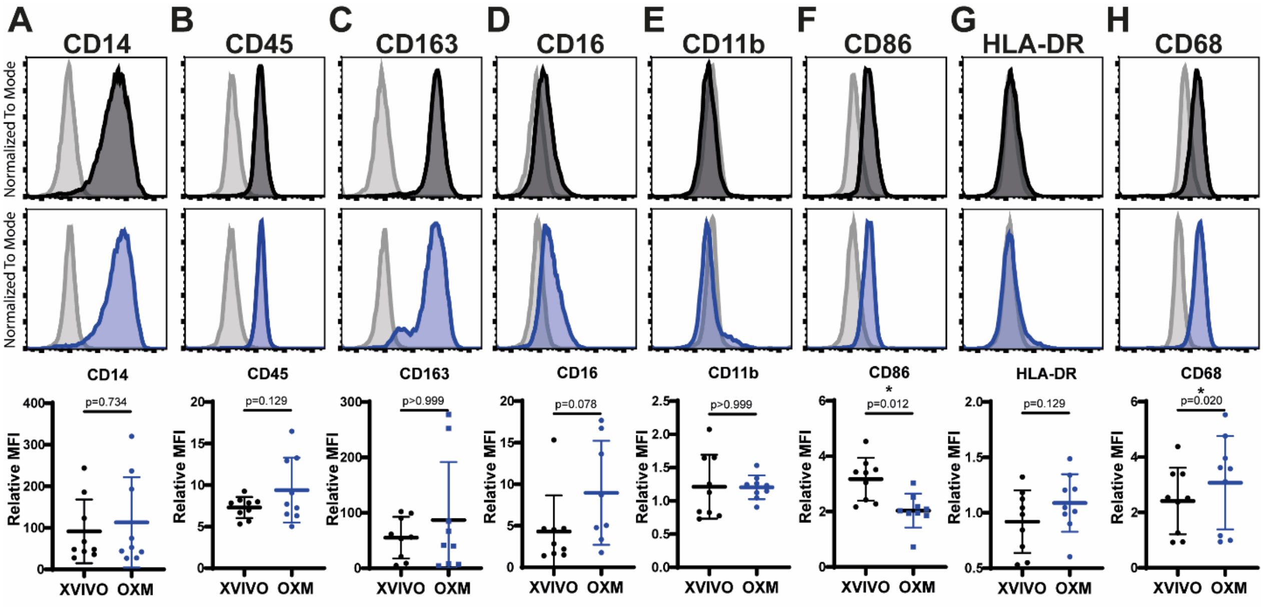

Figure S1

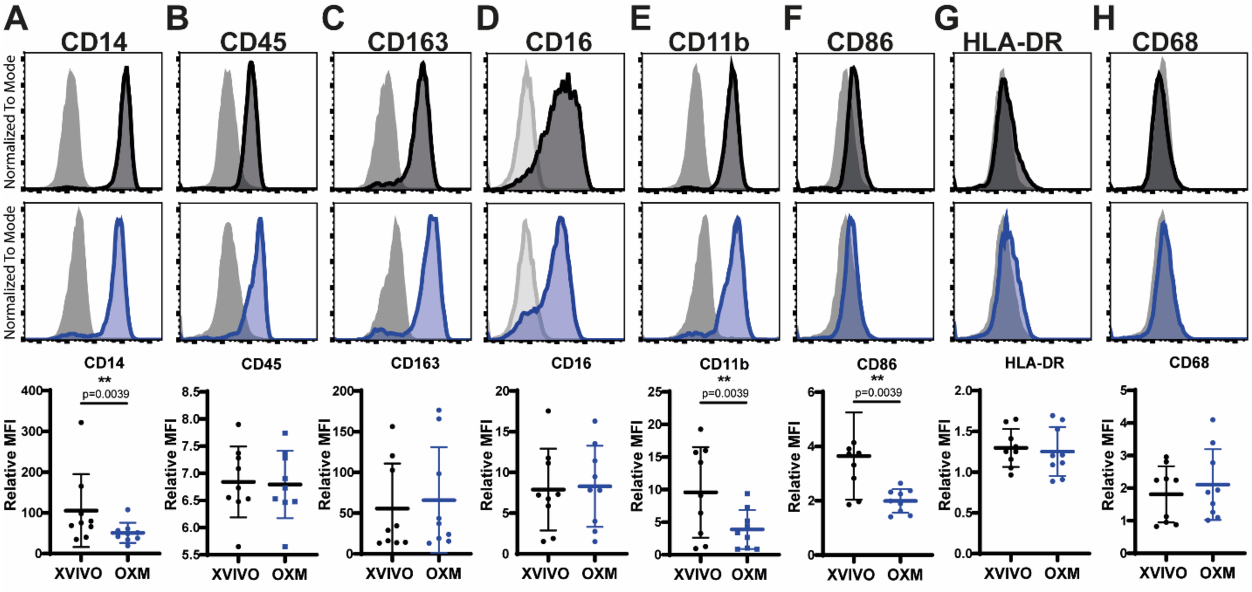
A CD14
B CD45
C CD163
D CD16
E CD11b
F CD86
G HLA-DR
H CD68
Normalized To Mode
Relative MFI
XVIVO
OXM
**
p=0.0039

Figure S2

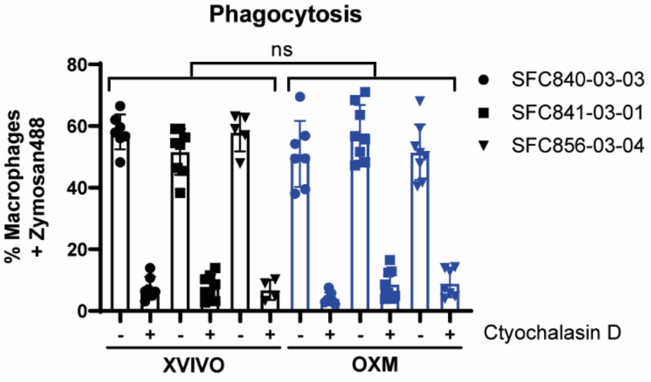
Phagocytosis
ns
80
60
40
20
0
% Macrophages + Zymosan488
SFC840-03-03
SFC841-03-01
SFC856-03-04
- + - + - + - + - + - +
Ctyochalasin D
XVIVO
OXM

Figure S3

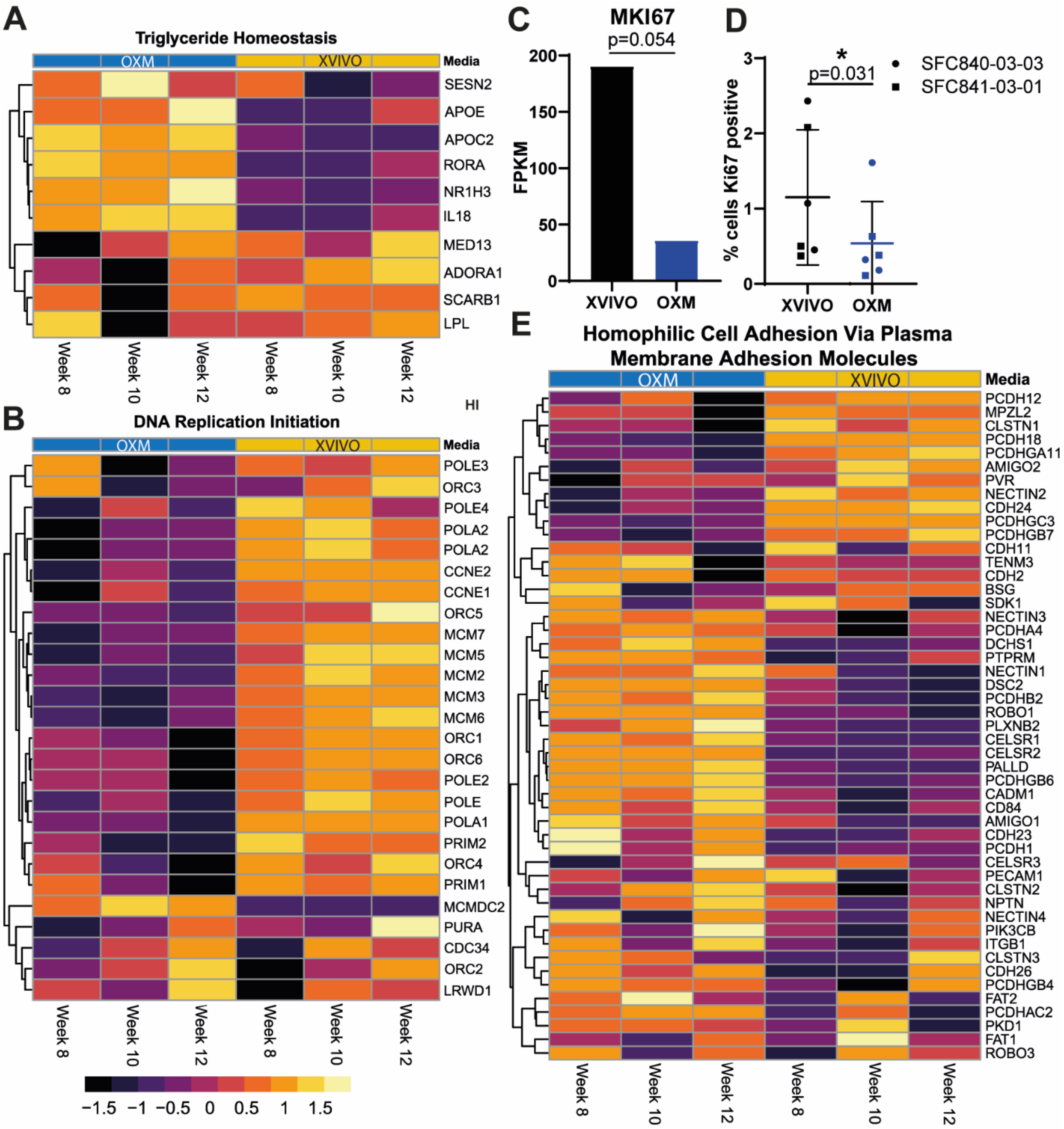
A
Triglyceride Homeostasis
OXM
XVIVO
Media
SESN2
APOE
APOC2
RORA
NR1H3
IL18
MED13
ADORA1
SCARB1
LPL
Week 8
Week 10
Week 12
B
DNA Replication Initiation
HI
POLE3
ORC3
POLE4
POLA2
POLA2
CCNE2
CCNE1
ORC5
MCM7
MCM5
MCM2
MCM3
MCM6
ORC1
ORC6
POLE2
POLE
POLA1
PRIM2
ORC4
PRIM1
MCMDC2
PURA
CDC34
ORC2
LRWD1
−1.5 −1 −0.5 0 0.5 1 1.5
C
MKI67
p=0.054
FPKM
XVIVO
OXM
D
p=0.031
*
% cells Ki67 positive
SFC840-03-03
SFC841-03-01
E
Homophilic Cell Adhesion Via Plasma Membrane Adhesion Molecules
PCDH12
MPZL2
CLSTN1
PCDH18
PCDHGA11
AMIGO2
PVR
NECTIN2
CDH24
PCDHGC3
PCDHGB7
CDH11
TENM3
CDH2
BSG
SDK1
NECTIN3
PCDHA4
DCHS1
PTPRM
NECTIN1
DSC2
PCDHB2
ROBO1
PLXNB2
CELSR1
CELSR2
PALLD
PCDHGB6
CADM1
CD84
AMIGO1
CDH23
PCDH1
CELSR3
PECAM1
CLSTN2
NPTN
NECTIN4
PIK3CB
ITGB1
CLSTN3
CDH26
PCDHGB4
FAT2
PCDHAC2
PKD1
FAT1
ROBO3

Figure S4

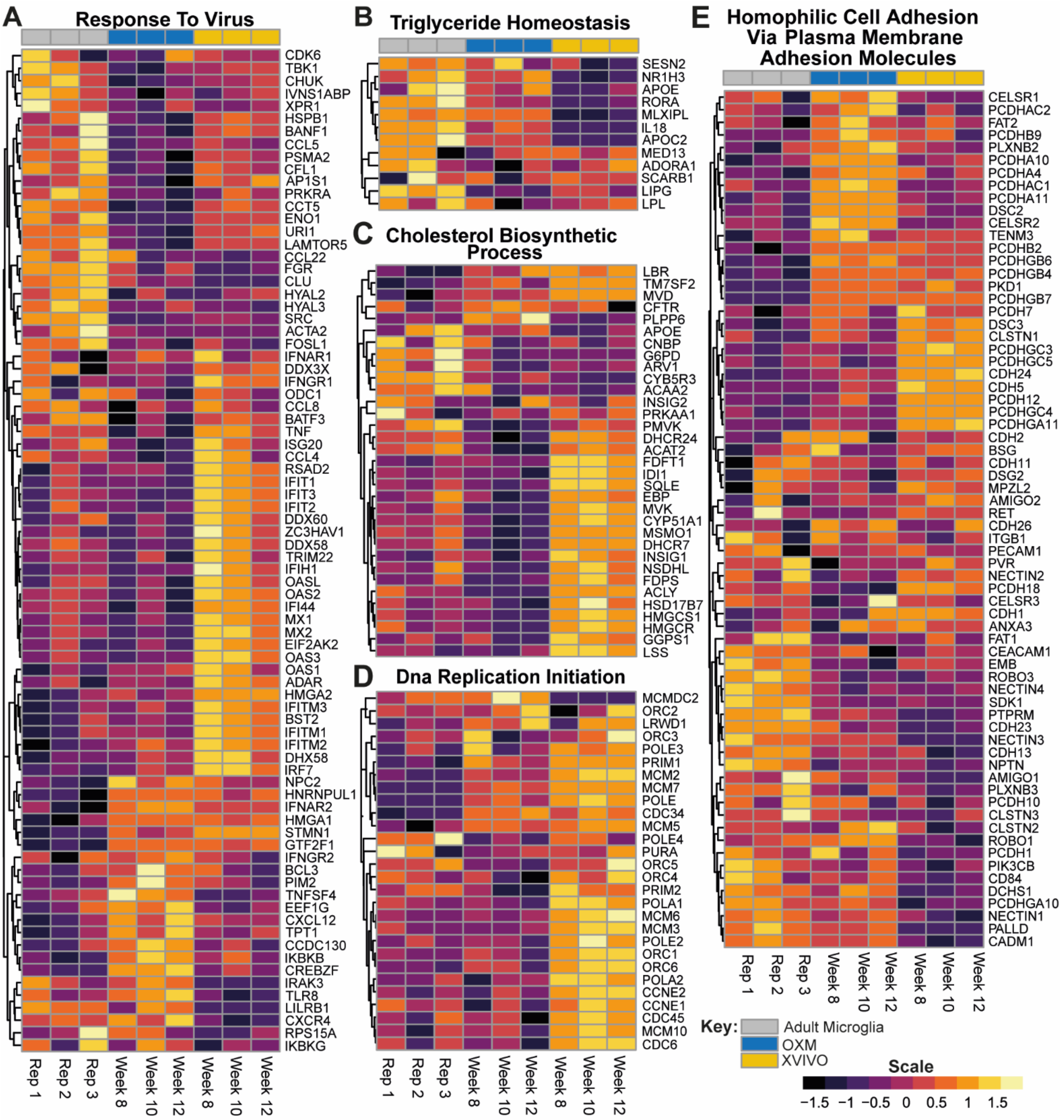
A
Response To Virus
CDK6
TBK1
CHUK
IVNS1ABP
XPR1
HSPB1
BANF1
CCL5
PSMA2
CFL1
AP1S1
PRKRA
CCT5
ENO1
URI1
LAMTOR5
CCL22
FGR
CLU
HYAL2
HYAL3
SRC
ACTA2
FOSL1
IFNAR1
DDX3X
IFNGR1
ODC1
CCL8
BATF3
TNF
ISG20
CCL4
RSAD2
IFIT1
IFIT3
IFIT2
DDX60
ZC3HAV1
DDX58
TRIM22
IFIH1
OASL
OAS2
IFI44
MX1
MX2
EIF2AK2
OAS3
OAS1
ADAR
HMGA2
IFITM3
BST2
IFITM1
IFITM2
DHX58
IRF7
NPC2
HNRNPUL1
IFNAR2
HMGA1
STMN1
GTF2F1
IFNGR2
BCL3
PIM2
TNFSF4
EEF1G
CXCL12
TPT1
CCDC130
IKBKB
CREBZF
IRAK3
TLR8
LILRB1
CXCR4
RPS15A
IKBKG
Rep 1
Rep 2
Rep 3
Week 8
Week 10
Week 12
Week 8
Week 10
Week 12
B
Triglyceride Homeostasis
SESN2
NR1H3
APOE
RORA
MLXIPL
IL18
APOC2
MED13
ADORA1
SCARB1
LIPG
LPL
C
Cholesterol Biosynthetic Process
LBR
TM7SF2
MVD
CFTR
PLPP6
APOE
CNBP
G6PD
ARV1
CYB5R3
ACAA2
INSIG2
PRKAA1
PMVK
DHCR24
ACAT2
FDFT1
IDI1
SQLE
EBP
MVK
CYP51A1
MSMO1
DHCR7
INSIG1
NSDHL
FDPS
ACLY
HSD17B7
HMGCS1
HMGCR
GGPS1
LSS
D
Dna Replication Initiation
MCMDC2
ORC2
LRWD1
ORC3
POLE3
PRIM1
MCM2
MCM7
POLE
CDC34
MCM5
POLE4
PURA
ORC5
ORC4
PRIM2
POLA1
MCM6
MCM3
POLE2
ORC1
ORC6
POLA2
CCNE2
CCNE1
CDC45
MCM10
CDC6
Rep 1
Rep 2
Rep 3
Week 8
Week 10
Week 12
Week 8
Week 10
Week 12
E
Homophilic Cell Adhesion Via Plasma Membrane Adhesion Molecules
CELSR1
PCDHAC2
FAT2
PCDHB9
PLXNB2
PCDHA10
PCDHA4
PCDHAC1
PCDHA11
DSC2
CELSR2
TENM3
PCDHB2
PCDHGB6
PCDHGB4
PKD1
PCDHGB7
PCDH7
DSC3
CLSTN1
PCDHGC3
PCDHGC5
CDH24
CDH5
PCDH12
PCDHGC4
PCDHGA11
CDH2
BSG
CDH11
DSG2
MPZL2
AMIGO2
RET
CDH26
ITGB1
PECAM1
PVR
NECTIN2
PCDH18
CELSR3
CDH1
ANXA3
FAT1
CEACAM1
EMB
ROBO3
NECTIN4
SDK1
PTPRM
CDH23
NECTIN3
CDH13
NPTN
AMIGO1
PLXNB3
PCDH10
CLSTN3
CLSTN2
ROBO1
PCDH1
PIK3CB
CD84
DCHS1
PCDHGA10
NECTIN1
PALLD
CADM1
Rep 1
Rep 2
Rep 3
Week 8
Week 10
Week 12
Week 8
Week 10
Week 12
Key:
Adult Microglia
OXM
XVIVO
Scale
−1.5
−1
−0.5
0
0.5
1
1.5

Figure S5

Figure Legends

Figure S1: Macrophage total marker phenotype. Related to Figure 3.

Total expression of A) CD14, B) CD45, C) CD163, D) CD-16, E) CD11b, F) CD86, G) HLA-DR, and H) CD68 as measured by flow cytometry in macrophages. Histograms show fluorescence intensity (x-axis) normalised to the mode (y-axis) for macrophages cultured in XVIVO (black) or OXM (Blue), relative to the isotype control (grey). Dot plots show ratio of the geometric MFI compared to the isotype control. Bars display mean ± SD. n=12 across 3 independent cell lines. Significance was calculated by Wilcoxon matched-pairs signed rank t-test. Significance is shown when p<0.05 (*), <0.01 (**), <0.001 (***)

Figure S2: PreMac surface marker phenotype. Related to Figure 3.

Surface expression of A) CD14, B) CD45, C) CD163, D) CD-16, E) CD11b, F) CD86, G) HLA-DR, and H) CD68 as measured by flow cytometry on PreMac cells. Histograms show fluorescence intensity (x-axis) normalised to the mode (y-axis) for macrophages cultured in XVIVO (black) or OXM (Blue), relative to the isotype control (grey). Dot plots show ratio of the geometric MFI compared to the isotype control. Bars display mean ± SD. n=12 across 3 independent cell lines. Significance was calculated by Wilcoxon matched-pairs signed rank t-test. Significance is shown when p<0.05 (*), <0.01 (**), <0.001 (***)

Figure S3: Phagocytosis in different culture conditions. Related to Figure 3.

Number of macrophages that are positive for Alexa-488 conjugated zymosan coated beads after 30-minute incubation, as measured by flow cytometry. Control cells were pre-treated with 10µM Cytochalasin D for 1 hour to inhibit phagocytosis. n=8 across 3 independent cell lines. Significance was calculated by 2-way ANOVA, Sidak's multiple comparison test. Significance is shown when p<0.05 (*), <0.01 (**), <0.001 (***)

Figure S4: Macrophage cell cycle state. Related to Figure 4.

A-B, E) Heatmaps of GO terms for; A) Triglyceride Homeostasis, B) DNA Replication Initiation, and E) Homophilic Cell Adhesion Via Plasma Membrane Adhesion Molecules. Results across 3 repeat measurements are shown. XVIVO in yellow, OXM in blue. The colour in each row represents the z-score of the log_2 fold difference from the mean TPM value for that gene. C) Mean TPM values for *MKI67* (KI67) expression in XVIVO vs OXM-cultured cells. D) Percentage of KI67 positive cells measured by immune-staining and flow cytometry. XVIVO in black, OXM in blue. n=6 in 2 independent cell lines. Mean and standard deviation shown. Significance was calculated by Wilcoxon matched-pairs signed rank t-test. Significance is shown when p<0.05 (*), <0.01 (**), <0.001 (***), <0.0001 (****)

Figure S5: OXM and XVIVO cultured macrophage GO terms compared to adult microglia. Related to Figure 4.

A-E) Heat maps from figures 4 and S4 here comparing gene expression data from Abud et al. 2017 Adult Microglia (grey - left), OXM (blue - middle) and XVIVO (yellow - right) for the GO terms; A) Response to Virus, B) Triglyceride Homeostasis, C) Cholesterol Biosynthetic Process, D) DNA Replication Initiation, and E) Homophilic Cell Adhesion Via Plasma Membrane Adhesion Molecules. The colour in each row represents the z-score of the log_2 fold difference from the mean TPM value for that gene.

Reference	Species	Stem Cell	Co-culture, EB, or monolayer?	# steps	Duration	Defined?	# supplemented growth factors	Serum?	Proprietary component?	Approx. Yield	Notes
Wiles and Keller, 1991	Mouse	ESC	EB	2	2-8 weeks	No	IL-3, M-CSF	10-15%	No	N/A	Primarily a haematopoiesis protocol with details on driving Macrophage differentiation
Nakano et al., 1994	Mouse	ESC	Co-culture with OP9 cells	2	10 days	No	M-CSF	20%	No	700/ stem cell	Primarily a haematopoiesis protocol with details on driving Macrophage differentiation
Moore et al., 1998	Mouse	ESC	EB	3	15-17 days	No	IL-3, M-CSF	10-15%	No	1-4/stem cell	media based on Wiles and Keller 1991
Lindmark et al., 2004	Mouse	ESC	EB	3	15-17 days	No	IL-3, M-CSF, IL-1B	10%	Yes	N/A	Based on Moore et al. 1998
Anderson et al., 2006	Human	ESC	Co-culture with S17 cells	3	30-37 days	No	GM-CSF, M-CSF	10-15%	No	N/A	
Odegaard et al., 2007	Mouse	ESC	EB	5	Infinite	No	SCF, B-estradiol, IL-3	10-15%	Yes	Infinite	Myeloid progenitors immortalised using retrovirus expressing Hox9a
Karlsson et al., 2008	Human	ESC	EB	3	2 weeks - 4 months	No	IL-3, M-CSF	10%	No	3-5x10^6/EB	Macrophages produced long term
Senju et al., 2009	Mouse	iPSC	Co-culture with OP9 cells	3	22-25 days	No	GM-CSF, M-CSF	20% & 5%	No	400-600/ stem cell	Yield for dendritic cells, not macrophages
Choi et al., 2009	Human	ESC and iPSC	Co-culture with OP9 cells	3	15-24 days	No	GM-CSF, M-CSF, IL-1B	10%	No	15 per stem cell	
Subramanian et al., 2011	Human	ESC	EB	4	21-22 days	No	SCF, IL-3, IL-6, M-CSF, BMP-4, FLT3L, G-CSF, GM-CSF, M-CSF	20%	No	0.45/ CD34+ progenitor	
Choi et al., 2011	Human	ESC and iPSC	Co-culture with OP9 cells	3	16-19 days	No	GM-CSF, M-CSF, IL-1B	10%	No	1 per stem cell	optimized protocol of Choi et al. 2009
Senju et al., 2011	Human	iPSC	Co-culture with OP9 cells	3	31-44 days	No	GM-CSF, M-CSF	10-20%	No	N/A	
	Human	iPSC	Monolayer	3	>36 days	Yes	GM-CSF, M-CSF	No	Yes	N/A	
Salvagiotto et al., 2011	Human	ESC and iPSC	Monolayer	5	18-22 days	Yes	BMP4, FGF, TPO, SCF, FLT3L, IL-6, IL-3, GM-CSF, M-CSF, IL-1B	No	Yes	3 per CD34+ progenitor	
Klimchenko et al., 2011	Human	ESC	EB	3	15-40 days	No	BMP4, VEGF, SCF, FLT3L, TPO, IL-3, GM-CSF, M-CSF	15%	No	300,000	
Kambal et al., 2011	Human	iPSC	Co-culture with OP9 cells	3	>9 days	No	GM-CSF, M-CSF	10%	Yes	N/A	method based on Vodyanik et al., 2005
Zhuang et al., 2012	Mouse	ESC	EB	3	10-30 days	No	IL-3, M-CSF	10%	No	20-40/stem cell	

Author	Species	Stem Cell	Co-culture, EB, or monolayer?	# steps	Duration	Defined?	# supplemented growth factors	Serum?	Proprietary component?	Approx. Yield	Notes
Yanagimachi et al., 2013	Human	iPSC	Monolayer	5	22-38 days	No	BMP4, VEGF, SCF, FGF, IL-3, TPO, FLT3L, GM-CSF, M-CSF	10%	Yes	6x10^6/30 stem cell colonies	
van Wilgenburg et al., 2013	Human	iPSC	EB	3	2 weeks - 12 months	Yes	VEGF, BMP4, SCF, FGF, IL-3, M-CSF	No	Yes	50/ stem cell	Based on Karlsson et al. 2008 method.
Brault et al., 2014	Human	iPSC	Co-culture with OP9 cells	4	25-31 days	No	GM-CSF, M-CSF	10%	No	3/ stem cell	
Schwartz et al., 2015	Human	iPSC	Monolayer	6	17-25 days	No*	BMP4, Activin A, FGF, VEGF, TPO, SCF, GM-CSF, M-CSF, IL-1B	10%	No	N/A	*Haematopoietic progenitor differentiation in fully defined medium from Uenishi et al., 2014
Lachmann et al., 2015	Human	iPSC	EB	3	17 days - 5 months	No	FGF, IL-3, M-CSF	10%	No	1x10^6/week	
Zhang et al., 2015	Human	iPSC	EB	5	22 days	No	BMP4, VEGF, SCF, TPO, FLT3L, FGF, M-CSF	20%	Yes	2x10^6/6-well plate	
Mucci et al., 2016	Mouse	iPSC	EB	3	18-28 days	No	IL-3, SCF	10-15%	No	10/ stem cell	similar protocol to Lachmann et al. 2015
Takata et al., 2017	Mouse	iPSC	Monolayer	4	11-13 days	Yes	FGF, BMP4, Activin A, VEGF, SCF, IL-3, M-CSF	No	Yes	N/A	
	Human	iPSC	Monolayer	6	23 days	Yes	BMP4, VEGF, CHIR99021*, FGF, SCF, DKK1, IL-3, IL-6, M-CSF	No	Yes	10-20/stem cell	*CHIR99021 is a WNT agonist
Hansen et al., 2018	Human	iPSC	Monolayer	5	27-28 days	Yes	VEGF, BMP4, IL-3, IL-6, TPO, SCF, FLT-3, G-CSF, GM-CSF	No	Yes	2000/ stem cell	to make myeloid cells - not just macrophages
Ackermann et al., 2018	Human	iPSC	EB	4	10 days - 7 weeks	No	IL-3, M-CSF	10%	Yes	1-3x10^7/week	Similar method to Lachmann et al 2015
Mukherjee et al., 2018	Human	iPSC	EB	3	4 weeks – 8 months	No	IL-3, M-CSF	10%	Yes	N/A	Based on van Wilgenburg et al. 2013
Cao et al., 2019	Human	iPSC	Monolayer	5	19 days	Yes	BMP4, Activin A, CHIR99021*, VEGF, FGF, SCF, SB431542**, IL-6, TPO, IL-3, M-CSF	No	No	30-40/ stem cell	Media based on Uenishi et al. 2014.<br>* CHIR99021 - WNT agonist.<br>†SB431542 - TGFb inhibitor
Monkley et al., 2020	Human	iPSC	EB	5	13-39 days	No	BMP4, VEGF, SCF, FGF, IL-3, TPO, FLT3L, GM-CSF, M-CSF	10%	Yes	~40/stem cell	Based on Yanagimachi et al 2013
Vaughan-Jackson et al.	*Human*	*iPSC*	*EB*	*3*	*2 weeks - 4 months*	*Yes*	VEGF, BMP4, SCF, FGF, IL-3, M-CSF	*No*	*No*	*20-30/ stem cell*	*Method based on van Wilgenburg et al. 2013*

Table S1: Methodologies for differentiation of stem cells to macrophages. Related to the Introduction.

A list of main publications describing novel methods for differentiation of macrophages from stem cells. Not including specialised macrophage subsets e.g. microglia. Reviews including these can be found elsewhere (Lee et al., 2018; Rajab et al., 2018).

Table S2: iPSC and macrophage culture media. Related to Figure 1 and 2.

Media	Component	Final Concentration	Supplier	Catalogue number
**OXE8**	Advanced DMEM/F-12	97.4%	Gibco	12634010
	GlutaMAX (100x)	2mM	Gibco	35050-038
	Heparin solution 0.2%	100ng/mL	STEMCELL™	07980
	Ascorbic Acid-2-phosphate magnesium salt	0.22mM	Sigma Aldrich	A8960
	HEPES 1M pH 7.4	15mM	Gibco	15630080
	FGF-2 basic 145aa	100ng/mL	Bio-Techne (R&D Systems)	4114-TC-01M
	TGF-beta	2ng/mL	Peprotech	AF-100-21C
**XVIVO differentiation medium**	X-VIVO 15	97.8%	SLS (Lonza)	BE02-060F
	GlutaMAX (100x)	2mM	Gibco	35050-038
	2-Mercaptoethanol (1000x)	50uM	Gibco	31350-010
	IL-3	25ng/mL	Invitrogen	PHC0033
	M-CSF	50ng/mL	Invitrogen	PHC9501
	Penicillin-Streptomycin (P/S 100x)	1%	Gibco	15140-122
**XVIVO Macrophage medium**	X-VIVO 15	97.9%	SLS (Lonza)	BE02-060F
	GlutaMAX (100x)	2mM	Gibco	35050-038
	M-CSF	50ng/mL	Invitrogen	PHC9501
	Penicillin-Streptomycin (P/S 100x)	1%	Gibco	15140-122
**OXM differentiation medium**	Advanced DMEM/F-12	96.3%	Gibco	12634010
	GlutaMAX (100x)	2mM	Gibco	35050-038
	HEPES 1M pH 7.4	15mM	Gibco	15630080
	Human recombinant Insulin solution	5µg/mL	Sigma	I9278-5ML
	Tropolone	15µM	Sigma	T89702-1G
	IL-3	25ng/mL	Invitrogen	PHC0033
	M-CSF	50ng/mL	Invitrogen	PHC9501
	Penicillin-Streptomycin (P/S 100x)	1%	Gibco	15140-122
**OXM Macrophage medium**	Advanced DMEM/F-12	96.5%	Gibco	12634010
	GlutaMAX (100x)	2mM	Gibco	35050-038
	HEPES 1M pH 7.4	15mM	Gibco	15630080
	M-CSF	50ng/mL	Invitrogen	PHC9501
	Penicillin-Streptomycin (P/S 100x)	1%	Gibco	15140-122

Complete composition of iPSC and macrophage differentiation medium including supplier and catalogue number. Advanced DMEM/F-12 formulation can be found at the suppliers site at https://www.thermofisher.com/uk/en/home/technical-resources/media-formulation.227.html (accessed 11/10/2019

Table S3: List of metabolites present in both media. Related to Figure 1.

Most likely molecule	OXM		XVIVO			measured	theoretical
	Intensity	concentration (mol/L)	Intensity	calculated concentration (mol/L)	Ion	m/z	m/z
**Tropolone**	8.10E+05	1.50E-05	1.00E+06	1.85E-05	[M-H]	121.0293	121.0295
**Pyruvic acid**	8.00E+08	1.00E-03	6.70E+06	8.38E-06	[M-H]	87.009	87.0087
**Leucine/isoleucine**	3.00E+06	4.50E-04	nd	0.00E+00	[M-H]	130.0524	130.0509
**L-Aspartic acid**	4.70E+07	5.00E-05	nd	0.00E+00	[M-H]	132.0301	132.0302
**Glutamic acid**	1.80E+09	5.00E-05	2.00E+07	5.56E-07	[M-H]	146.0458	146.0458
**Thymidine**	7.00E+05	1.50E-06	1.40E+07	3.00E-05	[M-H]	241.083	241.083
**Phenylalanine**	1.80E+05	2.15E-04	3.00E+04	3.58E-05	[M-H]	164.0716	164.0717
**C6 sugars (1)**	2.60E+08	---	5.40E+08	---	[M-H]	179.0559	179.05611
**C6 sugars (2)**	4.90E+09	---	1.00E+07	---	[M-2H]	89.0243	89.024
**Tryptophan**	1.10E+05	4.40E+05	1.60E+07	6.40E+07	[M-H]	203.0827	203.0826

Note: the C6 sugars (1) and (2) are different because the intensity ratio (C6 (1)/C6 (2)) is different between OXM and XVIVO.

Supplemental Experimental Procedures

iPSC lines

The derivation and characterisation of the iPSC lines used in this study is described elsewhere: SFC840-03-03 (Fernandes et al., 2016), SFC841-03-01 (Dafinca et al., 2016) SFC856-03-04 (Haenseler et al., 2017)). All lines were derived from dermal fibroblasts from disease-free donors recruited through StemBANCC (Morrison et al., 2015) and the Oxford Parkinson's Disease Centre: participants were recruited to this study having given signed informed consent, which included derivation of hiPSC lines from skin biopsies (Ethics Committee: National Health Service, Health Research Authority, NRES Committee South Central, Berkshire, UK, who specifically approved this part of the study (REC 10/H0505/71)). The iPSC lines were all derived using non-integrating Sendai reprogramming vectors (Cytotune, Life Technologies), cultured in mTeSR™1 (StemCell Technologies, #85850) on hESC-qualified Matrigel-coated plates (BD, #356234), passaging as clumps using 0.5 mM EDTA in PBS (Beers et al., 2012). Large-scale, low-passage frozen SNP-QCed batches were used for experiments to ensure consistency.

Cell culture reagent preparation: OXE8

Ascorbic acid was dissolved in PBS to make a 0.22 M stock and aliquoted at 1mL for storage at -20^oC indefinitely, or 4^oC for up to 3 months. FGF-2 was reconstituted according to manufacturer's instructions and a 1 mg/mL stock made in 0.1% HSA (improves stability of storage) and aliquoted at 50 µL for storage at -80^oC. 10 µg Lyophilised TGF-β was first centrifuged to pellet the powder before reconstitution in 100 µL ultra-pure sterile water. This was then transferred into 4.8 mL 0.1% HSA solution to make a 2 µg/mL stock and aliquoted at 0.5 mL for storage at -80^oC. Both FGF-2 and TGF-β once thawed are considered stable for 2 weeks at 4^oC. Avoid freeze/thaw cycles. To make OXE8 was as follows: 500 mL aDMEM/F-12, 5 mL GlutaMAX, 25 µL Heparin, 0.5 mL Ascorbic acid, 7.5 mL HEPES pH 7.4, 50 µL FGF-2, and 0.5 mL TGF-β.

Cell culture reagent preparation: OXM

The desired mass of powdered Tropolone was dissolved in ultra-pure water and filter sterilised by passing through a 0.22 µm filter. It was aliquoted at 1.5 mL and stored at -20^oC indefinitely, or 4^oC for up to 3 months. IL-3 was reconstituted according to manufacturer's instructions in ultra-pure sterile water and aliquoted at 0.5 mL for storage at -80^oC. Once thawed, it is considered stable for 1 month at 4^oC. M-CSF was reconstituted as needed according to manufacturer's instructions in 20 mM Tris-HCL pH 8.0. Each vial was resuspended in 1 mL and could be stored at for 1 month at 4^oC or stored at -80^oC. To make OXM differentiation media was as follows: 500 mL aDMEM/F-12, 5 mL GlutaMAX, 5 mL P/S, 0.25 mL Insulin solution, 7.5 mL HEPES pH 7.4, 0.75mL Tropolone, 0.5 mL M-CSF, and 0.25 mL IL-3. To make OXM macrophage medium Tropolone and IL-3 were excluded.

Cell culture

iPSC were cultured in mTeSR™ 1 on Geltrex™ (Gibco, #A1413201)-coated tissue culture dishes and passaged using TrypLE™ Express (Gibco, #12604013). For 24 hours after plating, media was supplemented with 10 µM Y-27632 (Abcam, ab120129). Vero-76 and HEK293T cells were cultured in DMEM made to 10% FBS (Sigma, #F9665) and 1% Pen/Strep (Gibco, #15140122). All cells were incubated at 37^oC, 5% CO_2.

Macrophage differentiation

iPSC were differentiated into macrophages using a protocol based on that previously described (van Wilgenburg et al., 2013). The updated method is as follows. Aggrewell™ 800 plates (Stemcell technologies, #34815) were prepared by addition of 0.5 mL Anti-Adherence Rinsing solution (Stemcell technologies, #07010) and centrifugation at 3000g for 3 minutes to remove bubbles from the microwells. Rinse solution was then aspirated and replaced with 1 mL of 2X concentrated EB medium (1X EB media: OXE8 media supplemented with 50 ng/mL BMP4 (Peprotech, #PHC9534), 50 ng/mL VEGF (Peprotech, #PHC9394), and 20 ng/mL SCF (Miltenyi Biotec, #130-096-695) supplemented with 10 µM Y-27632. iPSC were resuspended by washing with PBS, incubating in TrypLE Express for 3-5 minutes

at 37°C, 5% CO_2, followed by gentle lifting in Advanced-DMEM/F12 to achieve single cell suspension. Cells were counted and pelleted by centrifugation at 400g for 5 minutes. After centrifugation, cells were resuspended at $4x10^6$ cells/mL in OXE8 supplemented with 10 µM Y-27632 and 1 mL added to the Aggrewell. The Aggrewell plate was then spun at 100g for 3 minutes with no braking to encourage even distribution of cells across microwells. Cells were incubated for 4 days at 37°C, 5% CO_2 with daily feeding of 75% media change with EB medium, by aspiration of 1 mL by pipette and gentle addition of 1 mL fresh media twice to avoid disturbance to the microwells. After 4 days, EBs were lifted from the plate using a Pasteur pipette and passed over a 40 µm cell strainer to remove dead cells, before washing into tissue culture plate with differentiation media (Supplementary Table 2). EBs were divided evenly into 2 T175 flasks and topped up to 20 mL with differentiation medium. Differentiation cultures were incubated at 37°C, 5% CO_2, with weekly feeding of an additional 10 mL until macrophage precursors (PreMac) cells started to be produced. After this point PreMac cells were collected weekly and a minimum of equal volumes of media to the volume removed were replaced into the differentiation cultures. Each harvest involved a 25-50% media change. Harvested cells were either used directly or plated in appropriately sized tissue culture plates for further culturing in XVIVO or OXM Macrophage medium (Supplementary Table 2) for a further 7 days, with a 50% media change on day 4.

Cell count, size, and viability measurements

Day 7 macrophages were resuspended by replacing media with StemPro™ Accutase™ Cell Dissociation Reagent (Stemcell technologies, #A1110501) and incubating 3-5 minutes at 37°C, 5% CO_2. Cells lifted in Accutase were diluted in macrophage media 1:10 to maintain accurate viability throughout measurements. Cell count, size, and viability were measured using the Nucleocounter® NC-3000 (Chemometec) after staining for live/dead cells using Solution-13 AO-DAPI stain (Chemometec, #910-3013) mixed 1:20 dye to cell suspension.

Adhesion assay

$5x10^4$ PreMacs were plate in triplicate in a 96-well plate in differentiation or macrophage medium for terminal differentiation. To assess adhesion, differentiated cells were incubated with StemPro™ Accutase™ for the indicated period of time before washing once with PBS followed by nuclear staining with NucBlue™ (Life Technologies, R37605) in live cell imaging solution (Life technologies, A14291DJ) for 15 minutes at room temperature. NucBlue™ solution was then reomoved and cells fixed in 2% PFA in PBS. Adherent cells were then counted by fluorescent imaging on the EVOS Fl auto and analysed on ImageJ. Percentage adherent cells was calculated by normalisation against an Accutase™ untreated and unwashed, stained and fixed only control.

Flow Cytometry

Cells were lifted using Accutase as described above as no difference in surface marker levels using this method versus 5 mM EDTA/12 mM Lidocaine has been observed (Carter et al 2009, Haenseler et al 2018). Cells in suspension were kept at 4°C or on ice throughout staining. For surface marker expression, cells were stained directly without fixation in FACS buffer (PBS supplemented to 1% FBS, 10 µg/mL human-IgG (Sigma, #I8640-100MG), and 0.01% Sodium azide). For total marker staining, cells were first fixed for 10 minutes in 2% PFA over ice. They were then permeabilised by treatment with 0.1% saponin in PBS for 1 hour at room temperature. Staining was then carried out in FACS buffer supplemented with 0.1% saponin. Isotype controls with the same fluorophores from the same company were used at the same concentration. Fluorescence was measured using the BD LSRFortessa™ X-20 (BD Biosciences) and analysed using FlowJo version 10. Antibodies used for flow cytometry of surface markers were all mouse primary conjugated antibodies. Antibodies used were as follows: CD4 (APC, clone 11830, R&D systems, #FAB3791A), CD11b (APC, clone ICRF44, Bio-legend, #301310) CD206 (APC, clone 15-2, Bio-legend, #321109), CCR5 (PE, clone 45531, R&D, #FAB182P), CD16 (PE, clone LNK16, Immunotools, #21279164), CD86 (PE, clone IT2.2, Bio-legend, 305405), CD163 (PE, clone 215927, R&D, #FAB1607P), CXCR4 (PE, clone 44717, R&D, FAB173P), CD14 (FITC, clone MEM-18, Immunotools, #21270143), CD45 (FITC, clone MEM-28, Immunotools, #21270453X2), CD68 (FITC, clone Ki-M7, BIO-RAD, #MCA2375F), CD86 (FITC, clone BU63, Immunotools, #21480863), HLA-DR (FITC, clone MEM-12, Immunotools, #21388993), IgG1κ (APC, clone MOPC-21, Bio-legend, #400120), IgG2a (APC, clone 20102, R&D, #IC003A), IgG1 (PE, clone PPV-06, Immunotools, #21275514),

IgG2bk (PE, clone MPC-11, Bio-legend, #400311), IgG1 (PE, clone 11711, R&D, #IC002P), IgG2b (PE, clone 133303, R&D, #IC0041P), IgG1 (FITC, clone PPV-06, Immunotools, #21335013), and IgG1 (FITC, BIO-RAD, #MCA928F). Staining for KI67 used a mouse primary antibody (Abcam, #ab15580) or isotype control primary IgG (Abcam, #ab172730) followed with a goat anti-mouse Alexa-488 conjugated secondary antibody (Invitrogen, #A-21131).

Cytokine and Chemokine release

As previously described (van Wilgenburg et al., 2013), $2.8x10^5$ macrophages plated in a 24-well plate were stimulated with 100 ng/mL LPS (Invivogen, #tlrl-eklps) and 20 ng/mL IFNγ (Gibco, #PHC4031), or 50 ng/mL IL-4 (Gibco, #PHC0044) for 16 hours after which supernatants were collected and spun at 400g for 5 minutes to remove dead and floating cells, and stored at -80C until required. Cytokine release was measured using the Proteome Profiler Human Cytokine Array Kit (R&D systems, #ARY005B) modified to use IRDye 800CW Streptavidin (LI-COR, #926-32230) according to R&D systems protocol (https://www.rndsystems.com/resources/technical/use-proteome-profiler-arrays-li-cor-detection) for visualizing results on the LI-COR Odyssey 9260 and quantification using Image Studio Lite version 5.2. To quantify TNFα, 5x104 macrophages plated in a 96-well plates were stimulated with 100 ng/mL LPS or 50 ng/mL IL-4 in 100 µL macrophage medium for 16 hours. Supernatants were spun and stored at -80°C until quantification by ELISA according to manufacturer's instructions (Invitrogen, 88-7346-88).

Phagocytosis

Macrophages were cultured at $2x10^5$ cells/well in a 24-well plate. Control cells were pre-treated for 1 hour with 10 µM Cytochalasin D (Cambridge Bioscience, #11330-1 mg-CAY). 12.5 µg Zymosan A (*S. cerevisiae*) BioParticles™ Alexa Fluor-488 (ThermoFisher, #Z23373) were added to each well of cells and incubated for 30 minutes at 37°C, 5% CO_2. Particles not taken up were quenched with 0.025% (v/v) Trypan Blue (Sigma, #T8154) in PBS before lifting the cells with Accutase as described above. Cells were fixed with 4% paraformaldehyde (PFA) (Alfa Aesar, #J61899.AK) before measuring fluorescence with the BD LSRFortessa™ X-20.

Lentivirus preparation and Infection assay

Lentiviral vectors were produced using PEI-mediated transfection of HEK 293T cells with pNL4.3R-E-eGFPT2ANef and pBa-L. Lentiviral containing supernatant was harvested at 48 hours, filtered, and concentrated 100X using PEG6000.

$5x10^4$ macrophages plated in 96-well format were infected with the stated volumes of virus diluted to 100 µL and incubated for 72 hours. Cells were resuspended by pipetting after using 5 mM EDTA in PBS at 4°C for 30 minutes, and fixed in 1% PFA. GFP positive cells were measured by flow cytometry on the BD LSRFortessa™ X-20.

One-step growth of Zika virus (ZIKV) in macrophages.

ZIKV isolate MR1766, Uganda 1947, was obtained from EVAg, the European Virus Archive. $2x10^5$ macrophages in a 24-well dish were infected with 2×10^6 pfu of ZIKV. After 4 hours at 37°C, 5% CO_2, supernatants were aspirated and replaced with 700 µL fresh media, and incubation continued. 200 µL samples of supernatant were harvested at intervals from 6h to 48 hours post-infection, clarified by centrifugation and stored at -80°C.

Titration of ZIKV infectivity.

Virus-containing supernatants were serially diluted 10-fold and 100 µL added to each well of a 24-well plate. $2.5x10^5$ Vero-76 Cells in maintenance media (DMEM containing 1% FBS and 1% Pen-Strep to maintain cell viability without proliferation) were then added. Cells were incubated with virus for 2 hours at 37°C, 5% CO_2, before adding a semi-solid overlay of 1.5% Carboxymethyl cellulose sodium salt, low viscosity (Sigma, #C5678) in Vero maintenance media and returned to 37°C, 5% CO_2. At 72 hours, the semi-solid overlay was removed, the cell monolayers washed with PBS, and plaques were revealed with Amido black stain.

RNA-seq sample preparation, library construction, and data generation

RNA was extracted from 1x10^{6} macrophages (SFC840-03-03) differentiated from PreMac cells harvested at week 8, 10, and 12 of differentiation, using the RNeasy Mini Kit (QIAGEN, # 74104) according to manufacturer's directions, including optional DNase treatment step. Samples were sent to Novogene on dry ice for analysis. RNA degradation and contamination was monitored on 1% agarose gels. RNA purity was checked using the NanoPhotometer® spectrophotometer (IMPLEN, CA, USA). RNA integrity and quantitation were assessed using the RNA Nano 6000 Assay Kit of the Bioanalyzer 2100 system (Agilent Technologies, CA, USA). A total amount of 1 µg RNA per sample was used as input material for the RNA sample preparations. Sequencing libraries were generated using NEBNext® Ultra TM RNA Library Prep Kit for Illumina® (NEB, USA) following manufacturer's recommendations and index codes were added to attribute sequences to each sample. Briefly, mRNA was purified from total RNA using poly-T oligo-attached magnetic beads. Fragmentation was carried out using divalent cations under elevated temperature in NEBNext First Strand Synthesis Reaction Buffer (5X). First strand cDNA was synthesized using random hexamer primer and M-MuLV Reverse Transcriptase (RNase H-). Second strand cDNA synthesis was subsequently performed using DNA Polymerase I and RNase H. Remaining overhangs were converted into blunt ends via exonuclease/polymerase activities. After adenylation of 3' ends of DNA fragments, NEBNext Adaptor with hairpin loop structure were ligated to prepare for hybridization. In order to select cDNA fragments of preferentially 150~200 bp in length, the library fragments were purified with AMPure XP system (Beckman Coulter, Beverly, USA). Then 3 µl USER Enzyme (NEB, USA) was used with size-selected, adaptorligated cDNA at 37 °C for 15 min followed by 5 min at 95 °C before PCR. Then PCR was performed with Phusion High-Fidelity DNA polymerase, Universal PCR primers and Index (X) Primer. At last, PCR products were purified (AMPure XP system) and library quality was assessed on the Agilent Bioanalyzer 2100 system. The clustering of the index-coded samples was performed on a cBot Cluster Generation System using PE Cluster Kit cBot-HS (Illumina) according to the manufacturer's instructions. After cluster generation, the library preparations were sequenced on an Illumina platform and paired-end reads were generated.

RNA-Seq data analysis

Raw reads were pre-processed using fastp (v.0.20.1) software (Chen et al., 2018). In addition to the default filtering parameters, polyN sequences in read tails and adapter sequences were trimmed, and mismatched base pairs were corrected by overlap analysis. Filtered and trimmed reads were mapped to a full decoy-aware transcriptome using the Gencode version 34 reference human transcriptome and GRCh38 primary assembly genome. Salmon (v.1.3.0) selective alignment was used for transcript quantification with optional flags correcting for GC and random hexamer priming biases (Srivastava et al., 2019). All downstream analyses were done using the R (v.4.0) programming language (R Core Team 2020). The raw counts were imported, scaled to the average transcript length over samples and the library size ('LengthScaledTPM'), and summarised to the gene level using the tximport package (v.1.16.0) (Soneson et al., 2016). Lowly expressed genes with length-scaled abundance estimates less than 10 in 3 samples were filtered out. Differential expression was tested using limma-voom (Law et al., 2014). GO enrichment analysis was conducted using the EGSEA package (Alhamdoosh et al., 2017). GO terms with less than 10 genes were excluded from the enrichment analysis. The background gene set used for the analysis was the total number of the unique genes observed in the experiment. A publicly available dataset of human *ex vivo* and iPSC-derived microglia was processed as above and used as a reference (Abud et al., 2017). Principal component analysis was performed with the prcomp package to represent the level of transcriptomic similarity amongst the samples from both studies. To visualise expression of genes from a particular GO term, the length-scaled gene abundance estimates from both studies were transformed to counts per million (TMM-adjusted), Z-score standardise and presented on a heatmap using the pheatmap package.

Liquid chromatography-mass spectrometry (LC-MS) analysis of media

Samples of the media were prepared for LC-MS analysis as described before (Ebrahimi et al., 2020). Briefly, 500 µL of each media was filtered using Amicon Ultracentrifugal filters (3 or 10 kDa cut off). The flow through was used for analysis of metabolites using high-resolution negative ion LC-MS (accuracy better than 5 ppm). The setting and methods were as described previously (Alldritt et al., 2019; Ebrahimi et al., 2020). The total ion chromatograms were analysed using MesReNova software. The MS peaks were putatively assigned using and the human metabolome database (HMDB).   The concentrations

in the XVIVO were estimated based on the known concentration of different metabolites in the OXM media the ratio of the intensities for each molecule (Concentration in XVIVO = Concentration in OXM*(intensity XVIVO / intensity OXM).

Quantification of glucose concentration

A modification of the protocol for the detection of glucose using the Glucose (HK) Assay Kit (Supelco, # GAHK20-1KT) from culture media was done as follows: 100 µL of culture media were diluted 1:10 and 1:20 in dH2O. 20 µL of each dilution were added onto a well of a 96-well flat-bottom cell culture treated plate. Glucose solution was freshly made up by resuspending the vial using 20 mL of dH2O. 100 µL of glucose solution were added per well. Plates were incubated at 35ºC for 15 minutes before measuring absorbance at 340 nm using a plate reader. Values were compared to known values from a standard curve. The standard curve was done using seven serials dilutions (top 2.5 mM) of media culture with known composition (Dulbecco's Modified Eagle Medium – 25 mM). Each sample was run in duplicates on the plate.

Supplemental References

Abud, E.M., Ramirez, R.N., Martinez, E.S., Healy, L.M., Nguyen, C.H.H., Newman, S.A., Yeromin, A. V., Scarfone, V.M., Marsh, S.E., Fimbres, C., et al. (2017). iPSC-Derived Human Microglia-like Cells to Study Neurological Diseases. Neuron *94*, 278-293.e9.

Ackermann, M., Kempf, H., Hetzel, M., Hesse, C., Hashtchin, A.R., Brinkert, K., Schott, J.W., Haake, K., Kühnel, M.P., Glage, S., et al. (2018). Bioreactor-based mass production of human iPSC-derived macrophages enables immunotherapies against bacterial airway infections. Nat. Commun. *9*, 1–13.

Alhamdoosh, M., Law, C.W., Tian, L., Sheridan, J.M., Ng, M., and Ritchie, M.E. (2017). Easy and efficient ensemble gene set testing with EGSEA. F1000Research *6*, 1–38.

Alldritt, I., Whitham-Agut, B., Sipin, M., Studholme, J., Trentacoste, A., Tripp, J.A., Cappai, M.G., Ditchfield, P., Devièse, T., Hedges, R.E.M., et al. (2019). Metabolomics reveals diet-derived plant polyphenols accumulate in physiological bone. Sci. Rep. *9*, 1–12.

Anderson, J.., Bandi, S., Kaufman, D.., and Akkina, R. (2006). Derivation of normal macrophages from human embryonic stem (hES) cells for applications in HIV gene therapy. Retrovirology *3*.

Brault, J., Goutagny, E., Telugu, N., Shao, K., Baquié, M., Satre, V., Coutton, C., Grunwald, D., Brion, J.P., Barlogis, V., et al. (2014). Optimized generation of functional neutrophils and macrophages from patient-specific induced pluripotent stem cells: Ex vivo models of X0-Linked, AR220- and AR470- chronic granulomatous diseases. Biores. Open Access *3*, 311–326.

Cao, X., Yakala, G.K., van den Hil, F.E., Cochrane, A., Mummery, C.L., and Orlova, V. V. (2019). Differentiation and Functional Comparison of Monocytes and Macrophages from hiPSCs with Peripheral Blood Derivatives. Stem Cell Reports *12*, 1282–1297.

Chen, S., Zhou, Y., Chen, Y., and Gu, J. (2018). Fastp: An ultra-fast all-in-one FASTQ preprocessor. Bioinformatics *34*, i884–i890.

Choi, K., Vodyanik, M.A., and Slukvin, I.I. (2009). Generation of mature human myelomonocytic cells through expansion and differentiation of pluripotent stem cell–derived lin–CD34+CD43+CD45+ progenitors. J. Clin. Invest. *119*, 2818.

Choi, K.D., Vodyanik, M., and Slukvin, I.I. (2011). Hematopoietic differentiation and production of mature myeloid cells from human pluripotent stem cells. Nat. Protoc. *6*, 296–313.

Dafinca, R., Scaber, J., Ababneh, N., Lalic, T., Weir, G., Christian, H., Vowles, J., Douglas, A.G.L., Fletcher-Jones, A., Browne, C., et al. (2016). C9orf72 Hexanucleotide Expansions Are Associated with Altered Endoplasmic Reticulum Calcium Homeostasis and Stress Granule Formation in Induced Pluripotent Stem Cell-Derived Neurons from Patients with Amyotrophic Lateral Sclerosis and Frontotemporal Demen. Stem Cells *34*, 2063–2078.

Ebrahimi, K.H., Howie, D., Rowbotham, J.S., McCullagh, J., Armstrong, F.A., and James, W.S. (2020). Viperin, through its radical-SAM activity, depletes cellular nucleotide pools and interferes with mitochondrial metabolism to inhibit viral replication. FEBS Lett. *594*, 1624–1630.

Fernandes, H.J.R., Hartfield, E.M., Christian, H.C., Emmanoulidou, E., Zheng, Y., Booth, H., Bogetofte, H., Lang, C., Ryan, B.J., Sardi, S.P., et al. (2016). ER Stress and Autophagic Perturbations Lead to Elevated Extracellular α-Synuclein in GBA-N370S Parkinson's iPSC-Derived Dopamine Neurons. Stem Cell Reports *6*.

Haenseler, W., Sansom, S.N., Buchrieser, J., Newey, S.E., Moore, C.S., Nicholls, F.J., Chintawar, S., Schnell, C., Antel, J.P., Allen, N.D., et al. (2017). A Highly Efficient Human Pluripotent Stem Cell Microglia Model Displays a Neuronal-Co-culture-Specific Expression Profile and Inflammatory Response. Stem Cell Reports *8*, 1727–1742.

Hansen, M., Varga, E., Aarts, C., Wust, T., Kuijpers, T., von Lindern, M., and van den Akker, E. (2018). Efficient production of erythroid, megakaryocytic and myeloid cells, using single cell-derived iPSC colony differentiation. Stem Cell Res. *29*, 232–244.

Kambal, A., Mitchell, G., Cary, W., Gruenloh, W., Jung, Y., Kalomoiris, S., Nacey, C., McGee, J., Lindsey, M., Fury, B., et al. (2011). Generation of HIV-1 resistant and functional macrophages from

hematopoietic stem cell-derived induced pluripotent stem cells. Mol. Ther. *19*, 584–593.

Karlsson, K.R., Cowley, S., Martinez, F.O., Shaw, M., Minger, S.L., and James, W. (2008). Homogeneous monocytes and macrophages from human embryonic stem cells following coculture-free differentiation in M-CSF and IL-3. Exp. Hematol. *36*, 1167–1175.

Klimchenko, O., Di Stefano, A., Geoerger, B., Hamidi, S., Opolon, P., Robert, T., Routhier, M., El-Benna, J., Delezoide, A.L., Boukour, S., et al. (2011). Monocytic cells derived from human embryonic stem cells and fetal liver share common differentiation pathways and homeostatic functions. Blood *117*, 3065–3075.

Lachmann, N., Ackermann, M., Frenzel, E., Liebhaber, S., Brennig, S., Happle, C., Hoffmann, D., Klimenkova, O., Lüttge, D., Buchegger, T., et al. (2015). Large-scale hematopoietic differentiation of human induced pluripotent stem cells provides granulocytes or macrophages for cell replacement therapies. Stem Cell Reports *4*, 282–296.

Law, C.W., Chen, Y., Shi, W., and Smyth, G.K. (2014). Voom: Precision weights unlock linear model analysis tools for RNA-seq read counts. Genome Biol. *15*, 1–17.

Lee, C.Z.W., Kozaki, T., and Ginhoux, F. (2018). Studying tissue macrophages in vitro: are iPSC-derived cells the answer? Nat. Rev. Immunol. *18*, 716–725.

Lindmark, H., Rosengren, B., Hurt-Camejo, E., and Bruder, C.E.G. (2004). Gene expression profiling shows that macrophages derived from mouse embryonic stem cells is an improved in vitro model for studies of vascular disease. Exp. Cell Res. *300*, 335–344.

Monkley, S., Krishnaswamy, J.K., Goransson, M., Clausen, M., Mueller, J., Thorn, K., Hicks, R., Delaney, S., and Stjernborg, L. (2020). Optimised generation of iPSC-derived macrophages and dendritic cells that are functionally and transcriptionally similar to their primary counterparts. PLoS One 1–17.

Moore, K.J., Fabunmi, R.P., Andersson, L.P., and Freeman, M.W. (1998). In Vitro–Differentiated Embryonic Stem Cell Macrophages. Arterioscler. Thromb. Vasc. Biol. *18*, 1647–1654.

Mucci, A., Kunkiel, J., Suzuki, T., Brennig, S., Glage, S., Kühnel, M.P., Ackermann, M., Happle, C., Kuhn, A., Schambach, A., et al. (2016). Murine iPSC-Derived Macrophages as a Tool for Disease Modeling of Hereditary Pulmonary Alveolar Proteinosis due to Csf2rb Deficiency. Stem Cell Reports *7*, 292–305.

Mukherjee, C., Hale, C., and Mukhopadhyay, S. (2018). A Simple Multistep Protocol for Differentiating Human Induced Pluripotent Stem Cells into Functional Macrophages. In Macrophages: Methods and Protocols, G. Rousselet, ed. (New York, NY: Springer New York), pp. 13–28.

Nakano, T., Kodama, H., and Honjo, T. (1994). Generation of lymphohematopoietic cells from embryonic stem cells in culture. Science (80-.). *265*, 1098–1101.

Odegaard, J.I., Vats, D., Zhang, L., Ricardo-Gonzalez, R., Smith, K.L., Sykes, D.B., Kamps, M.P., and Chawla, A. (2007). Quantitative expansion of ES cell-derived myeloid progenitors capable of differentiating into macrophages. J. Leukoc. Biol. *81*, 711–719.

Rajab, N., Rutar, M., Laslett, A.L., and Wells, C.A. (2018). Designer macrophages: Pitfalls and opportunities for modelling macrophage phenotypes from pluripotent stem cells. Differentiation *104*, 42–49.

Salvagiotto, G., Burton, S., Daigh, C.A., Rajesh, D., Slukvin, I.I., and Seay, N.J. (2011). A defined, feeder-free, serum-free system to generate In Vitro hematopoietic progenitors and differentiated blood cells from hESCs and hiPSCs. PLoS One *6*.

Schwartz, M.P., Hou, Z., Propson, N.E., Zhang, J., Engstrom, C.J., Costa, V.S., Jiang, P., Nguyen, B.K., Bolin, J.M., Daly, W., et al. (2015). Human pluripotent stem cell-derived neural constructs for predicting neural toxicity. Proc. Natl. Acad. Sci. U. S. A. *112*, 12516–12521.

Senju, S., Haruta, M., Matsunaga, Y., Fukushima, S., Ikeda, T., Takahashi, K., Okita, K., Yamanaka, S., and Nishimura, Y. (2009). Characterization of dendritic cells and macrophages generated by directed differentiation from mouse induced pluripotent stem cells. Stem Cells *27*, 1021–1031.

Senju, S., Haruta, M., Matsumura, K., Matsunaga, Y., Fukushima, S., Ikeda, T., Takamatsu, K., Irie, A., and Nishimura, Y. (2011). Generation of dendritic cells and macrophages from human induced pluripotent stem cells aiming at cell therapy. Gene Ther. *18*, 874–883.

Soneson, C., Love, M.I., and Robinson, M.D. (2016). Differential analyses for RNA-seq: Transcript-level estimates improve gene-level inferences [version 2; referees: 2 approved]. F1000Research *4*, 1–22.

Srivastava, A., Malik, L., Sarkar, H., Zakeri, M., Almodaresi, F., Soneson, C., Love, M., Kingsford, C., and Patro, R. (2019). Alignment and mapping methodology influence transcript abundance estimation. BioRxiv 1–22.

Subramanian, A., Guo, B., Marsden, M.D., Galic, Z., Kitchen, S., Kacena, A., Brown, H.J., Cheng, G., and Zack, J.A. (2011). Macrophage differentiation from embryoid bodies derived from human embryonic stem cells. Hum. Mesenchymal Embryonic Stem Cells *4*, 211–232.

Takata, K., Kozaki, T., Lee, C.Z.W., Thion, M.S., Otsuka, M., Lim, S., Utami, K.H., Fidan, K., Park, D.S., Malleret, B., et al. (2017). Induced-Pluripotent-Stem-Cell-Derived Primitive Macrophages Provide a Platform for Modeling Tissue-Resident Macrophage Differentiation and Function. Immunity *47*, 183-198.e6.

Uenishi, G., Theisen, D., Lee, J.H., Kumar, A., Raymond, M., Vodyanik, M., Swanson, S., Stewart, R., Thomson, J., and Slukvin, I. (2014). Tenascin C promotes hematoendothelial development and T lymphoid commitment from human pluripotent stem cells in chemically defined conditions. Stem Cell Reports *3*, 1073–1084.

Vodyanik, M.A., Bork, J.A., Thomson, J.A., and Slukvin, I.I. (2005). Human embryonic stem cell-derived CD34+ cells: Efficient production in the coculture with OP9 stromal cells and analysis of lymphohematopoietic potential. Blood *105*, 617–626.

Wiles, M. V., and Keller, G. (1991). Multiple hematopoietic lineages develop from embryonic stem (ES) cells in culture. Development *111*, 259–267.

van Wilgenburg, B., Browne, C., Vowles, J., and Cowley, S.A. (2013). Efficient, Long Term Production of Monocyte-Derived Macrophages from Human Pluripotent Stem Cells under Partly-Defined and Fully-Defined Conditions. PLoS One *8*.

Yanagimachi, M.D., Niwa, A., Tanaka, T., Honda-Ozaki, F., Nishimoto, S., Murata, Y., Yasumi, T., Ito, J., Tomida, S., Oshima, K., et al. (2013). Robust and Highly-Efficient Differentiation of Functional Monocytic Cells from Human Pluripotent Stem Cells under Serum- and Feeder Cell-Free Conditions. PLoS One *8*, 1–9.

Zhang, H., Xue, C., Shah, R., Bermingham, K., Hinkle, C.C., Li, W., Rodrigues, A., Tabita-Martinez, J., Millar, J.S., Cuchel, M., et al. (2015). Functional Analysis and Transcriptomic Profiling of iPSC-Derived Macrophages and Their Application in Modeling Mendelian Disease. Circ. Res. *117*, 17–28.

Zhuang, L., Pound, J.D., Willems, J.J.L.P., Taylor, A.H., Forrester, L.M., and Gregory, C.D. (2012). Pure populations of murine macrophages from cultured embryonic stem cells. Application to studies of chemotaxis and apoptotic cell clearance. J. Immunol. Methods *385*, 1–14.
